# Systematic literature review: treatment of postural orthostatic tachycardia syndrome (POTS)

**DOI:** 10.1007/s10286-025-01172-2

**Published:** 2025-11-12

**Authors:** Nicole Schiweck, Katharina Langer, Andrea Maier, Daniel Vilser, Juliane Spiegler

**Affiliations:** 1 Department of Pediatrics, Universitätsklinikum Würzburg, Josef-Schneider-Str. 2, 97080 Würzburg, Germany; 2 MRI Munich Chronic Fatigue Center for Young People (MCFC), Technical University of Munich, Ismaninger Str. 22, 81675 Munich, Germany; 3 Department of Neurology, University Clinic RWTH Aachen, Pauwelsstraße 30, 52074 Aachen, Germany; 4 Clinic for Pediatrics, Müller-Gnadenegg-Weg 4, 86633 Neuburg an Der Donau, Germany

**Keywords:** Postural orthostatic tachycardia syndrome, Chronic fatigue syndrome, Therapy, Children, Adults, Cardiology

## Abstract

Postural orthostatic tachycardia syndrome (POTS) is a condition defined by symptoms of orthostatic intolerance and a sustained heart rate (HR) increment of ≥ 30 beats per minute (bpm) upon postural change to the upright position in the absence of orthostatic hypotension, defined as a sustained decrease in systolic blood pressure (SBP) of ≥ 20 mmHg or a decrease in diastolic blood pressure (DBP) of ≥ 10 mmHg within 3 min of standing. In children, a sustained HR increment of at least 40 bpm is required for diagnosis of POTS. POTS is a common condition in adults and children suffering from myalgic encephalomyelitis/chronic fatigue syndrome (ME/CFS). In daily clinical practice, therapeutic recommendations are rare and evidence is missing. The objective of this review is to present the current knowledge on non-pharmacological and pharmacological approaches in POTS with a special focus on POTS therapy in children and people with ME/CFS. Of 3853 studies, 45 studies were included in the systematic review. Evidence on therapy in POTS is rare and large randomized controlled trials (RCT) on single interventions are needed. Non-pharmacological approaches such as the use of compression garments, physical training, salt supplementation and transdermal vagal nerve stimulation could be possible treatment options in POTS because they are easy to implement as first-line therapeutic measures in clinical practice. For pharmaceuticals, several studies showed significant effects following therapy with ivabradine and β-adrenergic blocking agents. There are single studies which imply that midodrine (hydrochloride) and pyridostigmine seem to have a beneficial effect on hemodynamics in POTS.

**Supplementary Information:**

The online version contains supplementary material available at 10.1007/s10286-025-01172-2.

## Introduction

Postural orthostatic tachycardia syndrome (POTS) is a condition defined by symptoms of orthostatic intolerance, such as dizziness, headache, blurred vision, nausea or syncope for at least 3 months occurring with a sustained heart rate (HR) increment of ≥ 30 (≥ 40 bpm for people up to 19 years) upon postural change to the upright position in the absence of orthostatic hypotension (OH) defined as a sustained decrease in systolic blood pressure (SBP) of ≥ 20 mmHg or a sustained decrease in diastolic blood pressure (DBP) of ≥ 10 mmHg within 3 min of standing. For the diagnosis of POTS, a history of orthostatic intolerance (OI) is usually required to persist for a minimum of 3 months and common diseases which can cause similar symptoms, such as endocrinological diseases, have to be ruled out [1]. The prevalence of POTS is estimated at 0.2% of all individuals [2] which equals approximately 160,000 people in Germany. POTS and certain types of orthostatic intolerance often occur in patients suffering from myalgic encephalomyelitis/chronic fatigue syndrome (ME/CFS) [3] who usually require extensive medical care. According to several studies, the prevalence of POTS in ME/CFS is estimated between 11% [4] and 25%, but could be even higher because of a lack of diagnostic tests in clinical practice and sensitivity of some testing methods [5]. One of the current definitions of ME/CFS was published by the Institute of Medicine (IOM) in 2015, nowadays known as the National Academy of Medicine (NAM). The diagnostic IOM criteria require a substantially reduced ability to participate in social and educational activities, the occurrence of post-exertional malaise (PEM) together with unrefreshing sleep and either cognitive impairment or orthostatic intolerance [6]. In clinical practice and research, the Canadian Consensus Criteria (CCC) reported by Carruthers et al. [7] are often used together with the IOM criteria or alternatively for the clinical diagnosis of ME/CFS. In particular, children and adolescents with ME/CFS show a high comorbidity rate with POTS [8]. Data about therapeutic options are rare, and official recommendations are missing, making daily clinical practice challenging for doctors and patients.

The objective of this review is to present the current state of knowledge on non-pharmacological and pharmacological treatment options in POTS with a special focus on therapeutic approaches in children. Furthermore, we aim to mark out and discuss possible treatment options for patients with ME/CFS.

## Materials and methods

This review was registered on PROSPERO in May 2024 (CRD42024507731). The literature research strategy was developed in cooperation with the University Library Wuerzburg, Germany and can be viewed in the supplementary material (Supplement 1). PubMed, Cochrane, Embase and ClinicalTrials.gov were searched on May 15, 2024. Data published before May 15, 2024 was considered for abstract screening. As a result of limited resources, we excluded studies in languages other than English or German. We followed PRISMA guidelines with two independent reviewers (NS, KL) at all steps of screening, data extraction and quality assessment. If consensus could not be reached, a third reviewer (JS) was consulted. A list of all studies found on ClinicalTrials.gov for which results were not available can be found in the supplementary material (Supplement 2).

Types of studies: We included randomized controlled trials (RCT) and non-randomized trials (non-RCT) which were assessed separately. Clinical case reports were excluded. We did not restrict inclusion by number of participants.

Types of participants: Adults and/or children with a confirmed diagnosis of POTS using definitions stated above were eligible for inclusion.

Types of interventions: We screened for all types of non-pharmacological and pharmacological interventions, given by any route of administration, in any dose and frequency of application.

Types of comparators/controls: For non-RCTs, pre- vs. post-intervention analyses within the POTS study groups served as a comparator. For RCTs, either a placebo-controlled or a standard-of-care (SOC) approach was used as a comparator.

Main outcome measures: For symptom burden the Vanderbilt Orthostatic Symptom Scale (VOSS) and COMPASS-31 were considered, for quality of life the Short-Form 36 and SF-36. Changes in symptom burden which were assessed by other symptom scores were reported without* p* values. Change in HR upon postural change was considered as well. We differentiated physiological changes, such as changing from a supine to a standing position and provoked orthostatic changes, which can be found during tilt table tests with a head-up tilt (HUT). If data on HR increment was not available, we assessed upright HR upon postural change.

Risk of bias assessment was carried out using the RoB 2 tool (2019) for RCTs (Supplement 4) and ROBINS-I (original version 2016)) for non-RCT (Supplement 5).

Measures of treatment effect: For binary data, treatment effects are presented as risk ratios (RR) with 95% confidence intervals. For continuous data, mean difference (MD) and standard deviation (SD) are given.* P* values < 0.05 were considered to demonstrate a statistical significance.

Certainty of evidence was determined for each intervention, using the GRADE approach [9].

## Results

**Fig. 1:**
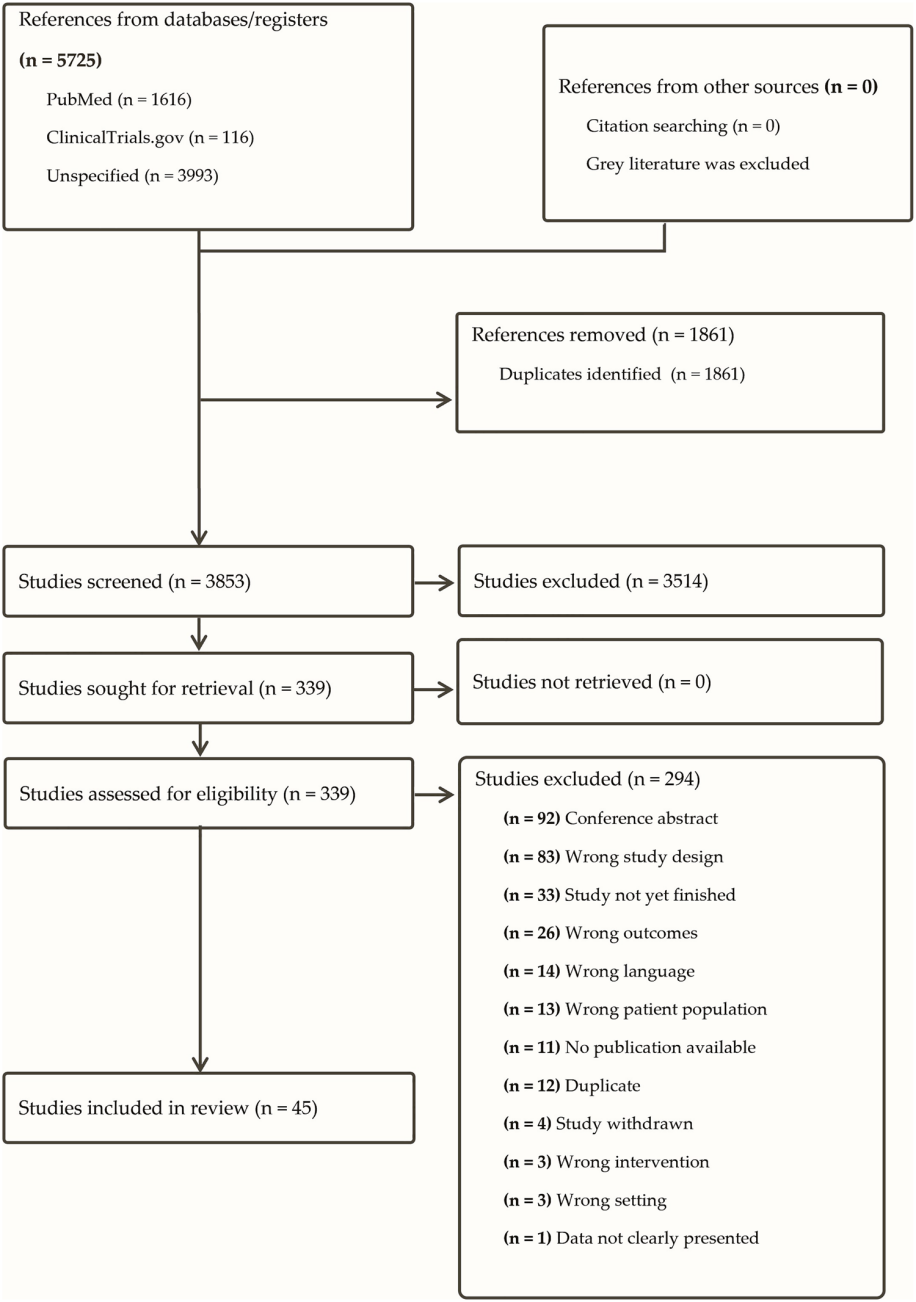
Summary of the study selection process

Of 3853 studies, a total of 45 studies qualified for data extraction and analysis and were included in the systematic review. Eighteen RCTs and 27 non-RCTs were analysed. Figure 1 shows the detailed process of study selection following PRISMA guidelines. Study characteristics of all included studies can be found in Supplement 3. A list of studies excluded during full-text screening can be found in the supplementary material (Supplement 6).

### Non-pharmacological interventions

In total, seven RCT and 13 non-RCTs assessing non-pharmacological therapeutic options in POTS were analysed. Interventions include breathing devices, compression garments, physical exercise, salt supplementation, transdermal vagus nerve stimulation (tVNS), increased oral water intake, intravenous saline infusions and gluten-free diet.

#### Breathing devices

In a single-blinded, crossover RCT, the short-term use of an impedance threshold device (ITD) in adult patients (*n* = 26) was compared to the use of a sham device. After 10 min of HUT testing, ITD caused a significant decrease in HR upon HUT to 70° compared to the use of a sham device (*p* = 0.007). However, there was no significant change in symptom burden assessed by VOSS (*p* = 0.897) [10]. There was no data on the use of breathing devices in patients with ME/CFS.

#### Compression garments

One RCT with 19 female patients aged 32 ± 2 years in which only splanchnic venous compression (40 mmHg) was compared to placebo could not find significant changes in HR increment (*p* > 0.05) upon postural change to standing or symptom burden (VOSS) after 2 h [11]. In their second protocol, the same group compared the use of 20 mg propranolol to abdominal compression combined with 20 mg propranolol, but did not find any differences in standing HR 2 h after drug application. In adult patients (*n* = 30) aged 18–60 years, Bourne et al. showed in a crossover RCT that the application of both abdominal and leg compression garments (20–40 mmHg) led to a significant decrease in HR increment upon HUT compared to no compression (*p* < 0.001) [12]. Additionally, symptom burden assessed by VOSS was reduced (*p* < 0.001). Nardone et al. used a crossover RCT to investigate whether a neck compression collar reduces upright HR during HUT and symptom burden in patients with POTS (*n* = 10) and found a significant decrease in symptom burden (VOSS) in adult patients (40 ± 10 years) (*p* = 0.04). Neck compression did not have any impact on upright HR (*p* = 0.49) [13].

One prospective non-RCT showed that in patients aged 13–19 years (*n* = 20), short-term abdominal compression (20–40 mmHg) led to a significant reduction in HR increment upon HUT to 70° (*p* < 0.001) [14]. Patients reported an improvement in symptom burden.

There was no study on the use of compression garments with explicit data on ME/CFS.

#### Physical training

Six studies tested the impact of physical exercise on hemodynamics or symptom burden in POTS of which one was an RCT and five were non-RCTs. Subjects participated in semi- or unsupervised training programs for 3–6 months.

Wheatley-Guy et al. conducted an RCT in adults conducting a partly supervised moderate intensity aerobic training with a target of 20–30 min three times a week [15]. They did not find any relevant changes in symptom burden assessed by COMPASS-31 after a total of 3 months in the intervention group (*n* = 26) compared to a SOC control group (*n* = 23) (*p* = 0.0925).

In non-RCTs with training programs lasting for up to 6 months, significant improvements in hemodynamics upon postural change were found in adult patients (*n* = 48) (*p* < 0.01), reducing HR (mean ± SD) increment from 36 ± 9 to 27 ± 7 bpm during a postural change from the supine position to standing, after participants had worked out unsupervised for up to six times a week [16]. Certain symptoms of orthostatic tachycardia improved but symptom burden was not assessed by any of the scores named above. Studies in 19 adult patients by Shibata et al. and Fu et al. showed that 30–45-min sessions amounting to 5–6 h/week of endurance training plus weekly weight lifting also showed a reduction in HR upon HUT to 60° (*p* < 0.05; *p* = 0.02) [17, 18]. However, no statement on symptom burden was made. In another non-RCT, George et al. found that 3 months of physical endurance training of 30–40 min 3–6 times per week plus weight lifting significantly improves physical, mental and social components of life quality (*p* < 0.001), assessed by SF-36 in 78 adult patients [19]. A current study by Svensson et al. presented a significant decrease in symptom burden in adult patients (*n* = 25) with a reduction in VOSS from 41.4 ± 17.1 to 32.9 ± 19.1 (mean ± SD) (*p* = 0.017) after individualized training [20].

The studies on physical training did not stratify by ME/CFS or other comorbidities.

#### Salt supplementation

In a crossover RCT, Garland et al. assessed the change in HR from supine to the standing in 14 patients with POTS aged 23–49 years undertaking a high-sodium (300 mEq/day) vs. a low-sodium (10 mEq/day) diet for 6 days and additionally compared the results to those of 13 healthy controls (HC) [21]. There was a significant reduction in HR increment in the high-sodium group in the upright position, with a median of 46 (interquartile range (IQR) 32–55) compared to the low-sodium group with a median of 60 (IQR 55–64). Moreover, in a comparison of subjects with POTS to HC, the reduction in HR was markedly greater in POTS (*p* < 0.001). Within the POTS group, there was no significant difference in symptom burden assessed by VOSS between high-sodium and low-sodium dietary approaches. No specific data on salt supplementation in patients with ME/CFS was found.

#### Transdermal vagus nerve stimulation

Stavrakis et al. performed an RCT to investigate the effect of tVNS positioned at the tragus on hemodynamics and symptom burden in adult patients with POTS [22]. Eleven participants (aged 31.2 ± 5.5 years) performed tVNS for 1 h/day over 4 months and their results were compared to 14 participants aged 30.8 ± 5.6 years using a sham device for the same amount of time. There were no significant differences between groups at baseline. After 4 months, HR increment following HUT was significantly reduced in the intervention group (17.6 ± 9.9 bpm) compared to the control group (31.7 ± 14.4 bpm, *p* = 0.01). However, tVNS did not cause a marked change in symptom burden which was assessed by COMPASS-31 (*p* = 0.07). No study specifically addressed tVNS in patients with POTS and ME/CFS.

#### Increased oral water intake

Three non-RCTs that analysed the short-term effects of increased water intake on hemodynamics were included in this review. Z’Graggen et al. did not detect any significant changes in HR increment 5 min after HUT following oral hydration with 450 ml water or clear soup (*p* > 0.05) in adult patients (*n* = 7) [23]. They did not perform any structured assessment of symptoms. Other trials on oral water intake (500 ml) also showed no effect on HR increment upon HUT (*p* = 0.098) in patients (*n* = 8, aged 18–45 years) compared to control [24] or HR upon tilt (*n* = 13, aged 27.62 ± 5.95) [25]. These studies reported improved symptom burden after water intake but did not use any of the scales listed above. In all studies analysed, we did not find any data to specifically address the condition of POTS in ME/CFS.

#### Intravenous saline infusions

Three non-RCTs investigated the effect of short-term [26, 27] and long-term, intermittent application of intravenous saline [28]. A one-time saline infusion (1 L) caused a reduced HR increment from the supine position to standing in 13 adult patients compared to baseline (*p* < 0.001) [27]. However, in another study, saline did not lead to relevant changes regarding upright HR in patients (*n* = 11) aged 14–39 (*p* = 0.328) after 5 min of HUT but patients reported improved symptom burden [26]. Ruzieh et al. performed intermittent, intravenous saline infusions (1 L) with possible increase to 2 L per week or decrease to 1 L every 2–4 weeks in 57 adult patients over a period of 3–12 months which caused a significant change in quality of life with a change of 19.1 ± 2.7 (mean ± SD) assessed by SF-36 (*p* < 0.001) [28]. There was no explicit data on the use of intravenous saline in patients with ME/CFS.

#### Gluten-free diet

Zha et al. retrospectively assessed symptom burden via COMPASS-31 in female patients with POTS (aged 16–62 years) with a diagnosis of celiac disease ruled out who had followed a gluten-free diet (GFD) for 4 weeks. Compared to baseline, a significant decrease in symptom burden was detected (*p* < 0.00001), with major improvements in components of orthostatic intolerance (*p* < 0.00001) and vasomotor aspects (*p* = 0.0034) [29]. This study did not stratify its population by ME/CFS or other comorbidities.

### Pharmacological interventions

In total, 12 RCTs and 16 non-RCTs assessing pharmacological therapeutic options in POTS were analysed. Interventions included β-adrenergic blocking agents, pyridostigmine, desmopressin, ivabradine, midodrine, modafinil, intravenous immunoglobulins (IVIG), atomoxetine, melatonin, selective serotonin reuptake inhibitors (SSRI), bupropion, droxidopa, erythropoietin, clonidine, digoxin and octreotide.

#### β-Adrenergic blocking agents

In this review, three RCTs and four non-RCTs which investigated the effect of β-adrenergic blocking agents on hemodynamics, symptom burden and quality of life in POTS were evaluated.

One crossover RCT on short-term application of β-adrenergic blocking agents (20 mg propranolol) vs. placebo showed a significant decrease in HR increment (*p* = 0.028) from the seated to the standing position and symptom burden (*p* < 0.007) assessed via VOSS in adult patients (*n* = 54, aged 34 ± 10 years) [30]. Smith et al. found significant changes in upright HR (*p* < 0.05) but not in HR increment (*p* > 0.05) from sitting to standing between intervention (20 mg propranolol) and control (placebo) 2 h after drug administration in 19 female patients aged 32 ± 2 years. There were no remarkable changes in symptom burden which was assessed by VOSS (*p* = 0.477) [11].

Moon et al. performed an RCT in adult patients, of which 19 (aged 39.4 ± 11.6 years) received 10–20 mg propranolol twice daily and 17 (aged 29.8 ± 9.9 years) received 2.5–5 mg bisoprolol once daily over a period of 3 months. Compared to baseline data, there was a significant decrease in HR increment from 39.3 ± 13.7 to 15.0 ± 8.4 (*p* < 0.001) with propranolol and 42.6 ± 9.9 to 14.2 ± 6.2 (*p* < 0.001) with bisoprolol after changing from supine to the standing position. Additionally, scores for physical and mental components of life quality (SF-36) showed significant improvement in both groups (*p* < 0.001) [31].

Four non-RCTs investigated the short- and long-term effects of β-adrenergic blocking agents. Single application of 40 mg propranolol in nine patients with POTS aged 14–39 years caused a significant decrease in HR after 5 min of HUT (*p* < 0.0002) compared to pooled baseline data [26]. However, propranolol did not cause any significant changes in symptom burden, for which they used a verbal reporting system.

Freitas et al. tested the effect of 5 mg bisoprolol (once daily) in ten adult participants who received medication over a period of 3 months. This led to a reduced HR upon HUT in patients (*p* < 0.05). Seven patients verbally reported improvement of orthostatic symptoms. Three patients without significant improvement in symptoms were additionally prescribed fludrocortisone after 6 weeks. After 12 weeks all patients were asymptomatic [32].

In 49 children, the application of metoprolol (0.5 mg/kg body weight, two times per day) significantly attenuated HR increment from the supine to the standing position after 1.5–3 months (*p* < 0.001) and led to an improvement in symptoms [33]. Chen et al. analysed hemodynamics in children (*n* = 19) who received metoprolol (0.5 mg/kg, two times per day) for 3–6 months. Patients presented a reduced HR increment upon standing compared to their pre-treatment state (*p* < 0.05) and also reported improved symptom burden [34].

No data on the use of β-adrenergic blocking agents in patients with POTS and ME/CFS was found.

#### Acetylcholinesterase inhibitors (pyridostigmine)

Three studies on the effect of pyridostigmine in POTS were included in this review, of which one was designed as an RCT.

Raj et al. found significant changes in standing HR after 2 h (*p* < 0.001) but not after 4 h (*p* = 0.160) following one single application of 30 mg pyridostigmine compared to placebo in adult patients (*n* = 15, aged 37 ± 11 years) in a crossover RCT. There were no significant changes in symptom burden assessed by VOSS (*p* = 0.174) [35].

In a non-RCT, Stewart et al. showed a reduction in HR upon HUT after one-time application of 60 mg pyridostigmine in 36 patients compared to no treatment (*p* < 0.05) [36]. This study focused on hemodynamics and did not use scores to assess orthostatic symptoms.

Kanjwal et al. retrospectively analysed long-term therapy with pyridostigmine (from 60 mg up to 180 mg per day) in 172 patients with POTS aged 26 ± 12 years. After 12 ± 3 months, there was a significantly reduced standing HR (*p* = 0.003) compared to pre-treatment state. Patients reported improvement in certain symptoms, such as fatigue or palpitations but no formal questionnaire was used to assess symptom burden [37].

No study specifically addressed the use of pyridostigmine in patients with ME/CFS.

#### Desmopressin (DDAVP)

Coffin et al. used a single-blinded crossover RCT to investigate the effect of desmopressin (0.2 mg) on hemodynamics and symptom burden in POTS. In 30 adult patients (aged 37 ± 11 years), a single application of desmopressin did not cause any relevant changes in HR increment from sitting to standing 4 h after drug administration compared to placebo (*p* = 0.181). However, symptom burden assessed via VOSS was remarkably reduced to a score of 13 (intervention) vs. 19 (control) (*p* = 0.009) [38]. There was no data available for the use of desmopressin in patients suffering from ME/CFS.

#### Ivabradine

Ivabradine is a funny channel inhibitor that is usually used in patients with chronic heart failure. Four studies on the usage of ivabradine in POTS were evaluated in this review, including one RCT and three non-RCTs.

In a double-blinded, crossover RCT (*n* = 22, aged 33.9 ± 11.7 years), Taub et al. found that application of 5 mg ivabradine twice daily for 1 month reduced HR increment upon HUT in the intervention group compared to placebo (*p* < 0.001). Furthermore, physical and social functioning improved under ivabradine, assessed by Short-Form 36 (SF-36) (*p* = 0.008 and *p* = 0.021) [39].

A non-RCT showed that one single short-term application of 7.5 mg ivabradine significantly reduced HR upon HUT in eight adult patients but no information on symptom burden following intervention was given [40].

In a retrospective study of 49 adult patients with POTS who received 2.5–10 mg ivabradine twice daily over a period of 3–12 months, standing HR was reduced from 107.4 ± 14.1 bpm (mean ± SD) to 95.1 ± 13.7 bpm (*p* < 0.001) [41]. Certain symptoms, such as palpitations and lightheadedness improved under therapy, but no structured questionnaire was used to depict symptom burden.

Towheed et al. conducted a retrospective study in 27 children (12–17 years old) with confirmed diagnosis of POTS [42]. Three to 12 months after the start of ivabradine therapy, which was started at 2.5–5 mg twice daily and, if tolerated, titrated to 7.5 mg twice daily standing, HR was significantly decreased from 100.5 ± 18.1 to 80.9 ± 10.1 bpm (*p* < 0.05). Symptoms of orthostatic intolerance were assessed separately without the use of a scoring system. Palpitations, fatigue, (pre-)syncopes and lightheadedness were reduced under therapy.

There was no scientific data on the specific use of ivabradine in patients with ME/CFS.

#### Midodrine

Midodrine (hydrochloride) serves as a selective α1-adrenoreceptor agonist. Its effect on POTS was evaluated in seven of the included studies.

Ross et al. performed an RCT in teenage patients (12–20 years) [43]. Their study distinguished two subtypes of POTS, namely hyperadrenergic (*n* = 8) or neuropathic (*n* = 12) POTS. The condition was defined as hyperadrenergic POTS if patients had an arterial calf blood flow less than 1.2 ml/100 ml per min (low flow) and the term neuropathic POTS was used if patients had an arterial calf blood flow greater than 1.2 ml/100 ml per min. Neuropathic POTS is a condition with small-fibre neuropathy patterns which can lead to a reduced vasoconstriction while hyperadrenergic POTS is dominated by increased noradrenaline levels [44]. The study was performed in a crossover manner. Each participant received a therapy with 2.5–10 mg midodrine three times daily for 2 weeks with a 1-week washout period between placebo trials. After 2 weeks of midodrine, patients with neuropathic POTS had a significantly reduced HR upon HUT (*p* = 0.0002) compared to placebo, while there was no major effect of midodrine in hyperadrenergic POTS (*p* = 0.3). There was no structural assessment of symptom burden or quality of life.

In studies performed by Jacob et al. [27] and Hoeldtke et al. [45], after a single application of 5–10 mg midodrine, HR increment during a standing test in adult patients was significantly attenuated (*p* < 0.05) and standing HR was remarkably lower (*p* < 0.001). Jacob et al. reported an overall improvement in orthostatic symptoms which were informally assessed [27].

However a non-RCT reported by Gordon et al. [26] did not find significant changes in HR upon HUT in patients (*n* = 12, aged 14–39) 5 min after one-time application of 10 mg midodrine compared to pooled baseline data (*p* = 0.146). Despite these findings, patients reported improved symptom severity.

Three non-RCTs investigated the effect of midodrine in children. Chen et al. showed that in 19 children, daily administration of 2.5 mg midodrine over 3–6 months caused a significant reduction in HR increment upon standing (*p* < 0.05) and symptom burden was markedly reduced but assessed without any of the questionnaires listed above [34]. A similar result during HUT was published by Zhang et al. [46]. Upon midodrine administration (2.5 mg once daily) over 3 months, in 44 children, HR increment following postural change was significantly attenuated (*p* = 0.016). Children who responded to treatment reported a reduction in overall symptom burden. A third trial tested 28 children who received 2.5 mg midodrine once daily for up to 8 months and HR increment upon HUT was reduced from 39.7 ± 10.1 to 26.2 ± 6.7 bpm (*p* < 0.001) [47]. This also led to a decrease in symptom burden, but none of the structural questionnaires named above was used. None of these studies stratified their population by ME/CFS or other comorbidities.

#### Modafinil

An RCT was conducted by Kpaeyeh et al. [48] to assess changes in hemodynamics and symptom burden (VOSS) after one-time application of 100 mg modafinil in 54 patients with POTS aged 32 ± 10 years. There were no significant changes in standing HR (*p* = 0.139) or symptom burden which was assessed by VOSS (*p* = 0.962), 4 h after administration of modafinil. There was no study on the use of modafinil in patients with ME/CFS.

#### Intravenous immunoglobulins (IVIG)

Vernino et al. [49] compared the effects of IVIG to albumin infusions on symptom burden in POTS. Thirty participants aged 18–55 years received either intermittent infusions of IVIG (*n* = 16) or albumin (*n* = 14) in a randomized manner and were asked for symptoms of orthostatic intolerance via VOSS and COMPASS-31 at 3 months. Twenty eight participants completed symptom scores at 3 months. IVIG did not cause significant changes in symptom burden (*p* = 0.927 and *p* = 0.629). The study did not stratify their population by ME/CFS or other comorbidities.

#### Atomoxetine

One RCT on the impact of 40 mg atomoxetine on hemodynamics and symptom burden in POTS was performed by Green et al. [50]. Twenty seven adult participants (34 ± 9 years) received a single dose of atomoxetine. Four hours after administration, atomoxetine did not change HR increment after changing from a seated to a standing position (*p* = 0.508) or symptom burden which was assessed by VOSS (*p* = 0.622). There was no explicit information on the use of atomoxetine in patients with POTS and ME/CFS.

#### Melatonin

Another RCT by Green et al. [51] was conducted in 78 adult patients (32 ± 9 years) with POTS. Four hours after administration of 3 mg melatonin, participants showed a reduced standing HR (*p* = 0.009) but no relevant difference in symptom burden which was assessed by VOSS (*p* = 0.48). There was no specific data for patients with ME/CFS.

#### Selective Serotonin Reuptake Inhibitors (SSRI)

Mar et al. [52] used a randomized, controlled approach to assess the effect of 50 mg sertraline on hemodynamics and symptom burden in 39 patients aged 39 ± 9 years with POTS. However, 4 h after administration, no relevant changes could be detected in HR increment upon standing (*p* = 0.13) or symptom burden assessed by VOSS (*p* = 0.188). A differentiation between patients with POTS alone and other comorbidities was not made in this study.

#### Bupropion

In a chart review of 47 adults (aged 42.0 ± 13.6 years) with POTS, Vyas et al. did not detect any changes in standing HR after treatment with bupropion (150–450 mg once daily) compared to pre-treatment state (*p* = 0.81) [53]. There was no structural assessment of symptom burden in this study and patients did not report overall improvement in symptom severity. There was no specific data on patients with ME/CFS.

#### Droxidopa

Ruzieh et al. retrospectively evaluated the effect of droxidopa on hemodynamics in 37 adult patients who had undergone therapy for up to 12 months [54]. Participants received 100–600 mg droxidopa three times daily. This did not cause any reduction in standing HR (*p* = 0.157). Following treatment, certain symptoms, such as dizziness and fatigue were less frequent but no structural assessment was carried out. This study did not stratify its population by ME/CFS or other comorbidities.

#### Erythropoietin

In a non-RCT, 39 patients (aged 33 ± 12 years) with POTS received 10,000–20,000 IU/week erythropoietin (EPO) depending on their hematocrit [55]. EPO was administered if a patient’s hematocrit was less than 50%. After 6 months of intermittent therapy, standing HR did not show any relevant changes (*p* = 0.5). In this study, no formal questionnaire was used, but patients who responded to treatment showed improved orthostatic symptoms, such as dizziness and palpitations. Specific data for patients with ME/CFS was not available.

#### Octreotide

Hoeldtke et al. tested the impact of subcutaneous application of octreotide in nine patients with POTS [45]. Eight minutes after drug administration standing HR was significantly reduced from 114 ± 0.7 to 90.6 ± 0.78 bpm (*p* < 0.001). There was no structural assessment of symptoms in this study. There was no data on the use of octreotide in patients with ME/CFS.

#### Clonidine

Two non-RCTs which investigated the effect of a one-time application of clonidine on hemodynamics in POTS were included in this review; 0.1–0.2 mg clonidine did not significantly attenuate HR increment when changing to a standing position (*p* > 0.05) [27] or HR during HUT (*p* = 0.481) [26] in 13 patients aged 18–47 and in six patients aged 14–39 years. Informal assessments of symptoms suggest that clonidine does not lead to any improvement in symptom severity, but even deteriorates overall symptom burden. These studies did not include specific data for patients with ME/CFS.

#### Phenobarbital

Gordon et al. showed that one-term application of 120 mg phenobarbital did not significantly change HR upon HUT in 11 patients (aged 14–39) with POTS compared to pooled baseline data (*p* = 0.081) [26]. Phenobarbital did not lead to any improvement in overall symptom burden which was assessed by an informal scoring system. Data on the use of phenobarbital in patients with ME/CFS was not available.

#### Digoxin

In a non-randomized, interventional approach, Stewart et al. [36] did not find any relevant changes in HR upon HUT after one-time application of 500 mg digoxin in 36 patients (aged 15–30 years) compared to no treatment in POTS. There was no statement on changes in symptom burden. In this study, there was no data on the use of digoxin in ME/CFS.

## Discussion

This review presents current evidence on non-pharmacological and pharmacological therapy in patients with POTS. Most therapeutic approaches aim to improve hemodynamics by lowering HR increment or upright HR and by increasing venous return. Several therapeutic approaches have been shown to be possibly effective in lowering HR increment, upright HR and symptom burden in patients with POTS. Our systematic review was conducted in accordance with Cochrane methodological standards, ensuring a high-quality literature search and synthesis. Inclusion criteria were clearly predefined, and two independent reviewers carried out the review process. Any deviations from the protocol, which was published prior to this review, are transparently documented.

When developing the research strategy for this review, we aimed to exclude all studies dealing with non-specific types of orthostatic intolerance. This also applies to studies in which the definition of the investigated condition was insufficient and could not clearly be classified as POTS. Therefore, it cannot be guaranteed that all studies with a different description or non-transparent definition of POTS were found during our literature research process. A list of all excluded studies and the reason for exclusion can be found in the supplementary material.

It must be emphasized that the diagnosis of POTS and its treatment remains a challenge for doctors across the world, especially because diagnostic methods such as tilt table testing or active standing tests are often needed to confirm the diagnosis. Most of the studies analysed in this review use such methods to define their inclusion criteria. These tests are not accessible in all medical institutions because of a lack of equipment, personnel and time constraints. This makes it difficult to diagnose POTS, and study populations do not necessarily represent the majority of patients with POTS. This could explain why large RCTs are missing. A clinically accessible diagnostic scheme that includes functioning and quality of life is needed and should be the subject of future studies.

Several non-pharmacological approaches were assessed in this review. RCT and non-RCTs showed a significant effect on hemodynamics and symptom burden following abdominal compression; in some of the studies this was combined with leg compression. Since compression garments can easily be applied and severe side effects are not to be expected, they might be a possible first-line treatment option for patients with POTS. The effect of long-term use of compression garments in POTS should be the objective of further studies. One RCT showed that salt supplementation improved hemodynamics in patients with POTS [21]. Although no effect on symptom burden could be detected in that study owing to its simple applicability, it should be considered as a possible non-pharmacological treatment option. 

There remains a need for rigorously designed, high-quality studies to clarify the impact of high sodium intake on hemodynamic parameters and symptomatology in individuals with POTS. Physical exercise did not lead to significant changes in POTS in an RCT [15]. However, several non-RCTs with higher, mostly daily frequency of interventions detected significant improvements in hemodynamics and symptom burden in POTS after 3–6 months of physical exercise. These findings need to be assessed in further RCTs and physical exercise should be considered as a possible non-pharmacological intervention in POTS, as it can be individually adapted to the patient’s capabilities. 

In all studies included in this review, there was no effect of increased oral water intake on hemodynamics in POTS, but all of the studies on oral water intake only used a short-term approach and no structural symptom assessment was carried out. However, improved symptom burden is frequently reported by patients in every day practice. Long-term studies on increased oral water uptake are needed to investigate the effects on hemodynamics and symptom burden in POTS, as it should be considered as a possible treatment option owing to its simple applicability. Ruzieh et al. reported a significant improvement in life quality following a long-term intermittent, intravenous saline infusion regime in POTS [28]. However, as a single and invasive method which always carries risks, such as infections, and as a result of the scarce evidence, this intravenous, very individual saline transfusion regime can currently not be recommended as a treatment option. 

We included one RCT on tVNS in POTS. Stavrakis et al. [22] reported improved hemodynamics in POTS after 4 months of daily tVNS compared to placebo. As no significant changes in symptom burden were observed, routine use cannot currently be recommended. However, owing to the promising approach and the non-invasive, easily applicable nature of the method, the effect of tVNS in patients with POTS should be further investigated in future studies. In a first step, available data on HR change under different stimulation modes could be analysed in patients with VNS therapy due to depression or epilepsy. We included one study on the effect of gluten-free dietary approaches in patients with POTS without celiac disease that showed a markedly improved symptom burden after 4 weeks of taking a gluten-free diet. However, that study used a retrospective design which makes it susceptible to recall bias and performing a gluten-free diet can not generally be recommended according to the current state of knowledge. Future RCTs would be helpful to investigate possible effects of dietary approaches in POTS. 

Gamboa et al. tested the impact of ITDs on hemodynamics and symptom burden in POTS and detected a reduction in HR but no significant changes in symptom burden [10]. From a clinical perspective, the use of ITDs is hardly feasible.

Of all the pharmaceuticals that were investigated in this study, β-adrenergic blocking agents have most often been used in studies on POTS. Substances that were used included bisoprolol, metoprolol and propranolol. Throughout all studies, a positive effect on hemodynamics was detected. However, results on symptom burden and quality of life diverged. Since many studies only used a single-time administration approach, more long-term RCTs are needed to assess potential benefits regarding symptom severity. There is abundant experience of using β-adrenergic blocking agents in adults and children and they can be seen as a potential treatment option in POTS if non-pharmacological interventions alone are insufficient. The use of ivabradine as a funny channel inhibitor also led to improved hemodynamics in one RCT and three non-RCTs and was administered to adults and children. Although most studies did not formally assess symptom burden, patients reported improved symptoms, and improved quality of life was detected in an RCT [39]. For ivabradine, long-term studies are also needed to further investigate potential effects in POTS. On the basis of the current evidence, ivabradine could be considered as a pharmacological approach in POTS if non-pharmacological interventions alone are not sufficient. Pyridostigmine was reported to significantly decrease upright HR in POTS during short-term and long-term approaches and informal assessments of symptom burden showed improved symptom severity. Evidence is still scarce and no studies on its use in children were found, so further studies on the long-term use of pyridostigmine in children and adults are essential to assess its effects on POTS. However, pyridostigmine could be considered as a potential treatment option in POTS if non-pharmacological interventions and the use of β-adrenergic blocking agents or ivabradine are not sufficient. In this review, midodrine was used in adults and children in RCT and non-RCTs and results on hemodynamics were divergent between studies. However, midodrine showed a positive effect on hemodynamics and symptom burden in three long-term studies in children and despite the risk of bias that goes along with non-RCTs, these findings should be the motivation to further investigate its effect on POTS in children and adults to assess its potential as a pharmacological intervention. Another substance which needs to be addressed in future studies is desmopressin. Although no relevant effect on hemodynamics was detected by Coffin et al. [38], desmopressin decreased symptom burden which was structurally assessed in an RCT design. As a result of the lack of evidence, desmopressin cannot generally be recommended for patients with POTS but should be the subject of future research. One study each on melatonin (RCT) and octreotide (non-RCT) reported improved hemodynamics in patients with POTS. For melatonin, no significant changes in symptom burden were detected after 4 h [51]. For octreotide, there was no structural assessment of symptoms available [45]. Both substances might be the subject of future research but can currently not be recommended generally. Other pharmaceuticals that were assessed in this review include modafinil, IVIG, droxidopa, erythropoietin, clonidine, atomoxetine, bupropion, phenobarbital, digoxin and SSRI. The studies on these substances investigated the drug’s effect on either hemodynamics or symptom burden or both. No significant changes were found for these parameters. Potential effects of these drugs cannot be ruled out but at the current state of knowledge, these interventions cannot be recommended.

The high heterogeneity of pharmacological and non-pharmacological interventions and outcome assessment tools precluded meta-analysis. The mostly very low certainty of evidence further complicates the interpretation of results. The certainty of evidence of the outcomes included in this systematic review was rated as low or very low. This is primarily due to most outcomes being assessed by only one or two studies with a small number of participants. Furthermore, wide confidence intervals and a high statistical heterogeneity contributed to imprecision and inconsistency of some studies, leading to a downgrading of the certainty of evidence. Additionally, a substantial risk of bias was identified in most studies (Supplements 4 and 5), arising from various methodological limitations in the conduct of the studies. In particular, long-term follow-up has to be interpreted cautiously as some studies allowed continuation of existing therapies/medications and introduction of other interventions (pharmacological and non-pharmacological) for POTS during the study period without controlling for or even reporting these, which leads to a significant risk of bias.

In contrast to the protocol published on PROSPERO, we reduced our main outcomes to symptom burden, quality of life and HR increment or—if information thereon was not given—upright HR, during the data extraction process. This change was based on an interdisciplinary decision, as blood pressure measurements are part of hemodynamic assessments, but—after orthostatic hypotension has been excluded as part of the POTS diagnostic workup—do not typically influence therapeutic decisions in clinical practice. Furthermore, different scores of orthostatic symptom burden were applied in the included studies, but many of them were used by only few institutions. In some studies, symptoms were described verbally without structured evaluation and in these cases could not be objectified. To make results more comparable, we decided to only include tools that are widely used and validated instruments, such as the VOSS or the COMPASS-31 scale, to assess symptom burden and the SF-36 to assess quality of life.

The studies in this review were published from 1997 to 2024. During this time, definitions and diagnostic criteria of POTS have changed. In 2011, Freeman et al. [56] stated that for the diagnosis of POTS, between 12 and 19 years of age, an HR increment of ≥ 40 bpm is required instead of ≥ 30 bpm. This differentiation between adults and children with POTS was insufficient in most studies. Along with that, in studies which include both teenage and adult patients, results were usually not separated into different age groups, making it difficult or impossible to distinguish treatment effects in children and adults. Another aspect to consider is that there are different types of POTS, including hyperadrenergic and neuropathic POTS [57], which might require different treatment approaches because of their distinct pathophysiological mechanisms, yet these distinctions are not generally accounted for during participant selection. In clinical practice, testing for these subtypes is usually not routinely established which might have an impact on response to therapy. In our review, only one study tried to differentiate between POTS subtypes to evaluate treatment response. Different pathological mechanisms have been identified for POTS, such as partial sympathetic denervation, a hyperadrenergic state with a plasma norepinephrine ≥ 600 pg/mL, a norepinephrine transporter deficiency or impaired cerebral autoregulation [44]. These mechanisms need to be further investigated in future studies in order to classify certain subtypes of POTS, and to assess if different treatment approaches are necessary.

POTS is a condition that occurs more often in the female population [2] and participants in clinical trials were predominantly female. However, study results were usually not divided into male and female subgroups. Response to treatment might differ between women and men which makes a differentiation necessary.

As a result of the small amount of RCTs on therapy in POTS, we included non-RCTs and RCTs in this review. Hence, many studies are barely comparable, even if the same intervention was addressed. We did not include case reports although they might include valuable information on side effects and factors that might have an influence on treatment response.

Another aspect that needs to be taken into account is that some of the interventions performed in the included studies are difficult to implement from a clinical point of view, such as the use of ITDs or intermittent saline infusions, because effects might only be temporary and the interventions might be a drain on clinical and the patient’s personal resources. Additionally, with regards to the risks and the cost–benefit ratio, certain interventions, such as weekly saline infusions, do not seem to be acceptable.

Furthermore, in many trials, interventions were performed only once and results depict assessments minutes to few hours after administration. Long-term effects of non-pharmacological and pharmacological therapy in patients with due regard to the pharmaceuticals’ half-lives need to be addressed in future studies. With only one exception [37], all studies had fewer than 100 participants included in their final analysis and therefore provided a very low level of evidence. Additionally, non-RCTs usually have a significant risk of bias due to a lack of blinding or allocation concealment.

It is crucial to point out that we focused on symptom severity and hemodynamics but not all of the studies assessed both outcomes. It is well known that certain pharmacological agents, such as β-adrenergic blocking agents, lead to a reduction in HR. From a clinical perspective, with a reduced HR, the diagnostic criteria of POTS might not be fulfilled after treatment, but patients might still encounter orthostatic symptoms. Symptom burden should always be taken into account when evaluating therapeutic effects of an intervention.

One of our objectives was to find potential treatment options for children. In total, 11 studies which included children and/or teenage patients were included in this review. However, data on POTS in children is rare and its management is not well understood. Some positive effects on hemodynamics or quality of life in children or teenage patients were documented for compression garments, midodrine, β-adrenergic blocking agents, ivabradine and pyridostigmine. These findings should be considered in future RCTs to find suitable treatment options to cover the needs and requirements of young patients.

Another objective was to find treatment options for patients diagnosed with POTS and ME/CFS. These patients have a severely reduced participation in daily educational and social life and often require extensive treatment. POTS is a common condition in patients with ME/CFS [8]. We did not find any studies that specifically addressed the therapy of POTS in ME/CFS. As a result of a significant exertion intolerance in patients with ME/CFS, physical training might increase the risk of PEM and therefore usually needs to be adapted or is not realistic. Further studies are sorely needed to investigate the prevalence of POTS in ME/CFS and to investigate potential treatment strategies which can be individually adapted to a patient’s abilities and needs. Multidisciplinary approaches are needed to address the different phenotypes of ME/CFS. Our work group developed and implemented an online patient education program for patients with ME/CFS, their siblings, their parents and teachers in the past, in order to not only educate people affected but also to investigate challenges and needs of the different groups [58]. Studies like this are needed as a starting point for the development of treatment programs for POTS in ME/CFS. Scientists should be encouraged to stratify their population by comorbidities like ME/CFS, so that more data becomes available on this issue.

All statements in this review focus on data extracted from the studies and might differ from statements made by the authors of the original studies because we focused on certain items of those studies only.

## Conclusions

In this review, we present evidence on possible treatment options in POTS. Compression garments, physical training and salt supplementation have been shown to have beneficial effects on symptom burden and/or hemodynamics in patients with POTS and should be the first therapeutic approach in patients with POTS. Further investigations on the relevance of tVNS are needed to make general recommendations. These non-pharmacological treatment options can always be seen as the foundation of POTS therapy, even when considering pharmaceuticals for treatment.

The use of impedance threshold breathing devices, saline infusions and gluten-free dietary approaches can currently not be recommended and needs to be discussed from a clinical perspective with regards to feasibility, medical resources and those of the patients. Of all the pharmacological substances included in this review, evidence is most widely available for β-adrenergic blocking agents and ivabradine which can be considered as possible treatment options if non-pharmacological management is not sufficient. Pyridostigmine and midodrine might improve hemodynamics in POTS and therefore can be chosen individually as an additional therapeutic approach if non-pharmacological options alone or combined with β-adrenergic blocking agents or ivabradine are insufficient or not suitable. Melatonin and octreotide caused significant changes in HR increment, upright HR and/or symptom burden but can currently not be generally recommended. In children, the use of compression garments can be considered from a non-pharmacological perspective. Pharmaceuticals such as β-adrenergic blocking agents, ivabradine, midodrine and pyridostigmine have been used in children in studies and should be further investigated in future studies as possible treatment options. Evidence is still scarce and further studies on the interventions listed above are needed to provide evidence-based recommendations on the therapeutic procedure. 

On the basis of our results, we suggest that RCTs for pharmacological treatment options of POTS should be designed as long-term studies over at least 3–6 months of treatment with documented changes in HR, a validated symptom score and quality of life assessment to clarify the potential effects of pharmaceuticals. Existing medications and non-pharmacologic interventions need to be controlled for. The studies should be powered to allow differentiation of sex- and age-dependent effects. Studies which specifically address therapeutic options in patients with POTS and ME/CFS are essential. According to our results, effects of midodrine, β-adrenergic blocking agents, ivabradine and pyridostigmine on POTS should be considered. For non-pharmacological treatment options both RCTs and non-RCTs seem suitable to analyse treatments that are dependent on the patient’s preference and adherence to options that are unsuitable for blinding. According to our results compression garments, salt supplementation, physical exercise programs and tVNS might be considered.

Importantly, subgroups, such as children and adolescents, subtypes of POTS, male and female participants, as well as patients with ME/CFS should become a major focus in future research on POTS.

## Supplementary Information

Below is the link to the electronic supplementary material.

Supplementary file1 (DOCX 249 kb)

Supplementary file2 (DOCX 28 kb)

Supplementary file3 (DOCX 37 kb)

Supplementary file4 (DOCX 53 kb)

Supplementary file5 (DOCX 59 kb)

Supplementary file6 (DOCX 87 kb)

Supplementary file7 (DOCX 45 kb)

Supplementary file8 (DOCX 42 kb)

## Data Availability

The data that supports the findings of this systematic review are available in the supplementary material of this article.

## Abbreviations

bpm: Beats per minute
CCC: Canadian Consensus Criteria
COMPASS-31: Composite Autonomic Symptom Score 31
DBP: Diastolic blood pressure
HR: Heart rate
HUT: Head-up tilt
IOM: Institute of Medicine (National Academy of Medicine)
IQR: Interquartile range
ITD: Impedance threshold device
IVIG: Intravenous immunoglobulins
MD: Mean difference
ME/CFS: Myalgic encephalomyelitis/chronic fatigue syndrome
Non-RCT: Non-randomized controlled trial
PEM: Post-exertional malaise
POTS: Postural orthostatic tachycardia syndrome
RCT: Randomized controlled trial
RoB 2: Risk of Bias 2 tool
ROBINS-I: Risk Of Bias In Non-randomized Studies Of Interventions tool
SBP: Systolic blood pressure
SD: Standard deviation
SF-36: Short-Form 36
SOC: Standard of care
tVNS: Transdermal vagus nerve stimulation
VOSS: Vanderbilt Orthostatic Symptom Scale

## References

[1] Raj SR, Guzman JC, Harvey P, Richer L, Schondorf R, Seifer C, Thibodeau-Jarry N, Sheldon RS (2020) Canadian Cardiovascular Society position statement on postural orthostatic tachycardia syndrome (POTS) and related disorders of chronic orthostatic intolerance. Can J Cardiol 36(3):357–372. 10.1016/j.cjca.2019.12.024 32145864 10.1016/j.cjca.2019.12.024

[2] Safavi-Naeini P, Razavi M (2020) Postural orthostatic tachycardia syndrome. Tex Heart Inst J 47(1):57–59. 10.14503/thij-19-7060 32148459 10.14503/THIJ-19-7060 PMC7046364

[3] Natelson BH, Brunjes DL, Mancini D (2021) Chronic fatigue syndrome and cardiovascular disease. JACC 78(10):1056–1067. 10.1016/j.jacc.2021.06.045 34474739 10.1016/j.jacc.2021.06.045

[4] Reynolds GK, Lewis DP, Richardson AM, Lidbury BA (2014) Comorbidity of postural orthostatic tachycardia syndrome and chronic fatigue syndrome in an Australian cohort. J Intern Med 275(4):409–417. 10.1111/joim.12161 24206536 10.1111/joim.12161

[5] Van Campen C, Rowe PC, Visser FC (2018) Low sensitivity of abbreviated tilt table testing for diagnosing postural tachycardia syndrome in adults with ME/CFS. Front Pediatr 6:349. 10.3389/fped.2018.00349 30505831 10.3389/fped.2018.00349 PMC6250822

[6] Committee on the Diagnostic Criteria for Myalgic Encephalomyelitis/Chronic Fatigue Syndrome; Board on the Health of Select Populations; Institute of Medicine. Beyond myalgic encephalomyelitis/chronic fatigue syndrome: redefining an illness. Washington (DC): National Academies Press (US); 2015. 10.17226/19012 25695122

[7] Carruthers BM, Jain AK, De Meirleir KL, Peterson DL, Klimas NG, Lerner AM, Bested AC, Flor-Henry P, Joshi P, Powles ACP, Sherkey JA, van de Sande MI (2003) Myalgic encephalomyelitis/chronic fatigue syndrome. J Chronic Fatigue Syndr 11(1):7–115. 10.1300/J092v11n01_02

[8] Stewart JM, Gewitz MH, Weldon A, Arlievsky N, Li K, Munoz J (1999) Orthostatic intolerance in adolescent chronic fatigue syndrome. Pediatrics 103(1):116–121. 10.1542/peds.103.1.116 9917448 10.1542/peds.103.1.116

[9] Guyatt G, Oxman AD, Akl EA, Kunz R, Vist G, Brozek J, Norris S, Falck-Ytter Y, Glasziou P, DeBeer H, Jaeschke R, Rind D, Meerpohl J, Dahm P, Schünemann HJ (2011) GRADE guidelines: 1. introduction-GRADE evidence profiles and summary of findings tables. J Clin Epidemiol 64(4):383–394. 10.1016/j.jclinepi.2010.04.026 21195583 10.1016/j.jclinepi.2010.04.026

[10] Gamboa A, Paranjape SY, Black BK, Arnold AC, Figueroa R, Okamoto LE, Nwazue VC, Diedrich A, Plummer WD, Dupont WD, Robertson D, Raj SR (2015) Inspiratory resistance improves postural tachycardia: a randomized study. Circ Arrhythm Electrophysiol 8(3):651–658. 10.1161/circep.114.002605 25792354 10.1161/CIRCEP.114.002605 PMC4472504

[11] Smith EC, Diedrich A, Raj SR, Gamboa A, Shibao CA, Black BK, Peltier A, Paranjape SY, Biaggioni I, Okamoto LE (2020) Splanchnic venous compression enhances the effects of β-blockade in the treatment of postural tachycardia syndrome. J Am Heart Assoc 9(14):e016196 32673517 10.1161/JAHA.120.016196 PMC7660715

[12] Bourne KM, Sheldon RS, Hall J, Lloyd M, Kogut K, Sheikh N, Jorge J, Ng J, Exner DV, Tyberg JV, Raj SR (2021) Compression garment reduces orthostatic tachycardia and symptoms in patients with postural orthostatic tachycardia syndrome. J Am Coll Cardiol 77(3):285–296. 10.1016/j.jacc.2020.11.040 33478652 10.1016/j.jacc.2020.11.040

[13] Nardone M, Guzman J, Harvey PJ, Floras JS, Edgell H (2020) Effect of a neck compression collar on cardiorespiratory and cerebrovascular function in postural orthostatic tachycardia syndrome (POTS). J Appl Physiol (1985) 128(4):907–913. 10.1152/japplphysiol.00040.2020 32163327 10.1152/japplphysiol.00040.2020

[14] Heyer GL (2014) Abdominal and lower-extremity compression decreases symptoms of postural tachycardia syndrome in youth during tilt table testing. J Pediatr 165(2):395–397. 10.1016/j.jpeds.2014.04.014 24840763 10.1016/j.jpeds.2014.04.014

[15] Wheatley-Guy CM, Shea MG, Parks JK, Scales R, Goodman BP, Butterfield RJ, Johnson BD (2023) Semi-supervised exercise training program more effective for individuals with postural orthostatic tachycardia syndrome in randomized controlled trial. Clin Auton Res 33(6):659–672. 10.1007/s10286-023-00970-w 37598401 10.1007/s10286-023-00970-w PMC10751269

[16] Gibbons CH, Silva G, Freeman R (2021) Cardiovascular exercise as a treatment of postural orthostatic tachycardia syndrome: a pragmatic treatment trial. Heart Rhythm 18(8):1361–1368. 10.1016/j.hrthm.2021.01.017 33482385 10.1016/j.hrthm.2021.01.017

[17] Shibata S, Fu Q, Bivens TB, Hastings JL, Wang W, Levine BD (2012) Short-term exercise training improves the cardiovascular response to exercise in the postural orthostatic tachycardia syndrome. J Physiol 590(15):3495–3505. 10.1113/jphysiol.2012.233858 22641777 10.1113/jphysiol.2012.233858 PMC3547265

[18] Fu Q, Vangundy TB, Galbreath MM, Shibata S, Jain M, Hastings JL, Bhella PS, Levine BD (2010) Cardiac origins of the postural orthostatic tachycardia syndrome. J Am Coll Cardiol 55(25):2858–2868. 10.1016/j.jacc.2010.02.043 20579544 10.1016/j.jacc.2010.02.043 PMC2914315

[19] George SA, Bivens TB, Howden EJ, Saleem Y, Galbreath MM, Hendrickson D, Fu Q, Levine BD (2016) The international POTS registry: evaluating the efficacy of an exercise training intervention in a community setting. Heart Rhythm 13(4):943–950. 10.1016/j.hrthm.2015.12.012 26690066 10.1016/j.hrthm.2015.12.012

[20] Svensson A, Svensson-Raskh A, Holmström L, Hallberg C, Bezuidenhout L, Moulaee Conradsson D, Ståhlberg M, Bruchfeld J, Fedorowski A, Nygren-Bonnier M (2024) Individually tailored exercise in patients with postural orthostatic tachycardia syndrome related to post-COVID-19 condition - a feasibility study. Sci Rep 14(1):20017. 10.1038/s41598-024-71055-5 39198662 10.1038/s41598-024-71055-5 PMC11358431

[21] Garland EM, Gamboa A, Nwazue VC, Celedonio JE, Paranjape SY, Black BK, Okamoto LE, Shibao CA, Biaggioni I, Robertson D, Diedrich A, Dupont WD, Raj SR (2021) Effect of high dietary sodium intake in patients with postural tachycardia syndrome. J Am Coll Cardiol 77(17):2174–2184. 10.1016/j.jacc.2021.03.005 33926653 10.1016/j.jacc.2021.03.005 PMC8103825

[22] Stavrakis S, Chakraborty P, Farhat K, Whyte S, Morris L, Abideen Asad ZU, Karfonta B, Anjum J, Matlock HG, Cai X, Yu X (2024) Noninvasive vagus nerve stimulation in postural tachycardia syndrome: a randomized clinical trial. JACC Clin Electrophysiol 10(2):346–355. 10.1016/j.jacep.2023.10.015 37999672 10.1016/j.jacep.2023.10.015 PMC10932945

[23] Z’Graggen WJ, Hess CW, Humm AM (2010) Acute fluid ingestion in the treatment of orthostatic intolerance - important implications for daily practice. Eur J Neurol 17(11):1370–1376. 10.1111/j.1468-1331.2010.03030.x 20412295 10.1111/j.1468-1331.2010.03030.x

[24] Rodriguez B, Zimmermann R, Gutbrod K, Heinemann D, Z’Graggen WJ (2019) Orthostatic cognitive dysfunction in postural tachycardia syndrome after rapid water drinking. Front Neurosci 13:327. 10.3389/fnins.2019.00327 31024242 10.3389/fnins.2019.00327 PMC6465605

[25] Rodriguez B, Hochstrasser A, Eugster PJ, Grouzmann E, Müri RM, Z’Graggen WJ (2022) Brain fog in neuropathic postural tachycardia syndrome may be associated with autonomic hyperarousal and improves after water drinking. Front Neurosci 16:968725. 10.3389/fnins.2022.968725 35992935 10.3389/fnins.2022.968725 PMC9388780

[26] Gordon VM, Opfer-Gehrking TL, Novak V, Low PA (2000) Hemodynamic and symptomatic effects of acute interventions on tilt in patients with postural tachycardia syndrome. Clin Auton Res 10(1):29–33. 10.1007/bf02291387 10750641 10.1007/BF02291387

[27] Jacob G, Shannon JR, Black B, Biaggioni I, Mosqueda-Garcia R, Robertson RM, Robertson D (1997) Effects of volume loading and pressor agents in idiopathic orthostatic tachycardia. Circulation 96(2):575–580. 10.1161/01.cir.96.2.575 9244228 10.1161/01.cir.96.2.575

[28] Ruzieh M, Baugh A, Dasa O, Parker RL, Perrault JT, Renno A, Karabin BL, Grubb B (2017) Effects of intermittent intravenous saline infusions in patients with medication-refractory postural tachycardia syndrome. J Interv Card Electrophysiol 48(3):255–260. 10.1007/s10840-017-0225-y 28185102 10.1007/s10840-017-0225-y

[29] Zha K, Brook J, McLaughlin A, Blitshteyn S (2023) Gluten-free diet in postural orthostatic tachycardia syndrome (POTS). Chronic Illn 19(2):409–417. 10.1177/17423953221076984 35098721 10.1177/17423953221076984

[30] Raj SR, Black BK, Biaggioni I, Paranjape SY, Ramirez M, Dupont WD, Robertson D (2009) Propranolol decreases tachycardia and improves symptoms in the postural tachycardia syndrome: less is more. Circulation 120(9):725–734. 10.1161/circulationaha.108.846501 19687359 10.1161/CIRCULATIONAHA.108.846501 PMC2758650

[31] Moon J, Kim DY, Lee WJ, Lee HS, Lim JA, Kim TJ, Jun JS, Park B, Byun JI, Sunwoo JS, Lee ST, Jung KH, Park KI, Jung KY, Kim M, Lee SK, Chu K (2018) Efficacy of propranolol, bisoprolol, and pyridostigmine for postural tachycardia syndrome: a randomized clinical trial. Neurotherapeutics 15(3):785–795. 10.1007/s13311-018-0612-9 29500811 10.1007/s13311-018-0612-9 PMC6095784

[32] Freitas J, Santos R, Azevedo E, Costa O, Carvalho M, de Freitas AF (2000) Reversible sympathetic vasomotor dysfunction in POTS patients. Rev Port Cardiol 19(11):1163–1170 11201632

[33] Zhao J, Du S, Yang J, Lin J, Tang C, Du J, Jin H (2014) Usefulness of plasma copeptin as a biomarker to predict the therapeutic effectiveness of metoprolol for postural tachycardia syndrome in children. Am J Cardiol 114(4):601–605. 10.1016/j.amjcard.2014.05.039 24996552 10.1016/j.amjcard.2014.05.039

[34] Chen L, Wang L, Sun J, Qin J, Tang C, Jin H, Du J (2011) Midodrine hydrochloride is effective in the treatment of children with postural orthostatic tachycardia syndrome. Circ J 75(4):927–931. 10.1253/circj.cj-10-0514 21301135 10.1253/circj.cj-10-0514

[35] Raj SR, Black BK, Biaggioni I, Harris PA, Robertson D (2005) Acetylcholinesterase inhibition improves tachycardia in postural tachycardia syndrome. Circulation 111(21):2734–2740. 10.1161/circulationaha.104.497594 15911704 10.1161/CIRCULATIONAHA.104.497594

[36] Stewart JM, Warsy IA, Visintainer P, Terilli C, Medow MS (2021) Supine parasympathetic withdrawal and upright sympathetic activation underly abnormalities of the baroreflex in postural tachycardia syndrome: effects of pyridostigmine and digoxin. Hypertension 77(4):1234–1244. 10.1161/hypertensionaha.120.16113 33423527 10.1161/HYPERTENSIONAHA.120.16113 PMC7946724

[37] Kanjwal K, Karabin B, Sheikh M, Elmer L, Kanjwal Y, Saeed B, Grubb BP (2011) Pyridostigmine in the treatment of postural orthostatic tachycardia: a single-center experience. Pacing Clin Electrophysiol 34(6):750–755. 10.1111/j.1540-8159.2011.03047.x 21410722 10.1111/j.1540-8159.2011.03047.x

[38] Coffin ST, Black BK, Biaggioni I, Paranjape SY, Orozco C, Black PW, Dupont WD, Robertson D, Raj SR (2012) Desmopressin acutely decreases tachycardia and improves symptoms in the postural tachycardia syndrome. Heart Rhythm 9(9):1484–1490. 10.1016/j.hrthm.2012.05.002 22561596 10.1016/j.hrthm.2012.05.002 PMC3419341

[39] Taub PR, Zadourian A, Lo HC, Ormiston CK, Golshan S, Hsu JC (2021) Randomized trial of ivabradine in patients with hyperadrenergic postural orthostatic tachycardia syndrome. J Am Coll Cardiol 77(7):861–871. 10.1016/j.jacc.2020.12.029 33602468 10.1016/j.jacc.2020.12.029

[40] Barzilai M, Jacob G (2015) The effect of ivabradine on the heart rate and sympathovagal balance in postural tachycardia syndrome patients. Rambam Maimonides Med J. 10.5041/rmmj.10213 26241226 10.5041/RMMJ.10213 PMC4524401

[41] Ruzieh M, Sirianni N, Ammari Z, Dasa O, Alhazmi L, Karabin B, Grubb B (2017) Ivabradine in the treatment of postural tachycardia syndrome (POTS), a single center experience. Pacing Clin Electrophysiol 40(11):1242–1245. 10.1111/pace.13182 28846151 10.1111/pace.13182

[42] Towheed A, Nesheiwat Z, Mangi MA, Karabin B, Grubb BP (2020) Ivabradine in children with postural orthostatic tachycardia syndrome: a retrospective study. Cardiol Young 30(7):975–979. 10.1017/s1047951120001341 32498748 10.1017/S1047951120001341

[43] Ross AJ, Ocon AJ, Medow MS, Stewart JM (2014) A double-blind placebo-controlled cross-over study of the vascular effects of midodrine in neuropathic compared with hyperadrenergic postural tachycardia syndrome. Clin Sci (Lond) 126(4):289–296. 10.1042/cs20130222 23978222 10.1042/CS20130222 PMC3896075

[44] Arnold AC, Ng J, Raj SR (2018) Postural tachycardia syndrome – diagnosis, physiology, and prognosis. Autonom Neurosci 215:3–11. 10.1016/j.autneu.2018.02.005 10.1016/j.autneu.2018.02.005 PMC6113123 29523389

[45] Hoeldtke RD, Bryner KD, Hoeldtke ME, Hobbs G (2006) Treatment of postural tachycardia syndrome: a comparison of octreotide and midodrine. Clin Auton Res 16(6):390–395. 10.1007/s10286-006-0373-0 17036177 10.1007/s10286-006-0373-0

[46] Zhang F, Li X, Ochs T, Chen L, Liao Y, Tang C, Jin H, Du J (2012) Midregional pro-adrenomedullin as a predictor for therapeutic response to midodrine hydrochloride in children with postural orthostatic tachycardia syndrome. J Am Coll Cardiol 60(4):315–320. 10.1016/j.jacc.2012.04.025 22813609 10.1016/j.jacc.2012.04.025

[47] Yang J, Zhao J, Du S, Liu D, Fu C, Li X, Chen S, Tang C, Du J, Jin H (2013) Postural orthostatic tachycardia syndrome with increased erythrocytic hydrogen sulfide and response to midodrine hydrochloride. J Pediatr 163(4):1169-1173.e1162. 10.1016/j.jpeds.2013.04.039 23726544 10.1016/j.jpeds.2013.04.039

[48] Kpaeyeh J Jr., Mar PL, Raj V, Black BK, Arnold AC, Biaggioni I, Shibao CA, Paranjape SY, Dupont WD, Robertson D, Raj SR (2014) Hemodynamic profiles and tolerability of modafinil in the treatment of postural tachycardia syndrome: a randomized, placebo-controlled trial. J Clin Psychopharmacol 34(6):738–741. 10.1097/jcp.0000000000000221 25222185 10.1097/JCP.0000000000000221 PMC4239166

[49] Vernino S, Hopkins S, Bryarly M, Hernandez RS, Salter A (2024) Randomized controlled trial of intravenous immunoglobulin for autoimmune postural orthostatic tachycardia syndrome (iSTAND). Clin Auton Res 34(1):153–163. 10.1007/s10286-024-01020-9 38311655 10.1007/s10286-024-01020-9

[50] Green EA, Raj V, Shibao CA, Biaggioni I, Black BK, Dupont WD, Robertson D, Raj SR (2013) Effects of norepinephrine reuptake inhibition on postural tachycardia syndrome. J Am Heart Assoc 2(5):e000395. 10.1161/jaha.113.000395 24002370 10.1161/JAHA.113.000395 PMC3835251

[51] Green EA, Black BK, Biaggioni I, Paranjape SY, Bagai K, Shibao C, Okoye MC, Dupont WD, Robertson D, Raj SR (2014) Melatonin reduces tachycardia in postural tachycardia syndrome: a randomized, crossover trial. Cardiovasc Ther 32(3):105–112. 10.1111/1755-5922.12067 24495468 10.1111/1755-5922.12067 PMC3999238

[52] Mar PL, Raj V, Black BK, Biaggioni I, Shibao CA, Paranjape SY, Dupont WD, Robertson D, Raj SR (2014) Acute hemodynamic effects of a selective serotonin reuptake inhibitor in postural tachycardia syndrome: a randomized, crossover trial. J Psychopharmacol 28(2):155–161. 10.1177/0269881113512911 24227635 10.1177/0269881113512911 PMC3956655

[53] Vyas R, Nesheiwat Z, Ruzieh M, Ammari Z, Al-Sarie M, Grubb B (2020) Bupropion in the treatment of postural orthostatic tachycardia syndrome (POTS): a single-center experience. J Investig Med 68(6):1156–1158. 10.1136/jim-2020-001272 32606041 10.1136/jim-2020-001272

[54] Ruzieh M, Dasa O, Pacenta A, Karabin B, Grubb B (2017) Droxidopa in the treatment of postural orthostatic tachycardia syndrome. Am J Ther 24(2):e157–e161. 10.1097/mjt.0000000000000468 27563801 10.1097/MJT.0000000000000468

[55] Kanjwal K, Saeed B, Karabin B, Kanjwal Y, Sheikh M, Grubb BP (2012) Erythropoietin in the treatment of postural orthostatic tachycardia syndrome. Am J Ther 19(2):92–95. 10.1097/MJT.0b013e3181ef621a 20838326 10.1097/MJT.0b013e3181ef621a

[56] Freeman R, Wieling W, Axelrod FB, Benditt DG, Benarroch E, Biaggioni I, Cheshire WP, Chelimsky T, Cortelli P, Gibbons CH, Goldstein DS, Hainsworth R, Hilz MJ, Jacob G, Kaufmann H, Jordan J, Lipsitz LA, Levine BD, Low PA, Mathias C, Raj SR, Robertson D, Sandroni P, Schatz I, Schondorff R, Stewart JM, van Dijk JG (2011) Consensus statement on the definition of orthostatic hypotension, neurally mediated syncope and the postural tachycardia syndrome. Clin Auton Res 21(2):69–72. 10.1007/s10286-011-0119-5 21431947 10.1007/s10286-011-0119-5

[57] Grubb BP, Kosinski DJ, Boehm K, Kip K (1997) The postural orthostatic tachycardia syndrome: a neurocardiogenic variant identified during head-up tilt table testing. Pacing Clin Electrophysiol 20(9 Pt 1):2205–2212. 10.1111/j.1540-8159.1997.tb04238.x 9309745 10.1111/j.1540-8159.1997.tb04238.x

[58] Keicher F, Thomann J, Erlenwein J, Schottdorf M, Reiter NL, Scholz-Schwärzler NP, Vogel B, Warlitz C, Stojanov S, Augustin S, Goldbrunner L, Schanz L, Dodel V, Zipper C, Schiweck N, Jaeschke R, Saramandic M, Wiejaczka K, Eberhartinger M, Dettmer K, Hattesohl DBR, Englbrecht S, Behrends U, Spiegler J (2024) Development and implementation of an online patient education program for children and adolescents with myalgic encephalomyelitis/chronic fatigue syndrome, their parents, siblings, and school personnel: protocol for the Prospective BAYNET FOR ME/CFS Study. JMIR Res Protoc 13:e54679 39570662 10.2196/54679 PMC11621712

