# Supplementary material for: Systematic literature review: treatment of postural orthostatic tachycardia syndrome (POTS)
Source: Clin Auton Res. 2025 Nov 12;36(1):3–16. doi: 10.1007/s10286-025-01172-2 (PMC12982215; doi:10.1007/s10286-025-01172-2)
Supplement: Supplementary file 1 — Supplementary file1 (DOCX 249 kb) [file 10286_2025_1172_MOESM1_ESM.docx]

**Supplementary Material**

**Supplement 1 – Literature Research Strategy**

**Systematic Literature Review - Treatment of Postural orthostatic Tachycardia Syndrome (PoTS) in children and adults**

**SEARCH STRATEGY**

**DUE TO THE LIMITED NUMBER OF STUDIES, GENERAL LITERATURE SEARCH WILL BE PERFORMED BEFORE APPLYING PICO CRITERIA, USING THE FOLLOWING SEARCH STRATEGY:**

1. **) TERMS AND SYNONYMS**

**=> CAVE! WE DID NOT INCLUDE THE TERM “ORTHOSTATIC INTOLERANCE” SINCE THIS INCLUDES A LARGE NUMBER OF ARTICLES AND DISEASES NOT DIRECTLY RELATED TO THE RESEARCH QUESTION**

**USING THE PUBMED ADVANCED RESEARCH BUILDER TO FIND SYNONYMS:**

**
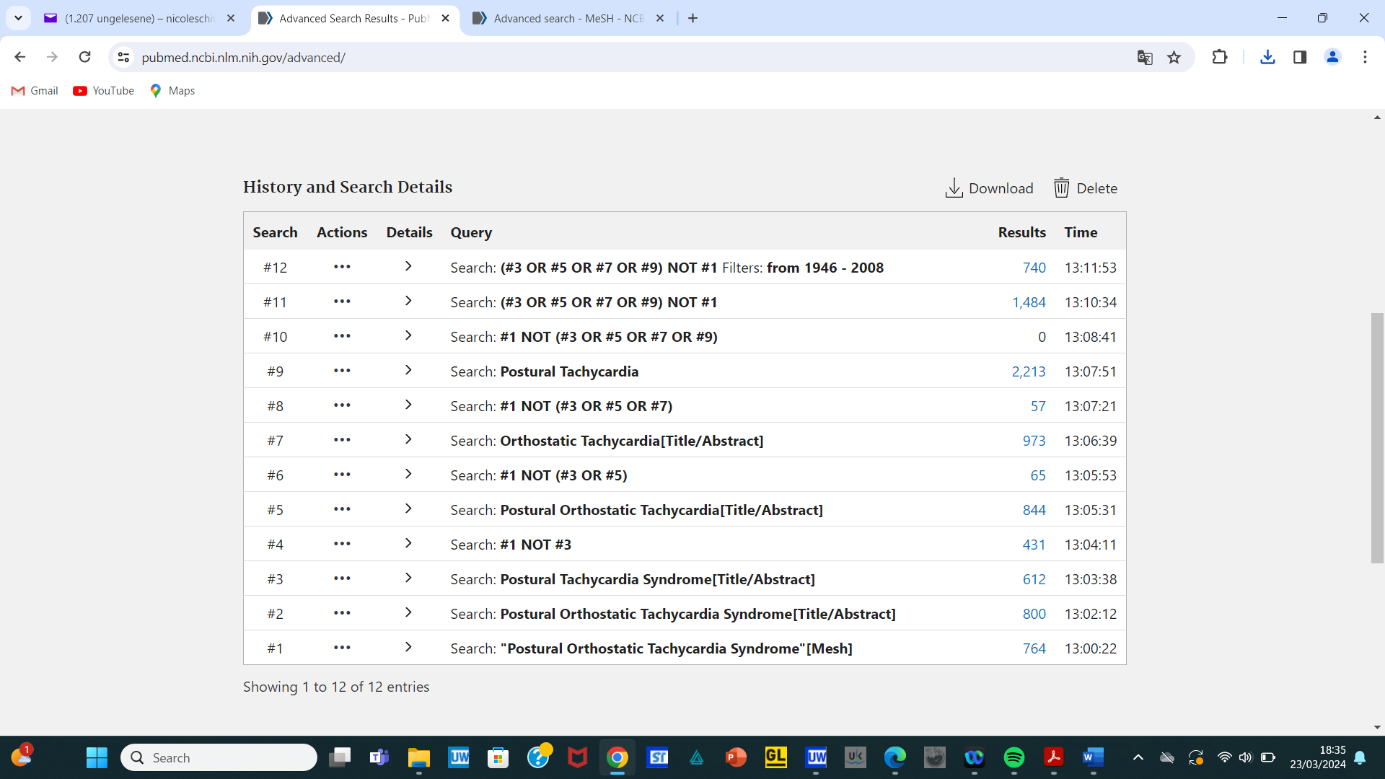
**

- **“Postural orthostatic Tachycardia Syndrome” [MeSH]** includes
  - Postural Tachycardia Syndrome
  - Syndrome, Postural Tachycardia
  - Tachycardia Syndrome, Postural
- TIME FILTER APPLIED (Publications before 2009): The MeSH term for PoTS was registered in 2009. In order to identify earlier publications describing the condition without the MeSH term being applied afterwards, an additional time filter was set.

| SYNONYMS IDENTIFIED / TERMS USED |
| --- |
| “Postural orthostatic Tachycardia Syndrome” [MeSH] |
| “Postural Tachycardia Syndrome” |
| “Postural Orthostatic Tachycardia” |
| “Orthostatic Tachycardia” |
| “Postural Tachcardia” |

1. SEARCH STRATEGY APPLYING ALL SYNONYMS IDENTIFIED

**PUBMED / MEDLINE:**

"Postural Orthostatic Tachycardia Syndrome"[Mesh] OR “Postural Tachycardia Syndrome” OR “Postural Orthostatic Tachycardia” OR “Orthostatic Tachycardia” OR “Postural Tachycardia”

**COCHRANE LIBRARIES**

- MeSH descriptor: [Postural Orthostatic Tachycardia Syndrome] explode all trees
- (postural orthostatic tachycardia syndrome) OR (Postural Tachycardia Syndrome) OR (Postural Orthostatic Tachycardia) OR (Orthostatic Tachycardia) OR (Postural Tachycardia)

**ClinicalTrials.gov**

- "Postural Orthostatic Tachycardia Syndrome" OR “Postural Tachycardia Syndrome” OR “Postural Orthostatic Tachycardia” OR “Orthostatic Tachycardia” OR “Postural Tachycardia”

**OVID**

- "Postural Orthostatic Tachycardia Syndrome" OR “Postural Tachycardia Syndrome” OR “Postural Orthostatic Tachycardia” OR “Orthostatic Tachycardia” OR “Postural Tachycardia”
- Filter: Human
- Databases included:
  - GeoREF (GREF)
  - ZOOR Zoological Record (ZOOR)
  - Ovid Nutrition and Health (FSMN)
  - Food Sciences and Technological Abstracts (FSTA)
  - AGRICOLA (AGRA)
  - JBI EBP
  - MEDLINE
  - Maternity and Infant Care (MWIC)
  - Embase : Excerpta Medica (EMED)
  - Embase Classic (EMCL)
  - Petroleum Abstracts TULSA (PTLM)
  - APA PsycBooks (PSBK)
  - APA PsycTests (PSYT)
  - APA PsycTherapy (PSTH)
  - APA PsycExtra (PSYE)
  - International Index to TV Periodicals (IITP-OV)
  - International Index to Film Periodicals (IIFP-OV)
  - PSYNDEXplus Literature and Audiovisual Media (PSYN)
  - PSYNDEXplus Tests (PSKM)
  - APA PsycInfo (PSYC)
  - Transplant Library (TRLI)
  - Psychoanalytic Electronic Publishing Archive (PEPA)
  - APA PsycTherapy (PSTH)
  - Northern Light Sciences Conference Abstracts (DSCV)
  - Inspec (INSP)

Duplicates removed.

**PICO(S)**

- **PARTICIPANTS / POPULATION:**

PEOPLE DIAGNOSED WITH / SUFFERING FROM POTS (ALL AGE GROUPS)

- **INTERVENTION:**

ANY KIND OF INTERVENTION, s.a.

- - NON-PHARMACOLOGICAL APPROACHES (s.a. physiotherapy, manual therapy, patient education,...)
  - PHARMACOLOGICAL APPROACHES
- **COMPARATOR / CONTROL**
  - PATIENTS DIAGNOSED WITH POTS UNDERGOING ROUTINE CARE / PLACEBO
  - NO TREATMENT
  - PRE- AND POST-TREATMENT STUDY RESULTS
- **OUTCOME**

| PRIMARY OUTCOME | MEASUREMENT |
| --- | --- |
| DECLINE IN HEART RATE INCREASE UPON POSTURAL CHANGE (E.G. TILT-TEST; SCHELLONG-TEST) | HEART RATE INCREASE < 30 bpm (ADULT)  or < 40 bpm (CHILD) OR HEART RATE LESS THAN 120 bpm (130 bpm) IN TOTAL  REGULAR HEART FREQUENCY (WITHOUT WORK LOAD) AT NORMAL LEVELS (60 – 80 bpm (ADULTS), AGE-ADAPTED HEART FREQUENCY (CHILDREN))  REDUCTION OF ARRHYTHMIA |
| NORMAL RANGE OF SYSTOLIC / DIASTOLIC BLOOD PRESSURE (BP) UPON POSTURAL CHANGE | SCHELLONG-TEST / TILT-TEST / NASA – 10- MINUTES-LEAN-TEST (OR SIMILAR TESTS): DECREASE OF SYSTOLIC BP < 20 mmHg and INCREASE OF DIASTOLIC BP NO MORE THAN 10 mmHg  REGULAR AND / OR CONTINUOUS BLOOD PRESSURE MEASUREMENT: BP AT NORMAL LEVELS (< 140 / 90 mmHg; NO SYMPTOMATIC HYPOTENSION) |
| DYSFUNCTIONAL BREATHING | LUNG FUNCTION TESTS, BLOOD GAS ANALYSIS, SYMPTOM-BURDEN-QUESTIONNAIRE |
| REGULATION OF THE AUTONOMOUS NERVOUS SYSTEM AND HEMODYNAMIC SYSTEM | QUESTIONNAIRES, NUMBER OF EPISODES OF AUTONOMOUS SYMPTOMS SUCH AS DIARRHEA, FLUSH, SWEATING, …  (LABORATORY TESTS: PLASMA CATECHOLAMINE LEVELS, ANP-LEVELS, BARORECEPTOR ACTIVITY, TOTAL PERIPHERY RESISTANCE, RENIN-ANGIOTENSIN-ALDOSTERON-SYSTEM ACTIVITY) |
| FALLS AND / OR SYNCOPIES | NUMBER OF FALLS / SYNCOPIES |
| NAUSEA  DIZZINESS | QUESTIONNAIRE, NEUROLOGICAL EXAMINATION |
| REMISSION | ASSESSING REMISSION OF PoTS, APPLYING DIAGNOSTIC CRITERIA |

| ADDITIONAL OUTCOME | MEASUREMENT |
| --- | --- |
| ATTENTIVENESS /  COGNITIVE FUNCTION | COGNITIVE FUNCTION TESTS, SUCH AS MINI-MENTAL STATE EXAMINATION, MONTREAL COGNITIVE ASSESSMENT OR SIMILAR TESTS |
| REDUCTION OF FATIGUE | QUESTIONNAIRES OR SCALES, SUCH AS THE FATIGUE ASSESSMENT SCALE, BELL SCORE |
| MENTAL HEALTH /  REDUCTION OF DEPRESSION AND / OR  ANXIETY | QUESTIONNAIRES OR SCALES, SUCH AS THE HAMILTON DEPRESSION RATING SCALE , MONTGOMERY-ASBERG RATING SCALE, CHILDREN´S DEPRESSION INVENTORY, CHILDREN´S DEPRESSION RATING SCALE, HAMILTON ANXIETY RATING SCALE OR SIMILAR SCALES |
| PARTICIPATION IN DAILY ACTIVITIES | QUESTIONNAIRE |
| QUALITY OF LIFE /  WELL-BEING | SCALES AND QUESTIONNAIRES, SUCH AS EQ-5D, SF36 |

**STUDY DESIGN**

- - Randomized-Controlled Trials
  - Non-randomized Controlled Trials
  - Prospective cohort studies
  - Case-control-studies
  - Case series

**ELECTRONIC DATABASES SEARCHED:**

- PubMed/MEDLINE
- Cochrane Library
- Ovid (for included databases, please see above)
- Clinicaltrials.gov

**SUBGROUPS**

- Myalgic Encephalomyelitis / Chronic Fatigue Syndrome
- Long-Covid-19
- Children, Adolescents or alternatively Mean Age

**ARTICLE TYPES**

- Randomized-Controlled Trials (RCT)
- Non-randomized controlled-trials
- Prospective cohort studies
- Case-control studies
- Case series

**LANGUAGE RESTRICTIONS:**

- English
- German

LITERATURE SEARCH WAS PERFORMED ON 15^th^ May 2024.

ARTICLES WERE NOT EXCLUDED DUE TO THEIR PUBLICATION DATE (NO TIME FRAME FOR INCLUDED LITERATURE)

GREY LITERATURE WAS EXCLUDED.
