# Supplementary material for: Systematic literature review: treatment of postural orthostatic tachycardia syndrome (POTS)
Source: Clin Auton Res. 2025 Nov 12;36(1):3–16. doi: 10.1007/s10286-025-01172-2 (PMC12982215; doi:10.1007/s10286-025-01172-2)
Supplement: Supplementary file 2 — Supplementary file2 (DOCX 28 kb) [file 10286_2025_1172_MOESM2_ESM.docx]

| Registration Code | Title according to ClinicalTrials.gov  **List of registered Clinical Trials (Results not available / not found)** |
| --- | --- |
| NCT05918978 | **Open Label Extension of Efgartigimod in Adults With Post-COVID-19 POTS** |
| NCT04943276 | **A Novel Noninvasive Thermoregulatory Device for Postural Tachycardia Syndrome** |
| NCT05914649 | **NC Testing in LC & POTS** |
| NCT04632134 | **Long-term Effects of Transcutaneous Vagal Nerve Stimulation on Postural Orthostatic Tachycardia Syndrome (POTS)** |
| NCT05094622 | **Physical Training in Patients With POTS After Covid-19** |
| NCT04875949 | **Anti-Cholinergic Receptors Antibodies, Autonomic Profile and Dysautonomia Symptoms in PAF, ALS and POTS (DISAUT-AB)** |
| NCT00581633 | **Acute Salt Handling in Orthostatic Intolerance** |
| NCT05421208 | **Cardiovascular Autonomic and Immune Mechanism of Post COVID-19 Tachycardia Syndrome** |
| NCT03185247 | **Implementation of Transdx Group for POTS** |
| ISRCTN45323485 | **Assessing the feasibility of a supervised exercise rehabilitation intervention with behavioural and motivational support, for people with postural orthostatic tachycardia syndrome** |
| NCT00409435 | **A Study of Pyridostigmine in Postural Tachycardia Syndrome** |
| NCT01000350 | **Intravenous (IV) Saline and Exercise in Postural Tachycardia Syndrome (POTS)** |
| NCT01988883 | **Modafinil and Cognitive Function in POTS** |
| NCT02281097 | **Transdermal Vagal Stimulation for POTS** |
| NCT02558972 | **Northera Improves Postural Tachycardia Syndrome (POTS) and Postural Vasovagal Syncope (VVS)** |
| NCT02854683 | **Reducing Orthostatic Intolerance With Oral Rehydration in Myalgic Encephalomyelitis/Chronic Fatigue Syndrome Patients** |
| NCT03124355 | **Vagal Stimulation in POTS** |
| NCT04140721 | **Autonomic Determinants of POTS - Pilot 2** |
| NCT04186286 | **Crossover Study of Propranolol vs Ivabradine in POTS** |
| NCT04345432 | **Gabapentin Treatment of Postural Tachycardia Syndrome (PoTS)** |
| NCT04881318 | **Compression Garments in the Community With POTS** |
| NCT05363514 | **Low Dose Naltrexone Use in Patients With POTS** |
| NCT05404672 | **Breathing Exercises With And Without Aerobic Training In Patients With Postural Orthostatic Tachycardia Syndrome** |
| NCT05454137 | **A Shared Medical Appointment Intervention for Quality of Life Improvement in POTS** |
| NCT05481177 | **Ivabradine for Long-Term Effects of COVID-19 With POTS Cohort** |
| NCT05554107 | **The Effect of Physical Activity on Postural Orthostatic Tachycardia Syndrome** |
| NCT05633407 | **Efficacy and Safety Study of Efgartigimod in Adults With Post-COVID-19 POTS** |
| NCT05823896 | **imPROving Quality of LIFe In the Long COVID Patient** |
| NCT05877534 | **Effects of Individual Tailored Physical Exercise in Patients With POTS After COVID-19 - a Randomized Controlled Study** |
| NCT05924646 | **CAlgary SAlt for POTS** |
| NCT06133075 | **Using Mirabegron to Increase BP in Patients With POTS** |
| NCT06268288 | **Non-invasive Vagal Neurostimulation (nVNS) in Adolescents With Postural Orthostatic Tachycardia Syndrome (POTS)** |
