# Supplementary material for: Systematic literature review: treatment of postural orthostatic tachycardia syndrome (POTS)
Source: Clin Auton Res. 2025 Nov 12;36(1):3–16. doi: 10.1007/s10286-025-01172-2 (PMC12982215; doi:10.1007/s10286-025-01172-2)
Supplement: Supplementary file 3 — Supplementary file3 (DOCX 37 kb) [file 10286_2025_1172_MOESM3_ESM.docx]

| **Supplement 3 - Included studies (Overview)** | | | | | | | | | |
| --- | --- | --- | --- | --- | --- | --- | --- | --- | --- |
| **Study ID** | **Title** | **Authors** | **Country** | **Year of Publication** | **Non-RCT vs. RCT** | **Medication** | **Non-pharmacological_intervention** | **Total number of participants at baseline** | **Funding Sources** |
| Barzilai 2015 | The Effect of Ivabradine on the Heart Rate and Sympathovagal Balance in Postural Tachycardia Syndrome Patients | Merav Barzilai, M.D., and Giris Jacob, M.D., D.Sc. | Israel | 2015 | Non-RCT study | Ivabradine | There is no non-pharmacological intervention in this study. | 8 | not given |
| Bourne 2021 | Compression Garment Reduces Orthostatic Tachycardia and Symptoms in Patients With Postural Orthostatic Tachycardia Syndrome | Kate M. Bourne, BSC, Robert S. Sheldon, MD, PHD, Juliette Hall, Matthew Lloyd, PHD, Karolina Kogut, BSC, Nasia Sheikh, BSC, Juliana Jorge, MD, Jessica Ng, MSC, Derek V. Exner, MD, MPH, John V. Tyberg, MD, PHD, Satish R. Raj, MD, MSC | Canada | 2021 | RCT study | There is no medication in this study. | Compression Garments | 32 | non-commercial [Libin Cardiovascular Institute] |
| Chen 2011 | Midodrine hydrochloride is effective in the treatment of children with postural orthostatic tachycardia syndrome | Li Chen, MD; Li Wang, MD; Jinghui Sun, MD; Jiong Qin, MD; Chaoshu Tang, PhD; Hongfang Jin, PhD; Junbao Du, MD, PhD | China | 2011 | Non-RCT study | Beta-Adrenergic-Blocking-Agents; Midodrine | There is no non-pharmacological intervention in this study. | 53 | not given |
| Coffin 2012 | Desmopressin acutely decreases tachycardia and improves symptoms in the postural tachycardia syndrome | Samuel T. Coffin, MD, Bonnie K. Black, RN, CNP, Italo Biaggioni, MD, Sachin Y. Paranjape, BS, Carlos Orozco, BS, Phillip W. Black, BS, William D. Dupont, PhD, David Robertson, MD, Satish R. Raj, MD, MSCI, FHRS | United States of America | 2012 | RCT study | Desmopressin | There is no non-pharmacological intervention in this study. | 30 | non-commercial [National Institutes of Health grants; Paden Dysautonomia Center] |
| Freitas 2000 | Reversible sympathetic vasomotor dysfunction in POTS patients | João Freitas, M.D., Rosa Santos, B.Sc., Elsa Azevedo, M.D., Ovidio Costa, Ph.D., Mádrio Carvalho, M.D., and A. Falcão de Freitas, M.D., Ph.D., F.E.S.C. | Portugal | 2000 | Non-RCT study | Beta-Adrenergic-Blocking-Agents; Fludrocortisone | There is no non-pharmacological intervention in this study. | 11 | not given |
| Fu 2010 | Cardiac origins of the postural orthostatic tachycardia syndrome | Qi Fu, MD, PHD, Tiffany B. VanGundy, MS, M. Melyn Galbreath, PHD, Shigeki Shibata, MD, PHD, Manish Jain, MD, Jeffrey L. Hastings, MD, Paul S. Bhella, MD, Benjamin D. Levine, MD | United States of America | 2010 | Non-RCT study | There is no medication in this study. | Physical Exercise / Training | 19 | non-commercial [National Institutes of Health;National Space Biomedical Research Institute; Clinical and Translational Research Center] |
| Gamboa 2015 | Inspiratory resistance improves postural tachycardia: a randomized study | Alfredo Gamboa, MD, MSCI; Sachin Y. Paranjape, BS; Bonnie K. Black, RN, CNP; Amy C. Arnold, PhD, MSCI; Rocío Figueroa, MD; Luis E. Okamoto, MD; Victor C. Nwazue, MD, MSCI; Andre Diedrich, MD, PhD; W. Dale Plummer, BS; William D. Dupont, PhD; David Robertson, MD; Satish R. Raj, MD, MSCI | Canada; United States of America | 2015 | RCT study | There is no medication in this study. | Breathing Device; Other: Impedance treshold device (ITD) | 39 | not given |
| Garland 2021 | Effect of High Dietary Sodium Intake in Patients With Postural Tachycardia Syndrome | Emily M. Garland, PHD, MSCI, Alfredo Gamboa, MD, MSCI, Victor C. Nwazue, MD, MSCI, Jorge E. Celedonio, MD, Sachin Y. Paranjape, BS, Bonnie K. Black, RN, NP, Luis E. Okamoto, MD, Cyndya A. Shibao, MD, MSCI, Italo Biaggioni, MD, David Robertson, MD, André Diedrich, MD, PHD, William D. Dupont, PHD, Satish R. Raj, MD, MSCI | Canada; United States of America | 2021 | RCT study | There is no medication in this study. | Salt supplementation | 27 | non-commercial [National Heart, Lung, and Blood Institute of the National Institutes of Health; National Center for Advancing Translational Sciences; Vanderbilt Hormone & Analytical Services Core] |
| George 2016 | The international POTS registry: Evaluating the efficacy of an exercise training intervention in a community setting | Stephen A. George, MD, PhD, Tiffany B. Bivens, MS, Erin J. Howden, PhD, Yasir Saleem, MD, M. Melyn Galbreath, PhD, Dianne Hendrickson, MS, APRN, FNP-C, Qi Fu, MD, PhD, Benjamin D. Levine, MD | United States of America | 2016 | Non-RCT study | There is no medication in this study. | Physical Exercise / Training | 251 | not given |
| Gibbons 2021 | Cardiovascular exercise as a treatment of postural orthostatic tachycardia syndrome: A pragmatic treatment trial | Christopher H. Gibbons, MD, MMSc, Gustavo Silva, MD, Roy Freeman, MD | United States of America | 2021 | Non-RCT study | There is no medication in this study. | Physical Exercise / Training | 77 | No funding sources to disclose. |
| Gordon 2000 | Hemodynamic and symptomatic effects of acute interventions on tilt in patients with postural tachycardia syndrome | Victor M. Gordon, U.D., Tonette L. Opfer-Gehrking, Vera Novak, M.D., Ph.D., and Phillip A. Low, M.D. | United States of America | 1999 | Non-RCT study | Beta-Adrenergic-Blocking-Agents; Midodrine; Other: Clonidine, Phenobarbital | Salt supplementation; (Re-)Hydration | 21 | non-commercial [National Institute of Neurological Disorders and Stroke; National Aeronautics and Space Administration; Mayo funds] |
| Green 2013 | Effects of norepinephrine reuptake inhibition on postural tachycardia syndrome | Elizabeth A. Green, BEng; Vidya Raj, MB, ChB; Cyndya A. Shibao, MD, MSCI; Italo Biaggioni, MD; Bonnie K. Black, RN, CNP; William D. Dupont, PhD; David Robertson, MD; Satish R. Raj, MD, MSCI | United States of America | 2013 | RCT study | Other: Atomoxetine | There is no non-pharmacological intervention in this study. | 27 | non-commercial [NIH grants; Clinical and Translational Science Award] |
| Green 2014 | Melatonin reduces tachycardia in postural tachycardia syndrome: a randomized, crossover trial | Elizabeth A. Green, Bonnie K. Black, Italo Biaggioni, Sachin Y. Paranjape, Kanika Bagai, Cyndya Shibao, Mirian C. Okoye, William D. Dupont, David Robertson & Satish R. Raj | United States of America | 2014 | RCT study | Other: Melatonin | There is no non-pharmacological intervention in this study. | 78 | non-commercial [National Institutes of Health grants; Clinical and Translational Science Award] |
| Heyer 2014 | Abdominal and lower-extremity compression decreases symptoms of postural tachycardia syndrome in youth during tilt table testing | Geoffrey L. Heyer, MD | United States of America | 2014 | Non-RCT study | There is no medication in this study. | Compression Garments | 20 | not given |
| Hoeldtke 2006 | Treatment of postural tachycardia syndrome: a comparison of octreotide and midodrine | Robert D. Hoeldtke, Kimberly D. Bryner, Martin E. Hoeldtke, Gerald Hobbs | United States of America | 2006 | Non-RCT study | Midodrine; Octreotide | There is no non-pharmacological intervention in this study. | 10 | not given |
| Jacob 1997 | Effects of volume loading and pressor agents in idiopathic orthostatic tachycardia | Giris Jacob, John R. Shannon, Bonnie Black, Italo Biaggioni, Rogelio Mosqueda-Garcia, Rose Marie Robertson, and David Robertson | United States of America | 1997 | Non-RCT study | Midodrine; Other: Clonidine | Salt supplementation; (Re-)Hydration | 13 | non-commercial [National Institutes of Health grants; National Aeronautics and Space Administration grants] |
| Kanjwal 2011 | Pyridostigmine in the treatment of postural orthostatic tachycardia: a single-center experience | KHALIL KANJWAL, M.D., BEVERLY KARABIN, PH.D., MUJEEB SHEIKH, M.D., LAWRENCE ELMER, M.D., PH.D., YOUSUF KANJWAL, M.D., BILAL SAEED, M.D., and BLAIR P. GRUBB, M.D. | United States of America | 2011 | Non-RCT study | Pyridostigmine | There is no non-pharmacological intervention in this study. | 208 | not given |
| Kanjwal 2012 | Erythropoietin in the treatment of postural orthostatic tachycardia syndrome | Khalil Kaejwal, MD, Bilal Saeed, MD, Beverly Karabin, PhD, Yousuf Kanjwal, MD, Mujeeb Sheikh, MD, and Blair P. Grubb, MD | United States of America | 2012 | Non-RCT study | Other: Erythropoeitin | There is no non-pharmacological intervention in this study. | 39 | not given |
| Kanjwal 2018 | Role of implantable loop recorders in patients with postural orthostatic tachycardia syndrome | Khalil Kanjwal MD, Rehana Qadir MD, Mohammed Ruzieh MD, Blair P. Grubb MD | United States of America | 2018 | Non-RCT study | There is no medication in this study. | Other: dual-chamber pacemaker | 40 | not given |
| Kanjwal 2021 | Further Observations on the Use of Pacemakers in Patients with Postural Orthostatic Tachycardia Syndrome with Demonstrated Asystole | KHALIL KANJWAL, md, fhrs, facc, ccds, ceps(p,ASIM KICHLOO, md, REHANA QADIR, md, and BLAIR P. GRUBB, md | United States of America | 2021 | Non-RCT study | There is no medication in this study. | Other: dual-chamber pacemaker | 40 | not given |
| Kpaeyeh 2014 | Hemodynamic profiles and tolerability of modafinil in the treatment of postural tachycardia syndrome: a randomized, placebo-controlled trial | John Kpaeyeh Jr, MD, Philip L. Mar, MD, PharmD, Vidya Raj, MB, ChB, Bonnie K. Black, RN, CNP, Amy C. Arnold, PhD, Italo Biaggioni, MD, Cyndya A. Shibao, MD, MSCI, Sachin Y. Paranjape, BS, William D. Dupont, PhD, David Robertson, MD, and Satish R. Raj, MD, MSCI, FACC | Canada; United States of America | 2014 | RCT study | Modafinil | There is no non-pharmacological intervention in this study. | 54 | non-commercial [National Institutes of Health grants; Clinical and Translational] Science Award |
| Mar 2014 | Acute hemodynamic effects of a selective serotonin reuptake inhibitor in postural tachycardia syndrome: a randomized, crossover trial | Philip L Mar, Vidya Raj, Bonnie K Black, Italo Biaggioni, Cyndya A Shibao, Sachin Y Paranjape, William D Dupont, David Robertson and Satish R Raj | United States of America | 2014 | RCT study | Selective-Serotonine-Reuptake Inhibitors (SSRI) | There is no non-pharmacological intervention in this study. | 39 | non-commercial [National Institutes of Health grants; Clinical and Translational] Science Award |
| Moon 2018 | Efficacy of Propranolol, Bisoprolol, and Pyridostigmine for Postural Tachycardia Syndrome: a Randomized Clinical Trial | Jangsup Moon, Do-Yong Kim1, Woo-Jin Lee, Han Sang Lee, Jung-Ah Lim, Tae-Joon Kim,Jin-Sun Jun, Byeongsu Park, Jung-Ick Byun, Jun-Sang Sunwoo, Soon-Tae Lee, Keun-Hwa Jung, Kyung-Il Park, Ki-Young Jung, Manho Kim, Sang Kun Lee, Kon Chu | South Korea | 2018 | RCT study | Beta-Adrenergic-Blocking-Agents; Pyridostigmine | There is no non-pharmacological intervention in this study. | 103 | non-commercial [National Research Foundation of Korea (NRF) funded by the Ministry of Science, ICT & Futur] |
| Nardone 2020 | Effect of a neck compression collar on cardiorespiratory and cerebrovascular function in postural orthostatic tachycardia syndrome (POTS) | Massimo Nardone, Juan Guzman, Paula J. Harvey, John S. Floras, and X Heather Edgell | Canada | 2020 | RCT study | There is no medication in this study. | Other: Neck compression collar (Q-collar) | 10 | non-commercial [Standing up to POTS] |
| Nesheiwat 2020 | Bupropion in the treatment of postural tachycardia syndrome (pots): A single center experience | Rohit Vyas, Zeid Nesheiwat, Mohammed Ruzieh, Zaid Ammari, Mohammad Al-Sarie, Blair Grubb | United States of America | 2020 | Non-RCT study | Buproprion | There is no non-pharmacological intervention in this study. | 194 | not declared |
| Raj 2005 | Acetylcholinesterase inhibition improves tachycardia in postural tachycardia syndrome | Satish R. Raj, MD; Bonnie K. Black, RN, NP; Italo Biaggioni, MD; Paul A. Harris, PhD; David Robertson, MD | United States of America | 2005 | RCT study | Pyridostigmine | There is no non-pharmacological intervention in this study. | 17 | non-commercial [National Institutes of Health grants] |
| Raj 2009 | Propranolol decreases tachycardia and improves symptoms in the postural tachycardia syndrome: less is more | Satish R. Raj, MD, MSCI; Bonnie K. Black, RN, CNP; Italo Biaggioni, MD; Sachin Y. Paranjape, BS; Maricelle Ramirez; William D. Dupont, PhD; David Robertson, MD | United States of America | 2009 | RCT study | Beta-Adrenergic-Blocking-Agents | There is no non-pharmacological intervention in this study. | 54 | non-commercial [National Institutes of Health grants] |
| Rodriguez 2019 | Orthostatic Cognitive Dysfunction in Postural Tachycardia Syndrome After Rapid Water Drinking | Belén Rodriguez, Raya Zimmermann, Klemens Gutbrod, Doerthe Heinemann and Werner J. Z’Graggen | Switzerland | 2019 | Non-RCT study | There is no medication in this study. | (Re-)Hydration | 16 | not given |
| Rodriguez 2022 | Brain fog in neuropathic postural tachycardia syndrome may be associated with autonomic hyperarousal and improves after water drinking | Belén Rodriguez, Annie Hochstrasser, Philippe J. Eugster, Eric Grouzmann, René M. Müri and Werner J. Z’Graggen | Switzerland | 2022 | Non-RCT study | There is no medication in this study. | (Re-)Hydration | 28 | non-commercial [University of Bern (Open access funding)] |
| Ross 2014 | A double-blind placebo-controlled cross-over study of the vascular effects of midodrine in neuropathic compared with hyperadrenergic postural tachycardia syndrome | Amanda J. ROSS, Anthony J. OCON, Marvin S. MEDOW, and Julian M. STEWART | United States of America | 2014 | RCT study | Midodrine | There is no non-pharmacological intervention in this study. | 20 | non-commercial [National Heart, Lung, and Blood Institute; Chronic Fatigue and Immune Deficiency Syndrome (CFIDS) Association] |
| Ruzieh 2017 | Ivabradine in the treatment of postural tachycardia syndrome (POTS), a single center experience | Mohammed Ruzieh, Natalie Sirianni, Zaid Ammari, Osama Dasa, Luai Alhazmi, Beverly Karabin, Blair Grubb | United States of America | 2017 | Non-RCT study | Ivabradine | There is no non-pharmacological intervention in this study. | 66 | None |
| Ruzieh 2017 | Droxidopa in the Treatment of Postural Orthostatic Tachycardia Syndrome | Mohammed Ruzieh, MD, Osama Dasa, MD, Ann Pacenta, MD, Beverly Karabin, CNP, PhD, and Blair Grubb, MD | United States of America | 2016 | Non-RCT study | Droxidopa | There is no non-pharmacological intervention in this study. | 54 | not given |
| Ruzieh 2017 | Effects of intermittent intravenous saline infusions in patients with medication-refractory postural tachycardia syndrome | Mohammed Ruzieh & Aaron Baugh & Osama Dasa & Rachel L. Parker & Joseph T. Perrault & Anas Renno & Beverly L. Karabin & Blair Grubb | United States of America | 2017 | Non-RCT study | There is no medication in this study. | (Re-)Hydration | 72 | None |
| Shibata 2012 | Short-term exercise training improves the cardiovascular response to exercise in the postural orthostatic tachycardia syndrome | Shigeki Shibata, Qi Fu, Tiffany B. Bivens, Jeffrey L. Hastings, Wade Wang and Benjamin D. Levine, | United States of America | 2012 | Non-RCT study | There is no medication in this study. | Physical Exercise / Training | 19 | non-commercial [NASA-NSBRI postdoctoral fellowship grant; NASA-NSBRI career development award; National Institutes of Health;Clinical and Translational Research Center] |
| Smith 2020 | Splanchnic Venous Compression Enhances the Effects of ß-Blockade in the Treatment of Postural Tachycardia Syndrome | Emily C. Smith, André Diedrich, Satish R. Raj, Alfredo Gamboa, Cyndya A. Shibao, Bonnie K. Black, Amanda Peltier, Sachin Y. Paranjape, BS, Italo Biaggioni, and Luis E. Okamoto | Canada; United States of America | 2020 | RCT study | Beta-Adrenergic-Blocking-Agents; Other: Placebo | Compression Garments | 19 | non-commercial [National Institutes of Health (NIH) grants; National Center for Advancing Translational Sciences; American Heart Association grant] |
| Stavrakis 2024 | Noninvasive Vagus Nerve Stimulation in Postural Tachycardia Syndrome: A Randomized Clinical Trial | Stavros Stavrakis, MD, PHD, Praloy Chakraborty, MD, MSC, Kassem Farhat, MD, Seabrook Whyte, MSC, Lynsie Morris, MSC, Zain Ul Abideen Asad, MD, Brittany Karfonta, RN, MPH, Juvaria Anjum, MD, H. Greg Matlock, PHD, Xue Cai, PHD, Xichun Yu, MD | United States of America | 2024 | RCT study | There is no medication in this study. | Transdermal Vagal stimulation | 26 | non-commercial [National Institutes of Health (NIH)/National Heart, Lung, and Blood Institute; NIH/National Institute of General Medical Sciences; Francie Fitzgerald and family through the OU Foundation Fund] |
| Stewart 2021 | Supine Parasympathetic Withdrawal and Upright Sympathetic Activation Underly Abnormalities of the Baroreflex in Postural Tachycardia Syndrome: Effects of Pyridostigmine and Digoxin | Julian M. Stewart, Irfan A. Warsy, Paul Visintainer, Courtney Terilli, Marvin S. Medow | United States of America | 2021 | Non-RCT study | Pyridostigmine; Digoxin | There is no non-pharmacological intervention in this study. | 36 | non-commercial [National Heart Lung and Blood Institute (NHLBI)] |
| Svensson 2024 | Individually tailored exercise in patients with postural orthostatic tachycardia syndrome related to post-COVID-19 condition - a feasibility study | Annie Svensson, Anna Svensson‑Raskh, Linda Holmström Carl Hallberg,  Lucian Bezuidenhout, David Moulaee Conradsson Marcus Ståhlberg,  Judith Bruchfeld, Artur Fedorowski Malin Nygren‑Bonnier | Sweden | 2024 | Non-RCT study | There is no medication in this study. | Physical Exercise / Training | 26 | non-commercial [Karolinska Institute (Open access funding); Swedish Heart Lung Foundation; Swedish Research Council; Swedish Heart and Lung Association; Odd Fellows; Stifelsen Bygg-Göta Vetenskaplig forskning och Social hjälpverksamhet] |
| Taub 2021 | Randomized Trial of Ivabradine in Patients With Hyperadrenergic Postural Orthostatic Tachycardia Syndrome | Pam R. Taub, MD, Adena Zadourian, BS, Hannah C. Lo, BS, Cameron K. Ormiston, BS, Shahrokh Golshan, PHD, Jonathan C. Hsu, MD, MAS | United States of America | 2021 | RCT study | Ivabradine | There is no non-pharmacological intervention in this study. | 26 | None |
| Towheed 2020 | Ivabradine in children with postural orthostatic tachycardia syndrome: a retrospective study | Arooge Towheed, Zeid Nesheiwat, Muhammad A Mangi, Beverly Karabin and Blair P Grubb | United States of America | 2020 | Non-RCT study | Ivabradine | There is no non-pharmacological intervention in this study. | 37 | None |
| Vernino 2024 | Randomized controlled trial of intravenous immunoglobulin for autoimmune postural orthostatic tachycardia syndrome (iSTAND) | Steven Vernino, Steve Hopkins, Meredith Bryarly, Roberto S. Hernandez, Amber Salter | United States of America | 2024 | RCT study | Other: Albumin Intravenous Immunoglobulines (IVIG) | There is no non-pharmacological intervention in this study. | 30 | non-commercial [Dysautonomia International and the Sjogren’s Foundation; Grifols; Roy and Janis Cofee] |
| Wheatley-Guy 2023 | Semi-supervised exercise training program more effective for individuals with postural orthostatic tachycardia syndrome in randomized controlled trial | Courtney M. Wheatley‑Guy, Meredith G. Shea,Jordan K. Parks, Robert Scales, Brent P. Goodman, Richard J. Butterfield, Bruce D. Johnson1 | United States of America | 2023 | RCT study | There is no medication in this study. | Physical Exercise / Training | 60 | non-commercial [Mayo Clinic Arizona Cardiovascular Research Center Clinical Research Grant (MCA CV CRC), Center for Clinical and Translational Science (CCTS), Mayo Clinic] |
| Yang 2013 | Postural orthostatic tachycardia syndrome with increased erythrocytic hydrogen sulfide and response to midodrine hydrochloride | Jinyan Yang, , Juan Zhao, Shuxu Du, Die Liu, Chunhin Fu, Xueying Li,  , Stella Chen, Chaoshu Tang, Junbao Du, and Hongfang Jin, PhD | China | 2013 | Non-RCT study | Midodrine | There is no non-pharmacological intervention in this study. | 56 | National Twelfth 5-Year Plan for Science and Technology Support, Major Basic Research Project of China, National Natural Science Foundation of China (81121061) |
| Z'Graggen 2010 | Acute fluid ingestion in the treatment of orthostatic intolerance - important implications for daily practice | W. J. ZGraggen, C. W. Hess and A. M. Humm | Switzerland | 2010 | Non-RCT study | There is no medication in this study. | (Re-)Hydration | 7 | not given |
| Zha 2023 | Gluten-free diet in postural orthostatic tachycardia syndrome (POTS) | Kate Zha BS, Jill Brook MA, Abigail McLaughlin MD and Svetlana Blitshteyn MD | United States of America | 2023 | Non-RCT study | There is no medication in this study. | Gluten-free diet | 20 | None |
| Zhang 2012 | Midregional pro-adrenomedullin as a predictor for therapeutic response to midodrine hydrochloride in children with postural orthostatic tachycardia syndrome | Fengwen Zhang, MM, Xueying Li, MM, Todd Ochs, MD, Li Chen, MM, Ying Liao, MD, Chaoshu Tang, PHD, Hongfang Jin, PHD, Junbao Du, MD | China; United States of America | 2012 | Non-RCT study | Midodrine | There is no non-pharmacological intervention in this study. | 77 | non-commercial [Major Basic Research Project of China; National Twelfth Five-Year Plan for Science & Technology Support; Beijing Science and Technology Project, China; National Natural Science Foundation of China] |
| Zhao 2014 | Usefulness of plasma copeptin as a biomarker to predict the therapeutic effectiveness of metoprolol for postural tachycardia syndrome in children | Juan Zhao, PhD, Shuxu Du, Jinyan Yang, Jing Lin, Chaoshu Tang, Junbao Du, and Hongfang Jin, PhD | China | 2014 | Non-RCT study | Beta-Adrenergic-Blocking-Agents | There is no non-pharmacological intervention in this study. | 74 | non-commercial [National Twelfth Five-Year Plan for Science & Technology Support, Beijing; Major Basic Research Project of China, Beijing; National Natural Science Foundation of China, Beijing] |
|  |  |  |  |  |  |  |  |  |  |
|  | ***Systematic Literature Review - Treatment of Postural Orthostatic Tachycardia Syndrome (POTS)*; Clinical Autonomic Research; Authors: Nicole Schiweck, Katharina Langer, Andrea Maier, Daniel Vilser, Juliane Spiegler; University Hospital Wuerzburg; Corresponding author: Juliane Spiegler (spiegler_j@ukw.de)** | | | | |  |  |  |  |
|  |  |  |  |  |  |  |  |  |  |
|  |  |  |  |  |  |  |  |  |  |
