# Supplementary material for: Systematic literature review: treatment of postural orthostatic tachycardia syndrome (POTS)
Source: Clin Auton Res. 2025 Nov 12;36(1):3–16. doi: 10.1007/s10286-025-01172-2 (PMC12982215; doi:10.1007/s10286-025-01172-2)
Supplement: Supplementary file 4 — Supplementary file4 (DOCX 53 kb) [file 10286_2025_1172_MOESM4_ESM.docx]

Systematic Literature Review - Treatment of Postural Orthostatic Tachycardia Syndrome (POTS); Clinical Autonomic Research; Authors: Nicole Schiweck, Katharina Langer, Andrea Maier, Daniel Vilser, Juliane Spiegler; University Hospital Wuerzburg; Corresponding author: Juliane Spiegler (spiegler_j@ukw.de)
