# Supplementary material for: Systematic literature review: treatment of postural orthostatic tachycardia syndrome (POTS)
Source: Clin Auton Res. 2025 Nov 12;36(1):3–16. doi: 10.1007/s10286-025-01172-2 (PMC12982215; doi:10.1007/s10286-025-01172-2)
Supplement: Supplementary file 6 — Supplementary file6 (DOCX 87 kb) [file 10286_2025_1172_MOESM6_ESM.docx]

| **Supplement 6 - Excluded Studies** | | | | | |
| --- | --- | --- | --- | --- | --- |
| **Title** | **Authors** | **Published Year** | **Journal** | **DOI** | **Reason** |
| Erratum: Brain fog in neuropathic postural tachycardia syndrome may be associated with autonomic hyperarousal and improves after water drinking |  | 2023 | Front Neurosci | 10.3389/fnins.2023.1178850 | Exclusion reason: Wrong study design; |
| Ivabradine effects on COVID-19-associated postural orthostatic tachycardia syndrome: a single center prospective study | Abdelnabi, M.; Saleh, Y.; Ahmed, A.; Benjanuwattra, J.; Leelaviwat, N.; Almaghraby, A. | 2023 | Am J Cardiovasc Dis |  | Exclusion reason: Wrong outcomes; |
| Heart rate and plasma cyclic AMP responses to isoproterenol infusion and effect of beta-adrenergic blockade in patients with postural orthostatic tachycardia syndrome | Abe, H.; Nagatomo, T.; Kohshi, K.; Numata, T.; Kikuchi, K.; Sonoda, S.; Mizuki, T.; Kuroiwa, A.; Nakashima, Y. | 2000 | J Cardiovasc Pharmacol | 10.1097/00005344-200000006-00017 | Exclusion reason: Wrong outcomes; |
| [Evaluation of the vagal activity by the Deep-Breathing test] | Aboudrar, S.; Benjelloun, H.; Benazzouz, A.; Bendahmanne, S.; Coghlan, L.; Kanouni, N.; Abouqal, R.; Benomar, M. | 2007 | Neurophysiol Clin | 10.1016/j.neucli.2007.01.005 | Exclusion reason: Wrong language; |
| Low-dose propranolol and exercise capacity in postural tachycardia syndrome: a randomized study | Arnold, A. C.; Okamoto, L. E.; Diedrich, A.; Paranjape, S. Y.; Raj, S. R.; Biaggioni, I.; Gamboa, A. | 2013 | Neurology | 10.1212/WNL.0b013e318293e310 | Exclusion reason: Wrong outcomes; |
| Letter: Propranolol in orthostatic tachycardia | Barch, F.; Lawrence, T. | 1975 | Am Heart J | 10.1016/0002-8703(75)90168-4 | Exclusion reason: Wrong study design; |
| Improved Acute Orthostatic Tolerance in POTS by Lower Body Compression: Both Beneficial and Sufficient? | Benditt, D. G.; Sutton, R. | 2021 | J Am Coll Cardiol | 10.1016/j.jacc.2020.11.039 | Exclusion reason: Wrong study design; |
| The effect of propranolol on ECG in angina pectoris and orthostatic tachycardia | Biörck, G.; Eliasch, H.; Pernow, B.; Rosén, A. | 1968 | Acta Med Scand | 10.1111/j.0954-6820.1968.tb02456.x | Exclusion reason: Wrong patient population; |
| Postural orthostatic tachycardia syndrome (POTS) and other autonomic disorders after COVID-19 infection: a case series of 20 patients | Blitshteyn, S.; Whitelaw, S. | 2021 | Immunol Res | 10.1007/s12026-021-09185-5 | Exclusion reason: Wrong study design; |
| Correction to: Postural orthostatic tachycardia syndrome (POTS) and other autonomic disorders after COVID-19 infection: a case series of 20 patients | Blitshteyn, S.; Whitelaw, S. | 2021 | Immunol Res | 10.1007/s12026-021-09191-7 | Exclusion reason: Wrong study design; |
| Utilisation of medications to reduce symptoms in children with postural orthostatic tachycardia syndrome | Boris, J. R.; Bernadzikowski, T. | 2018 | Cardiol Young | 10.1017/s1047951118001373 | Exclusion reason: Wrong setting; |
| Therapy for fatigue and cognitive dysfunction in postural orthostatic tachycardia syndrome | Boris, J. R.; Bernadzikowski, T. | 2018 | Cardiol Young | 10.1017/s1047951118001415 | Exclusion reason: Wrong setting; |
| Clinical Course of Transgender Adolescents with Complicated Postural Orthostatic Tachycardia Syndrome Undergoing Hormonal Therapy in Gender Transition: A Case Series | Boris, J. R.; McClain, Z. B. R.; Bernadzikowski, T. | 2019 | Transgend Health | 10.1089/trgh.2019.0041 | Exclusion reason: Wrong patient population; |
| Do meals affect heart rate and symptoms in postural orthostatic tachycardia syndrome? | Bourne, K. M.; Stiles, L. E.; Raj, S. R.; Shibao, C. A. | 2022 | Clin Auton Res | 10.1007/s10286-021-00835-0 | Exclusion reason: Wrong study design; |
| Long-term control of abdominal pain related to POTS: two case reports in pediatrics | Brown, B.; Naber, J. W.; Kapural, L. | 2018 | Pain Manag | 10.2217/pmt-2018-0013 | Exclusion reason: Wrong study design; |
| Improvement in Functioning and Psychological Distress in Adolescents With Postural Orthostatic Tachycardia Syndrome Following Interdisciplinary Treatment | Bruce, B. K.; Harrison, T. E.; Bee, S. M.; Luedtke, C. A.; Porter, C. J.; Fischer, P. R.; Hayes, S. E.; Allman, D. A.; Ale, C. M.; Weiss, K. E. | 2016 | Clin Pediatr (Phila) | 10.1177/0009922816638663 | Exclusion reason: Wrong outcomes; |
| Therapeutic Approaches to Dysautonomia in Childhood, with a Special Focus on Long COVID | Buchhorn, R. | 2023 | Children (Basel) | 10.3390/children10020316 | Exclusion reason: Data not clearly presented; |
| [Progress in the treatment of postural tachycardia syndrome with midodrine hydrochloride in children] | Chen, J.; Du, J. | 2015 | Zhonghua Er Ke Za Zhi |  | Exclusion reason: Wrong language; |
| [Effect of selective alpha1 receptor agonist in the treatment of children with postural orthostatic tachycardia syndrome] | Chen, L.; Du, J. B.; Jin, H. F.; Zhang, Q. Y.; Li, W. Z.; Wang, L.; Wang, Y. L. | 2008 | Zhonghua Er Ke Za Zhi |  | Exclusion reason: Wrong language; |
| Baroreflex sensitivity predicts therapeutic effects of metoprolol on pediatric postural orthostatic tachycardia syndrome | Cui, Y.; Wang, Y.; Liu, P.; Wang, Y.; Du, J.; Jin, H. | 2022 | Front Cardiovasc Med | 10.3389/fcvm.2022.930994 | Exclusion reason: Wrong study design; |
| Sinus node sparing novel hybrid approach for treatment of inappropriate sinus tachycardia/postural sinus tachycardia: multicenter experience | de Asmundis, C.; Chierchia, G. B.; Lakkireddy, D.; Romeya, A.; Okum, E.; Gandhi, G.; Sieira, J.; Vloka, M.; Jones, S. D.; Shah, H.; Winner, M.; Patel, D.; Whalen, S. P.; Beaty, E. H.; Kincaid, E. H.; Lee, A.; Brodt, C.; Taylor, B. J.; Colombowala, I.; Romano, M.; Morady, F.; Ströker, E.; Overeinder, I.; Bala, G.; Van Meeteren, J.; Krauthammer, Y.; Koerber, S.; Shults, C.; Thomaides, A.; Badhwar, N.; Gopinathannair, R.; Shah, A.; Tummala, R.; Bello, D.; Hoff, S.; Almorad, A.; Frazier, K.; Brugada, P.; La Meir, M. | 2022 | J Interv Card Electrophysiol | 10.1007/s10840-021-01044-5 | Exclusion reason: Wrong outcomes; |
| Sinus Node Sparing Novel Hybrid Approach for Treatment of Inappropriate Sinus Tachycardia/Postural Orthostatic Sinus Tachycardia With New Electrophysiological Finding | de Asmundis, C.; Chierchia, G. B.; Sieira, J.; Ströker, E.; Umbrain, V.; Poelaert, J.; Brugada, P.; La Meir, M. | 2019 | Am J Cardiol | 10.1016/j.amjcard.2019.04.019 | Exclusion reason: Wrong outcomes; |
| Redo procedures after sinus node sparing hybrid ablation for inappropriate sinus tachycardia/postural orthostatic sinus tachycardia | de Asmundis, C.; Marcon, L.; Pannone, L.; Della Rocca, D. G.; Lakkireddy, D.; Beaver, T. M.; Brodt, C. R.; Monaco, C.; Sorgente, A.; Audiat, C.; Vetta, G.; Ramak, R.; Overeinder, I.; Kronenberger, R.; Bala, G.; Almorad, A.; Ströker, E.; Sieira, J.; Sarkozy, A.; Brugada, P.; Chierchia, G. B.; La Meir, M. | 2023 | Europace | 10.1093/europace/euad373 | Exclusion reason: Wrong study design; |
| Ivabradine in Postural Orthostatic Tachycardia Syndrome: Preliminary Experience in Children | Delle Donne, G.; Rosés Noguer, F.; Till, J.; Salukhe, T.; Prasad, S. K.; Daubeney, P. E. F. | 2018 | Am J Cardiovasc Drugs | 10.1007/s40256-017-0248-x | Exclusion reason: Wrong outcomes; |
| Difference between supine and upright blood pressure associates to the efficacy of midodrine on postural orthostatic tachycardia syndrome (POTS) in children | Deng, W.; Liu, Y.; Liu, A. D.; Holmberg, L.; Ochs, T.; Li, X.; Yang, J.; Tang, C.; Du, J.; Jin, H. | 2014 | Pediatr Cardiol | 10.1007/s00246-013-0843-9 | Exclusion reason: Wrong study design; |
| Postural orthostatic tachycardia syndrome after mRNA COVID-19 vaccine | Eldokla, A. M.; Numan, M. T. | 2022 | Clin Auton Res | 10.1007/s10286-022-00880-3 | Exclusion reason: Wrong study design; |
| Immediate cardiovascular responses to oral prazosin--effects of concurrent beta-blockers | Elliott, H. L.; McLean, K.; Sumner, D. J.; Meredith, P. A.; Reid, J. L. | 1981 | Clin Pharmacol Ther | 10.1038/clpt.1981.40 | Exclusion reason: Wrong patient population; |
| Symptom improvement in postural orthostatic tachycardia syndrome with the sinus node blocker ivabradine | Ewan, V.; Norton, M.; Newton, J. L. | 2007 | Europace | 10.1093/europace/eum235 | Exclusion reason: Wrong study design; |
| Acute volume loading and exercise capacity in postural tachycardia syndrome | Figueroa, R. A.; Arnold, A. C.; Nwazue, V. C.; Okamoto, L. E.; Paranjape, S. Y.; Black, B. K.; Diedrich, A.; Robertson, D.; Biaggioni, I.; Raj, S. R.; Gamboa, A. | 2014 | J Appl Physiol (1985) | 10.1152/japplphysiol.00367.2014 | Exclusion reason: Wrong outcomes; |
| Use of an allostatic neurotechnology by adolescents with postural orthostatic tachycardia syndrome (POTS) is associated with improvements in heart rate variability and changes in temporal lobe electrical activity | Fortunato, J. E.; Tegeler, C. L.; Gerdes, L.; Lee, S. W.; Pajewski, N. M.; Franco, M. E.; Cook, J. F.; Shaltout, H. A.; Tegeler, C. H. | 2016 | Exp Brain Res | 10.1007/s00221-015-4499-y | Exclusion reason: Wrong outcomes; |
| Mindfulness-Based Stress Reduction and Group Support Decrease Stress in Adolescents with Cardiac Diagnoses: A Randomized Two-Group Study | Freedenberg, V. A.; Hinds, P. S.; Friedmann, E. | 2017 | Pediatr Cardiol | 10.1007/s00246-017-1679-5 | Exclusion reason: Wrong patient population; |
| Exercise in the postural orthostatic tachycardia syndrome | Fu, Q.; Levine, B. D. | 2015 | Auton Neurosci | 10.1016/j.autneu.2014.11.008 | Exclusion reason: Wrong study design; |
| Exercise training versus propranolol in the treatment of the postural orthostatic tachycardia syndrome | Fu, Q.; Vangundy, T. B.; Shibata, S.; Auchus, R. J.; Williams, G. H.; Levine, B. D. | 2011 | Hypertension | 10.1161/hypertensionaha.111.172262 | Exclusion reason: Wrong outcomes;  ; |
| Effects of exercise training on arterial-cardiac baroreflex function in POTS | Galbreath, M. M.; Shibata, S.; VanGundy, T. B.; Okazaki, K.; Fu, Q.; Levine, B. D. | 2011 | Clin Auton Res | 10.1007/s10286-010-0091-5 | Exclusion reason: Wrong outcomes; |
| Symptomatic use of carbidopa in autonomic disorders | Golden, E. P.; Park, C. J.; Vernino, S. | 2021 | Auton Neurosci | 10.1016/j.autneu.2021.102888 | Exclusion reason: Wrong patient population; |
| High Sodium Intake in Patients With Postural Orthostatic Tachycardia Syndrome: A Practice "Worth Its Salt" | Grubb, B. P. | 2021 | J Am Coll Cardiol | 10.1016/j.jacc.2021.03.229 | Exclusion reason: Wrong study design; |
| Adalimumab as a potential treatment for postural orthostatic tachycardia syndrome | Hendrix, A.; Nesheiwat, Z.; Towheed, A.; Brar, V.; Grubb, B. P. | 2021 | HeartRhythm Case Rep | 10.1016/j.hrcr.2020.11.003 | Exclusion reason: Wrong study design; |
| Treatment of autonomic neuropathy, postural tachycardia and orthostatic syncope with octreotide LAR | Hoeldtke, R. D.; Bryner, K. D.; Hoeldtke, M. E.; Hobbs, G. | 2007 | Clin Auton Res | 10.1007/s10286-007-0436-x | Exclusion reason: Wrong patient population; |
| The orthostatic tachycardia syndrome: evaluation of autonomic function and treatment with octreotide and ergot alkaloids | Hoeldtke, R. D.; Davis, K. M. | 1991 | J Clin Endocrinol Metab | 10.1210/jcem-73-1-132 | Exclusion reason: Wrong study design; |
| Treatment of orthostatic tachycardia with erythropoietin | Hoeldtke, R. D.; Horvath, G. G.; Bryner, K. D. | 1995 | Am J Med | 10.1016/s0002-9343(99)80230-7 | Exclusion reason: Wrong outcomes; |
| [Postural orthostatic tachycardia syndrome (POTS): report of 15 cases] | Jiménez-Cohl, P.; Earle, N. M.; González, B. R.; Thieck, E. J. | 2012 | Rev Med Chil | 10.4067/s0034-98872012000200001 | Exclusion reason: Wrong language; |
| Long-Haul Post-COVID-19 Symptoms Presenting as a Variant of Postural Orthostatic Tachycardia Syndrome: The Swedish Experience | Johansson, M.; Ståhlberg, M.; Runold, M.; Nygren-Bonnier, M.; Nilsson, J.; Olshansky, B.; Bruchfeld, J.; Fedorowski, A. | 2021 | JACC Case Rep | 10.1016/j.jaccas.2021.01.009 | Exclusion reason: Wrong study design; |
| Exercise training in Postural Orthostatic Tachycardia syndrome: blocking the urge to block β-receptors? | Joyner, M. J. | 2011 | Hypertension | 10.1161/hypertensionaha.111.173872 | Exclusion reason: Wrong study design; |
| Youth with Chronic Pain and Postural Orthostatic Tachycardia Syndrome (POTS): Treatment Mediators of Improvement in Functional Disability | Junghans-Rutelonis, A. N.; Craner, J. R.; Ale, C. M.; Harbeck-Weber, C.; Fischer, P. R.; Weiss, K. E. | 2018 | J Clin Psychol Med Settings | 10.1007/s10880-018-9558-7 | Exclusion reason: Wrong outcomes; |
| Further Observations on the Use of Pacemakers in Patients with Postural Orthostatic Tachycardia Syndrome with Demonstrated Asystole | Kanjwal, K.; Kichloo, A.; Qadir, R.; Grubb, B. P. | 2021 | J Innov Card Rhythm Manag | 10.19102/icrm.2021.120307 | Exclusion reason: Wrong study design; |
| Role of implantable loop recorders in patients with postural orthostatic tachycardia syndrome | Kanjwal, K.; Qadir, R.; Ruzieh, M.; Grubb, B. P. | 2018 | Pacing Clin Electrophysiol | 10.1111/pace.13441 | Exclusion reason: Wrong study design; |
| Use of methylphenidate in the treatment of patients suffering from refractory postural tachycardia syndrome | Kanjwal, K.; Saeed, B.; Karabin, B.; Kanjwal, Y.; Grubb, B. P. | 2012 | Am J Ther | 10.1097/MJT.0b013e3181dd21d2 | Exclusion reason: Wrong outcomes; |
| Use of octreotide in the treatment of refractory orthostatic intolerance | Kanjwal, K.; Saeed, B.; Karabin, B.; Kanjwal, Y.; Grubb, B. P. | 2012 | Am J Ther | 10.1097/MJT.0b013e3181e28156 | Exclusion reason: Wrong patient population; |
| Active compression garment prevents tilt-induced orthostatic tachycardia in humans | Kelly, K. L.; Johnson, C. P.; Dunne, L. E.; Holschuh, B.; Joyner, M.; Johnson, B. D. | 2019 | Physiol Rep | 10.14814/phy2.14050 | Exclusion reason: Wrong patient population; |
| Immunotherapy with subcutaneous immunoglobulin or plasmapheresis in patients with postural orthostatic tachycardia syndrome (POTS) | Kesterson, K.; Schofield, J.; Blitshteyn, S. | 2023 | J Neurol | 10.1007/s00415-022-11344-z | Exclusion reason: Wrong study design; |
| Treatment of Refractory Postural Tachycardia Syndrome with Subcutaneous Octreotide Delivered Using an Insulin Pump | Khan, M.; Ouyang, J.; Perkins, K.; Somauroo, J.; Joseph, F. | 2015 | Case Rep Med | 10.1155/2015/545029 | Exclusion reason: Wrong study design; |
| Experimental induction of panic-like symptoms in patients with postural tachycardia syndrome | Khurana, R. K. | 2006 | Clin Auton Res | 10.1007/s10286-006-0365-0 | Exclusion reason: Wrong study design; |
| Outcomes in adolescents with postural orthostatic tachycardia syndrome treated with midodrine and beta-blockers | Lai, C. C.; Fischer, P. R.; Brands, C. K.; Fisher, J. L.; Porter, C. B.; Driscoll, S. W.; Graner, K. K. | 2009 | Pacing Clin Electrophysiol | 10.1111/j.1540-8159.2008.02207.x | Exclusion reason: Wrong outcomes; |
| Virtual patient workshops: A tool for education, community, and empowerment in patients with postural orthostatic tachycardia syndrome | Laird-Gion, J.; Kelley-Hedgepeth, A.; Lee Lewis, D. | 2022 | Heart Rhythm O2 | 10.1016/j.hroo.2022.02.015 | Exclusion reason: Wrong study design; |
| Body Mass Index (BMI) is Associated with the Therapeutic Response to Oral Rehydration Solution in Children with Postural Tachycardia Syndrome | Li, H.; Wang, Y.; Liu, P.; Chen, Y.; Feng, X.; Tang, C.; Du, J.; Jin, H. | 2016 | Pediatr Cardiol | 10.1007/s00246-016-1436-1 | Exclusion reason: Wrong study design; |
| [Predictive value of baseline plasma midregional fragment of pro-adrenomedullin level on long-term outcome of postural tachycardia syndrome children treated with midodrine hydrochloride] | Li, H.; Zhang, F.; Wang, Y.; Liu, P.; Zhang, C.; Feng, X.; Du, J.; Jin, H. | 2015 | Zhonghua Xin Xue Guan Bing Za Zhi |  | Exclusion reason: Wrong language; |
| [Predictive value of upright blood pressure change for long-term prognosis of children with postural tachycardia syndrome treated with midodrine hydrochloride] | Li, H. X.; Deng, W. J.; Zhang, C. Y.; Jin, H. F.; Du, J. B. | 2016 | Zhonghua Er Ke Za Zhi | 10.3760/cma.j.issn.0578-1310.2016.07.009 | Exclusion reason: Wrong language; |
| Clinical features and management of postural tachycardia syndrome in children: a single-center experience | Li, J.; Zhang, Q.; Hao, H.; Jin, H.; Du, J. | 2014 | Chin Med J (Engl) |  | Exclusion reason: Wrong outcomes; |
| [Clinical value of 24-hour urinary sodium determination in children with postural tachycardia syndrome] | Li, J.; Zhang, Q.; Liao, Y.; Zhang, C.; Du, J. | 2015 | Zhonghua Er Ke Za Zhi |  | Exclusion reason: Wrong language; |
| Prognostic analysis of orthostatic intolerance using survival model in children | Li, Y.; Li, H.; Li, X.; Li, X.; Jin, H. | 2014 | Chin Med J (Engl) |  | Exclusion reason: Wrong study design; |
| Flow-mediated vasodilation as a predictor of therapeutic response to midodrine hydrochloride in children with postural orthostatic tachycardia syndrome | Liao, Y.; Yang, J.; Zhang, F.; Chen, S.; Liu, X.; Zhang, Q.; Ai, Y.; Wang, Y.; Tang, C.; Du, J.; Jin, H. | 2013 | Am J Cardiol | 10.1016/j.amjcard.2013.05.008 | Exclusion reason: Wrong study design; |
| Plasma C-type natriuretic peptide as a predictor for therapeutic response to metoprolol in children with postural tachycardia syndrome | Lin, J.; Han, Z.; Li, H.; Chen, S. Y.; Li, X.; Liu, P.; Wang, Y.; Tang, C.; Du, J.; Jin, H. | 2015 | PLoS One | 10.1371/journal.pone.0121913 | Exclusion reason: Wrong study design; |
| [Evaluation of the changes in heart rate during head-up test predicting the efficacy of oral rehydration salts on postural tachycardia syndrome in children] | Lin, J.; Liu, P.; Wang, Y.; Li, H.; Li, X.; Zhao, J.; Tang, C.; Du, J.; Jin, H. | 2015 | Zhonghua Er Ke Za Zhi |  | Exclusion reason: Wrong study design; |
| Salivary Cortisol Levels Predict Therapeutic Response to a Sleep-Promoting Method in Children with Postural Tachycardia Syndrome | Lin, J.; Zhao, H.; Shen, J.; Jiao, F. | 2017 | J Pediatr | 10.1016/j.jpeds.2017.08.039 | Exclusion reason: Wrong study design; |
| Observational case series describing features of cardiopulmonary exercise testing in Postural Tachycardia Syndrome (PoTS) | Loughnan, A.; Gall, N.; James, S. | 2021 | Auton Neurosci | 10.1016/j.autneu.2020.102762 | Exclusion reason: Wrong study design; |
| [Autonomic function evaluation and physical treatment in children with POTS] | Lu, W.; Du, J. | 2015 | Zhonghua Er Ke Za Zhi |  | Exclusion reason: Wrong language; |
| Electrocardiography-Derived Predictors for Therapeutic Response to Treatment in Children with Postural Tachycardia Syndrome | Lu, W.; Yan, H.; Wu, S.; Chen, S.; Xu, W.; Jin, H.; Du, J. | 2016 | J Pediatr | 10.1016/j.jpeds.2016.05.030 | Exclusion reason: Wrong study design; |
| Hemocytometric Measures Predict the Efficacy of Oral Rehydration for Children with Postural Tachycardia Syndrome | Lu, W.; Yan, H.; Wu, S.; Xu, W.; Jin, H.; Du, J. | 2017 | J Pediatr | 10.1016/j.jpeds.2017.04.034 | Exclusion reason: Wrong study design; |
| Cognitive functioning in postural orthostatic tachycardia syndrome among different body positions: a prospective pilot study (POTSKog study) | Maier, A.; Schopen, L.; Thiel, J. C.; Müller, K.; Fimm, B.; Schulz, J. B. | 2023 | Clin Auton Res | 10.1007/s10286-023-00950-0 | Exclusion reason: Wrong study design; |
| Single centre experience of ivabradine in postural orthostatic tachycardia syndrome | McDonald, C.; Frith, J.; Newton, J. L. | 2011 | Europace | 10.1093/europace/euq390 | Exclusion reason: Wrong study design; |
| Radiofrequency venous ablation for symptomatic relief in postural orthostatic tachycardia syndrome: a case series | McGeoch, C. L. B.; Steinberg, R. S.; Bortfeld, K. S.; Almuwaqqat, Z.; Rheudasil, J. M.; Bhatia, N. K.; Cutchins, A. C. | 2024 | Eur Heart J Case Rep | 10.1093/ehjcr/ytae029 | Exclusion reason: Wrong study design; |
| The Benefits of Oral Rehydration on Orthostatic Intolerance in Children with Postural Tachycardia Syndrome | Medow, M. S.; Guber, K.; Chokshi, S.; Terilli, C.; Visintainer, P.; Stewart, J. M. | 2019 | J Pediatr | 10.1016/j.jpeds.2019.07.041 | Exclusion reason: Wrong outcomes; |
| Orthostatic intolerance in the young and the benefits of oral rehydration | Medow, M. S.; Guber, K.; Chokshi, S.; Terilli, C.; Visintainer, P.; Stewart, J. M. | 2019 | Clinical Autonomic Research | https://dx.doi.org/10.1007/s10286-019-00631-x | Exclusion reason: Wrong outcomes; |
| Phenylephrine Alters Phase Synchronization between Cerebral Blood Velocity and Blood Pressure in Chronic Fatigue Syndrome with Orthostatic Intolerance | Medow, M. S.; Stewart, J. M. | 2024 | Am J Physiol Regul Integr Comp Physiol | 10.1152/ajpregu.00071.2024 | Exclusion reason: Wrong outcomes; |
| Ivabradine in POTS: Increase in Central Pressure Rather Than Down-Regulation of Sympathetic Nervous System | Messerli, F. H.; Seiler, C.; Rimoldi, S. F. E. | 2021 | J Am Coll Cardiol | 10.1016/j.jacc.2021.03.338 | Exclusion reason: Wrong study design; |
| Towards more evidenced-based therapies for postural tachycardia syndrome and other updates on recent autonomic research | Miglis, M. G.; Muppidi, S. | 2021 | Clin Auton Res | 10.1007/s10286-021-00795-5 | Exclusion reason: Wrong study design; |
| Abdominal Compression as a Treatment for Postural Tachycardia Syndrome | Miller, A. J.; Bourne, K. M. | 2020 | J Am Heart Assoc | 10.1161/jaha.120.017610 | Exclusion reason: Wrong study design; |
| Propranolol in the treatment of orthostatic tachycardia associated with orthostatic hypotension | Miller, A. J.; Cohen, H. C.; Glick, G. | 1974 | Am Heart J | 10.1016/0002-8703(74)90211-7 | Exclusion reason: Wrong study design; |
| Intravenous Hydration for Management of Medication-Resistant Orthostatic Intolerance in the Adolescent and Young Adult | Moak, J. P.; Leong, D.; Fabian, R.; Freedenberg, V.; Jarosz, E.; Toney, C.; Hanumanthaiah, S.; Darbari, A. | 2016 | Pediatr Cardiol | 10.1007/s00246-015-1274-6 | Exclusion reason: Wrong patient population; |
| Erratum to: Ablation therapy of postural orthostatic tachycardia syndrome, inappropriate sinus tachycardia and primary electrical diseases: new insights in invasive treatment options in severely symptomatic patients | Monaco, C.; Sorgente, A.; Ramak, R.; Chierchia, G. B.; La Meir, M.; de Asmundis, C. | 2021 | Herzschrittmacherther Elektrophysiol | 10.1007/s00399-021-00799-8 | Exclusion reason: Wrong study design; |
| Response to Letter: Postural Orthostatic Tachycardia Syndrome in Children-A Single-Center Study | Moodley, M. | 2021 | J Child Neurol | 10.1177/0883073821993609 | Exclusion reason: Wrong study design; |
| Head-down tilt reduces the heart rate in postural tachycardia syndrome in acute setting: a pilot study | Novak, P. | 2024 | Neurol Sci | 10.1007/s10072-023-07153-5 | Exclusion reason: Wrong intervention; |
| Understanding the placebo effect in clinical trials for postural tachycardia syndrome | Nwazue, V. C.; Arnold, A. C.; Raj, V.; Black, B. K.; Biaggioni, I.; Paranjape, S. Y.; Orozco, C.; Dupont, W. D.; Robertson, D.; Raj, S. R. | 2014 | Clin Exp Pharmacol Physiol | 10.1111/1440-1681.12221 | Exclusion reason: Wrong study design; |
| Postural tachycardia syndrome and inappropriate sinus tachycardia: role of autonomic modulation and sinus node automaticity | Nwazue, V. C.; Paranjape, S. Y.; Black, B. K.; Biaggioni, I.; Diedrich, A.; Dupont, W. D.; Robertson, D.; Raj, S. R. | 2014 | J Am Heart Assoc | 10.1161/jaha.113.000700 | Exclusion reason: Wrong outcomes; |
| The Goals and Outcomes of Adolescent and Young Adults with POTS Attending an Intensive Interdisciplinary Treatment Program | Oetjen, L.; Johannsen, A.; Bean, J.; Sim, L.; Harrison, T.; Tsai Owens, M.; Harbeck-Weber, C. | 2022 | Occup Ther Health Care | 10.1080/07380577.2022.2116666 | Exclusion reason: Wrong setting; |
| COVID-19 and postural tachycardia syndrome: a case series | Parker, W. H.; Moudgil, R.; Wilson, R. G.; Tonelli, A. R.; Mayuga, K. A.; Singh, T. K. | 2021 | Eur Heart J Case Rep | 10.1093/ehjcr/ytab325 | Exclusion reason: Wrong study design; |
| Orthostatic intolerance without postural tachycardia: how much dysautonomia? | Parsaik, A. K.; Singer, W.; Allison, T. G.; Sletten, D. M.; Joyner, M. J.; Benarroch, E. E.; Low, P. A.; Sandroni, P. | 2013 | Clin Auton Res | 10.1007/s10286-013-0199-5 | Exclusion reason: Wrong study design; |
| Co-creation of a complex, multicomponent rehabilitation intervention and feasibility trial protocol for the PostUraL tachycardia Syndrome Exercise (PULSE) study | Pearce, G.; Holliday, N.; Sandhu, H.; Eftekhari, H.; Bruce, J.; Timms, E.; Ablett, L.; Kavi, L.; Simmonds, J.; Evans, R.; Magee, P.; Powell, R.; Keogh, S.; McGregor, G. | 2023 | Pilot Feasibility Stud | 10.1186/s40814-023-01365-4 | Exclusion reason: Wrong study design; |
| The influence of sex on the treatment of postural tachycardia syndrome in children | Peng, Y.; Wang, S.; Zou, R.; Cai, H.; Zhang, J.; Wang, Y.; Wang, C. | 2023 | Medicine (Baltimore) | 10.1097/md.0000000000033951 | Exclusion reason: Wrong study design; |
| Vagal nerve stimulation is beneficial in postural orthostatic tachycardia syndrome and epilepsy | Petelin Gadze, Z.; Bujan Kovac, A.; Adamec, I.; Milekic, N.; Sulentic, V. | 2018 | Seizure | 10.1016/j.seizure.2018.03.001 | Exclusion reason: Wrong study design; |
| Breathlessness and dysfunctional breathing in patients with postural orthostatic tachycardia syndrome (POTS): The impact of a physiotherapy intervention | Reilly, C. C.; Floyd, S. V.; Lee, K.; Warwick, G.; James, S.; Gall, N.; Rafferty, G. F. | 2020 | Auton Neurosci | 10.1016/j.autneu.2019.102601 | Exclusion reason: Wrong outcomes; |
| Immunomodulatory treatment in postural tachycardia syndrome: A case series | Rodriguez, B.; Hoepner, R.; Salmen, A.; Kamber, N.; Z'Graggen, W. J. | 2021 | Eur J Neurol | 10.1111/ene.14711 | Exclusion reason: Wrong study design; |
| Mechanisms of blood pressure alterations in response to the Valsalva maneuver in postural tachycardia syndrome | Sandroni, P.; Novak, V.; Opfer-Gehrking, T. L.; Huck, C. A.; Low, P. A. | 2000 | Clin Auton Res | 10.1007/bf02291382 | Exclusion reason: Wrong study design; |
| Postural tachycardia syndrome: clinical features and follow-up study | Sandroni, P.; Opfer-Gehrking, T. L.; McPhee, B. R.; Low, P. A. | 1999 | Mayo Clin Proc | 10.4065/74.11.1106 | Exclusion reason: Wrong intervention; |
| [Orthostatic postural tachycardia: study of 8 patients] | Santiago Pérez, S.; Ferrer Gila, T. | 1998 | Med Clin (Barc) |  | Exclusion reason: Wrong language; |
| Intravenous Immunoglobulin Therapy in Refractory Autoimmune Dysautonomias: A Retrospective Analysis of 38 Patients | Schofield, J. R.; Chemali, K. R. | 2019 | Am J Ther | 10.1097/mjt.0000000000000778 | Exclusion reason: Wrong study design; |
| Emerging Role of Autonomic Modulation by Transcutaneous Vagus Nerve Stimulation: Electrifying Hope in POTS? | Seeley, M. C.; Gallagher, C.; Lau, D. H. | 2024 | JACC Clin Electrophysiol | 10.1016/j.jacep.2023.11.024 | Exclusion reason: Wrong study design; |
| Plasma Exchange Improves Cognitive Function in Long-COVID-Related Postural Orthostatic Tachycardia Syndrome and Autoimmune Neurological Dysfunction | Seeley, M. C.; Hooper, M.; Tan, J.; Wells, R.; Gallagher, C.; Lau, D. H. | 2023 | Am J Med | 10.1016/j.amjmed.2023.01.043 | Exclusion reason: Wrong study design; |
| Desmopressin as a Novel Long-Term Treatment in Postural Tachycardia Syndrome Patients with Polyuria | Seeley, M. C.; Thynne, T. R.; Braund, W. J.; Worthley, D. L.; Gallagher, C.; Sanders, P.; Lau, D. H. | 2021 | Am J Med | 10.1016/j.amjmed.2021.03.028 | Exclusion reason: Wrong study design; |
| Water drinking as a treatment for orthostatic syndromes | Shannon, J. R.; Diedrich, A.; Biaggioni, I.; Tank, J.; Robertson, R. M.; Robertson, D.; Jordan, J. | 2002 | Am J Med | 10.1016/s0002-9343(02)01025-2 | Exclusion reason: Wrong patient population; |
| Is sinus node modification appropriate for inappropriate sinus tachycardia with features of postural orthostatic tachycardia syndrome? | Shen, W. K.; Low, P. A.; Jahangir, A.; Munger, T. M.; Friedman, P. A.; Osborn, M. J.; Stanton, M. S.; Packer, D. L.; Rea, R. F.; Hammill, S. C. | 2001 | Pacing Clin Electrophysiol | 10.1046/j.1460-9592.2001.00217.x | Exclusion reason: Wrong study design; |
| Long-term follow-up of patients with postural tachycardia syndrome | Sousa, A.; Lebreiro, A.; Freitas, J.; Maciel, M. J. | 2012 | Clin Auton Res | 10.1007/s10286-011-0155-1 | Exclusion reason: Wrong study design; |
| Low-Dose Naltrexone Use in Postural Orthostatic Tachycardia Syndrome: A Case Series | Stallkamp Tidd, S. J.; Cantrell, C.; Greene, B. D.; Wilson, R. | 2023 | Cureus | 10.7759/cureus.43426 | Exclusion reason: Wrong study design; |
| Pediatric-Onset Postural Orthostatic Tachycardia Syndrome in a Single Tertiary Care Center | Staples, A.; Thompson, N. R.; Moodley, M. | 2020 | J Child Neurol | 10.1177/0883073820916260 | Exclusion reason: Wrong study design; |
| Intravenous cannulation of adolescents does not affect the modulation of autonomic tone assessed by heart rate and blood pressure variability | Stewart, J. M. | 2000 | Clin Auton Res | 10.1007/bf02291383 | Exclusion reason: Wrong intervention; |
| Clinical and physiological effects of an acute alpha-1 adrenergic agonist and a beta-1 adrenergic antagonist in chronic orthostatic intolerance | Stewart, J. M.; Munoz, J.; Weldon, A. | 2002 | Circulation | 10.1161/01.cir.0000040999.00692.f3 | Exclusion reason: Wrong outcomes; |
| Ascorbate improves circulation in postural tachycardia syndrome | Stewart, J. M.; Ocon, A. J.; Medow, M. S. | 2011 | Am J Physiol Heart Circ Physiol | 10.1152/ajpheart.00018.2011 | Exclusion reason: Wrong outcomes; |
| Pediatric autonomic testing: retrospective review of a large series | Sukul, D.; Chelimsky, T. C.; Chelimsky, G. | 2012 | Clin Pediatr (Phila) | 10.1177/0009922811415102 | Exclusion reason: Wrong study design; |
| Gastrointestinal symptoms associated with orthostatic intolerance | Sullivan, S. D.; Hanauer, J.; Rowe, P. C.; Barron, D. F.; Darbari, A.; Oliva-Hemker, M. | 2005 | J Pediatr Gastroenterol Nutr | 10.1097/01.mpg.0000157914.40088.31 | Exclusion reason: Wrong study design; |
| Long-Term Outcomes of Children and Adolescents With Postural Tachycardia Syndrome After Conventional Treatment | Tao, C.; Lu, W.; Lin, J.; Li, H.; Li, X.; Tang, C.; Du, J.; Jin, H. | 2019 | Front Pediatr | 10.3389/fped.2019.00261 | Exclusion reason: Wrong study design; |
| Reply: Ivabradine in POTS: Increase in Central Pressure Rather Than Down-Regulation of Sympathetic Nervous System | Taub, P. R.; Zadourian, A.; Hsu, J. C. | 2021 | J Am Coll Cardiol | 10.1016/j.jacc.2021.04.046 | Exclusion reason: Wrong study design; |
| Treatment of long COVID complicated by postural orthostatic tachycardia syndrome-Case series research | Tsuchida, T.; Ishibashi, Y.; Inoue, Y.; Ishizuka, K.; Katayama, K.; Hirose, M.; Nakagama, Y.; Kido, Y.; Akashi, Y.; Otsubo, T.; Matsuda, T.; Ohira, Y. | 2024 | J Gen Fam Med | 10.1002/jgf2.670 | Exclusion reason: Wrong study design; |
| [Our experience in the diagnosis and treatment of postural orthostatic tachycardia syndrome, vasovagal syncope, and inappropriate sinus tachycardia in children] | Ugan Atik, S.; Dedeoğlu, R.; Koka, A.; Öztunç, F. | 2017 | Turk Kardiyol Dern Ars | 10.5543/tkda.2017.36517 | Exclusion reason: Wrong language; |
| [Vasovagal syncope or postural orthostatic tachycardia syndrome in children with neurological symptoms at disease onset: a clinical analysis of 88 cases] | Wang, A. P.; Zheng, J.; Wang, C.; Cai, H.; Mao, D. A.; Lin, P.; Li, F.; Luo, H. Y.; Xiong, J. J.; Liu, L. Q. | 2020 | Zhongguo Dang Dai Er Ke Za Zhi | 10.7499/j.issn.1008-8830.1911080 | Exclusion reason: Wrong language; |
| Heart Rate and Heart Rate Difference Predicted the Efficacy of Metoprolol on Postural Tachycardia Syndrome in Children and Adolescents | Wang, S.; Zou, R.; Cai, H.; Wang, Y.; Ding, Y.; Tan, C.; Yang, M.; Li, F.; Wang, C. | 2020 | J Pediatr | 10.1016/j.jpeds.2020.05.017 | Exclusion reason: Wrong outcomes; |
| Baseline Corrected QT Interval Dispersion Is Useful to Predict Effectiveness of Metoprolol on Pediatric Postural Tachycardia Syndrome | Wang, Y.; Sun, Y.; Zhang, Q.; Zhang, C.; Liu, P.; Wang, Y.; Tang, C.; Jin, H.; Du, J. | 2021 | Front Cardiovasc Med | 10.3389/fcvm.2021.808512 | Exclusion reason: Wrong study design; |
| Heart Rate Variability Predicts Therapeutic Response to Metoprolol in Children With Postural Tachycardia Syndrome | Wang, Y.; Zhang, C.; Chen, S.; Liu, P.; Wang, Y.; Tang, C.; Jin, H.; Du, J. | 2019 | Front Neurosci | 10.3389/fnins.2019.01214 | Exclusion reason: Wrong study design; |
| Baseline left ventricular ejection fraction associated with symptom improvements in both children and adolescents with postural tachycardia syndrome under metoprolol therapy | Wang, Y. Y.; Han, Z. H.; Wang, Y. L.; Liao, Y.; Zhang, C. Y.; Liu, P.; Tang, C. S.; Du, J. B.; Jin, H. F.; Huang, Y. Q. | 2021 | Chin Med J (Engl) | 10.1097/cm9.0000000000001698 | Exclusion reason: Wrong study design; |
| Desmopressin in the postural tachycardia syndrome | Weinstock, J. | 2012 | Heart Rhythm | 10.1016/j.hrthm.2012.05.018 | Exclusion reason: Wrong study design; |
| Plasma Exchange Therapy in Postural Tachycardia Syndrome: A Novel Long-Term Approach? | Wells, R.; Hissaria, P.; Elliott, A. D.; Sanders, P.; Page, A.; Baumert, M.; Lau, D. H. | 2020 | Am J Med | 10.1016/j.amjmed.2019.10.016 | Exclusion reason: Wrong study design; |
| Salt supplementation in the management of orthostatic intolerance: Vasovagal syncope and postural orthostatic tachycardia syndrome | Williams, E. L.; Raj, S. R.; Schondorf, R.; Shen, W. K.; Wieling, W.; Claydon, V. E. | 2022 | Auton Neurosci | 10.1016/j.autneu.2021.102906 | Exclusion reason: Wrong study design; |
| Establishment and validation of a multivariate predictive model for the efficacy of oral rehydration salts in children with postural tachycardia syndrome | Xu, B.; Gao, Y.; Zhang, Q.; Li, X.; Liu, X.; Du, J.; Jin, H. | 2024 | EBioMedicine | 10.1016/j.ebiom.2023.104951 | Exclusion reason: Wrong study design; |
| A predictive model of response to metoprolol in children and adolescents with postural tachycardia syndrome | Xu, B. W.; Zhang, Q. Y.; Li, X. Y.; Tang, C. S.; Du, J. B.; Liu, X. Q.; Jin, H. F. | 2023 | World J Pediatr | 10.1007/s12519-022-00677-4 | Exclusion reason: Wrong study design; |
| Short-term efficacy of ORS formulation and propranolol regimen in children with POTS | Yozgat, Y.; Temur, H. O.; Coban, S.; Oner, T.; Karaarslan, U.; Yozgat, C. Y.; Karadeniz, C.; Ergor, S. N.; Erenberk, U. | 2020 | Arch Pediatr | 10.1016/j.arcped.2020.06.001 | Exclusion reason: Wrong outcomes; |
| Poincaré plot can help predict the curative effect of metoprolol for pediatric postural orthostatic tachycardia syndrome | Yuan, P.; Lian, Z.; Wang, Y.; Zhang, C.; Jin, H.; Du, J.; Huang, Y.; Liao, Y. | 2023 | Front Neurosci | 10.3389/fnins.2023.1280172 | Exclusion reason: Wrong outcomes; |
| ORTHOSTATIC HYPOTENSION AND ORTHOSTATIC TACHYCARDIA: NEW CLINICAL OBSERVATIONS, SUCCESSFUL TREATMENT WITH PAREDRINE, AND REVIEW OF LITERATURE | Yuskis, A. S.; Griffith, G. C. | 1948 | Calif Med |  | Exclusion reason: Wrong study design; |
| [Therapies for postural tachycardia syndrome in children] | Zhang, F. W.; Liao, Y.; Li, X. Y.; Chen, L.; Jin, H. F.; Du, J. B. | 2011 | Zhonghua Er Ke Za Zhi |  | Exclusion reason: Wrong language; |
| Orthostatic plasma norepinephrine level as a predictor for therapeutic response to metoprolol in children with postural tachycardia syndrome | Zhang, Q.; Chen, X.; Li, J.; Du, J. | 2014 | J Transl Med | 10.1186/s12967-014-0249-3 | Exclusion reason: Wrong study design; |
| Twenty-four-hour urinary sodium excretion and postural orthostatic tachycardia syndrome | Zhang, Q.; Liao, Y.; Tang, C.; Du, J.; Jin, H. | 2012 | J Pediatr | 10.1016/j.jpeds.2012.01.054 | Exclusion reason: Wrong study design; |
| Plasma copeptin and therapeutic effectiveness of midodrine hydrochloride on postural tachycardia syndrome in children | Zhao, J.; Tang, C.; Jin, H.; Du, J. | 2014 | J Pediatr | 10.1016/j.jpeds.2014.04.032 | Exclusion reason: Wrong study design; |
| Northera Improves Postural Tachycardia Syndrome (POTS) and Postural Vasovagal Syncope (VVS) | NCT02558972, | 2015 |  |  | Exclusion reason: Study not yet finished; |
| Effect of Ivabradine on Patients With Postural Orthostatic Tachycardia Syndrome | NCT03182725, | 2017 |  |  | Exclusion reason: Duplicate; |
| A Shared Medical Appointment Intervention for Quality of Life Improvement in POTS | NCT05454137, | 2022 |  |  | Exclusion reason: Study not yet finished; |
| Intravenous (IV) Saline and Exercise in Postural Tachycardia Syndrome (POTS) | NCT01000350, | 2009 |  |  | Exclusion reason: Study not yet finished; |
| Iron Sucrose in Patients With Iron Deficiency and POTS | NCT04855266, | 2021 |  |  | Exclusion reason: Study withdrawn; |
| Breathing Exercises With And Without Aerobic Training In Patients With Postural Orthostatic Tachycardia Syndrome | NCT05404672, | 2022 |  |  | Exclusion reason: Study not yet finished; |
| The Effect of Ivabradine on Patients With Postural Tachycardia Syndrome | NCT01761825, | 2012 |  |  | Exclusion reason: Study not yet finished; |
| Transdermal Vagal Stimulation for POTS | NCT02281097, | 2014 |  |  | Exclusion reason: Wrong study design; |
| Using Mirabegron to Increase BP in Patients With POTS | NCT06133075, | 2023 |  |  | Exclusion reason: Study not yet finished; |
| The Effect of Physical Activity on Postural Orthostatic Tachycardia Syndrome | NCT05554107, | 2022 |  |  | Exclusion reason: Study not yet finished; |
| Reducing Orthostatic Intolerance With Oral Rehydration in Myalgic Encephalomyelitis/Chronic Fatigue Syndrome Patients | NCT02854683, | 2016 |  |  | Exclusion reason: Study not yet finished; |
| Remote Self-training Program for Patients With Postural Orthostatic Tachycardia Syndrome | NCT04603157, | 2020 |  |  | Exclusion reason: Duplicate; |
| Efficacy and Safety Study of Efgartigimod in Adults With Post-COVID-19 POTS | NCT05633407, | 2022 |  |  | Exclusion reason: Study not yet finished; |
| Non-invasive Vagal Neurostimulation (nVNS) in Adolescents With Postural Orthostatic Tachycardia Syndrome (POTS) | NCT06268288, | 2024 |  |  | Exclusion reason: Study not yet finished; |
| Dietary Sodium's Effect on Urinary Sodium and Dopamine Excretion in Patients With Postural Tachycardia Syndrome | NCT01563107, | 2012 |  |  | Exclusion reason: No publication available; |
| Effect of Exercise in OI | NCT00770484, | 2008 |  |  | Exclusion reason: Duplicate; |
| Vagus Nerve Stimulation in Treatment of Postural Orthostatic Tachycardia Syndrome | NCT03930914, | 2019 |  |  | Exclusion reason: Duplicate; |
| Iron Sucrose in Adolescents With Iron Deficiency and Postural Orthostatic Tachycardia Syndrome (POTS) | NCT01978535, | 2013 |  |  | Exclusion reason: No publication available; |
| Hemodynamic Effects of Compression in POTS | NCT03484273, | 2018 |  |  | Exclusion reason: Duplicate; |
| Autonomic Determinants of POTS - Pilot 2 | NCT04140721, | 2019 |  |  | Exclusion reason: Study not yet finished; |
| High Sodium Diet and External Abdominal Compression in POTS | NCT01771484, | 2012 |  |  | Exclusion reason: Study withdrawn; |
| CAlgary SAlt for POTS | NCT05924646, | 2023 |  |  | Exclusion reason: Study not yet finished; |
| Hemodynamic Response of Neuropathic And Non-Neuropathic POTS Patients To Adrenoreceptor Agonist And Antagonist | NCT03070730, | 2012 |  |  | Exclusion reason: No publication available; |
| Yudanrongxin pills for the treatment of children with postural tachycardia syndrome: a Randomized, Double-blind Pacebo-controlled Trial | ChiCTR-IPR-14005327, | 2014 |  |  | Exclusion reason: No publication available; |
| Semi-supervised exercise training program more efficacious for individuals with postural orthostatic tachycardia syndrome | Wheatley-Guy, CM; Shea, MG; Parks, JK; Scales, R; Goodman, BP; Johnson, BD | 2022 |  | 10.1007/s10286-022-00892-z | Exclusion reason: Conference abstract; |
| Abdominal and Lower Extremity Compression During Tilt Table Testing in Adolescent POTS Patients | NCT01795469, | 2013 |  |  | Exclusion reason: Duplicate; |
| Cardiovascular Effects of Selective I(f)-Channel Blockade | NCT00865917, | 2009 |  |  | Exclusion reason: No publication available; |
| Compression Garments in the Community With POTS | NCT04881318, | 2021 |  |  | Exclusion reason: Study not yet finished; |
| Body compression in postural tachycardia syndrome improves orthostatic tolerance in a dose-dependent pattern | Bourne, KM; Sheldon, RS; Exner, DV; Tyberg, J; Kogut, K; Ng, J; Sheikh, N; Lloyd, M; Scott, E; Raj, SR | 2019 |  | 10.1007/s10286-019-00631-x | Exclusion reason: Conference abstract; |
| BODY COMPRESSION IN POSTURAL TACHYCARDIA SYNDROME: EFFECTS ON ORTHOSTATIC TOLERANCE | Bourne, K; Exner, DV; Mph, MD; Tyberg, JV; Sheldon, RS; Kogut, K; Ng, J; Sheikh, N; Lloyd, M; Scott, E; et al. | 2019 |  | 10.1016/j.hrthm.2019.04.018 | Exclusion reason: Conference abstract; |
| Gabapentin Treatment of Postural Tachycardia Syndrome (PoTS) | NCT04345432, | 2020 |  |  | Exclusion reason: Study not yet finished; |
| Differential effects of dietary salt on blood volume regulation in postural tachycardia syndrome and healthy subjects | Nwazue, VC; Garland, EM; Black, BK; Okamoto, LE; Paranjape, SY; Shibao, CA; Biaggioni, I; Robertson, D; Raj, SR | 2013 |  | 10.1007/s10286-013-0211-0 | Exclusion reason: Conference abstract; |
| Effects of high sodium intake on blood volume and catecholamines in patients with postural tachycardia syndrome and healthy females | Celedonio, JE; Garland, EM; Nwazue, VC; Paranjape, SY; Black, BK; Okamoto, LE; Shibao, CA; Gamboa, A; Biaggioni, I; Robertson, D; et al. | 2014 |  | 10.1007/s10286-014-0251-0 | Exclusion reason: Conference abstract; |
| LB-456640-4 NONINVASIVE VAGUS NERVE STIMULATION IN POSTURAL TACHYCARDIA SYNDROME: a RANDOMIZED CLINICAL TRIAL | Stavrakis, S; Cai, X; Morris, L; Whyte, S; Karfonta, B; Matlock, HG; Asad, ZU; Yu, X | 2023 |  | 10.1016/j.hrthm.2023.04.051 | Exclusion reason: Conference abstract; |
| Efficacy and safety of periodic albumin infusions in refractory postural orthostatic tachycardia syndrome: a comparative study | Siddiqi, Z; Blackmore, D; Soloway, A | 2019 |  | 10.1111/ene.14019 | Exclusion reason: Conference abstract; |
| A Study to Systematically Assess the Efficacy and Safety of Intravenous Albumin Infusions in Severe POTS | NCT03365414, | 2017 |  |  | Exclusion reason: Study withdrawn; |
| Modafinil and Cognitive Function in POTS | NCT01988883, | 2013 |  |  | Exclusion reason: Study not yet finished; |
| Beta blockers and exercise in POTS | Gamboa, A; Okamoto, LE; Arnold, A; Black, B; Diedrich, A; Farley, G; Paranjape, SY; Biaggioni, I | 2010 |  | 10.1007/s10286-010-0082-6 | Exclusion reason: Conference abstract; |
| Combined β-blockade and splanchnic venous compression in the treatment of POTS | Okamoto, LE; Diedrich, A; Gamboa, A; Shibao, C; Black, BK; Raj, SR; Robertson, D; Biaggioni, I | 2015 |  | 10.1016/j.autneu.2015.07.210 | Exclusion reason: Conference abstract; |
| Low Dose Naltrexone Use in Patients With POTS | NCT05363514, | 2022 |  |  | Exclusion reason: Study not yet finished; |
| Effects of Individual Tailored Physical Exercise in Patients With POTS After COVID-19 - a Randomized Controlled Study | NCT05877534, | 2023 |  |  | Exclusion reason: Study not yet finished; |
| Physiology of Long COVID-19 and the Impact of Cardiopulmonary Rehabilitation on Quality-of-Life and Functional Capacity | NCT05566483, | 2022 |  |  | Exclusion reason: Wrong patient population; |
| Assessing the feasibility of a supervised exercise rehabilitation intervention with behavioural and motivational support, for people with postural orthostatic tachycardia syndrome | ISRCTN45323485, | 2020 |  |  | Exclusion reason: Study not yet finished; |
| Protocol update for a randomised controlled feasibility trial of exercise rehabilitation for people with postural tachycardia syndrome: the PULSE study | McGregor, G.; Evans, B.; Sandhu, H.; Simmonds, J.; Joshi, S.; Devi, G.; Zhupaj, A.; Holliday, N.; Pearce, G.; Patel, C.; Hee, S. W.; Powell, R.; Heine, P.; Patel, S.; Kavi, L.; Bruce, J.; Hayat, S.; Lim, B.; Eftekhari, H.; Panikker, S. | 2022 | Pilot Feasibility Stud | 10.1186/s40814-022-01056-6 | Exclusion reason: Study not yet finished; |
| Protocol for a randomised controlled feasibility trial of exercise rehabilitation for people with postural tachycardia syndrome: the PULSE study | McGregor, G.; Hee, S. W.; Eftekhari, H.; Holliday, N.; Pearce, G.; Sandhu, H.; Simmonds, J.; Joshi, S.; Kavi, L.; Bruce, J.; Panikker, S.; Lim, B.; Hayat, S. | 2020 | Pilot Feasibility Stud | 10.1186/s40814-020-00702-1 | Exclusion reason: Study not yet finished; |
| A double-blind, placebo-controlled, crossover pilot trial of gabapentin for treatment of postural tachycardia symptoms | Khurana, RK | 2019 |  | 10.1007/s10286-019-00631-x | Exclusion reason: Conference abstract; |
| Crossover Study of Propranolol vs Ivabradine in POTS | NCT04186286, | 2019 |  |  | Exclusion reason: Study not yet finished; |
| imPROving Quality of LIFe In the Long COVID Patient | NCT05823896, | 2023 |  |  | Exclusion reason: Study not yet finished; |
| Ivabradine for Long-Term Effects of COVID-19 With POTS Cohort | NCT05481177, | 2022 |  |  | Exclusion reason: Study not yet finished; |
| Effect of a neck compression collar on cardiorespiratory function in postural tachycardia syndrome (POTS) | Nardone, M; Guzman, J; Harvey, P; Floras, J; Edgell, H | 2019 |  | 10.1007/s10286-019-00631-x | Exclusion reason: Conference abstract; |
| A Study of Pyridostigmine in Postural Tachycardia Syndrome | NCT00409435, | 2006 |  |  | Exclusion reason: Study not yet finished; |
| Acute modafinil and cognition in postural tachycardia syndrome | Arnold, AC; Haman, K; Garland, EM; Miller, AJ; Wang, M; Shen, B; Paranjape, SY; Black, BK; Diedrich, A; Robertson, D; et al. | 2019 |  | 10.1007/s10286-019-00631-x | Exclusion reason: Conference abstract; |
| Efficacy of compression on different capacitance beds to ameliorate orthostatic symptoms in patients with postural tachycardia syndrome (pots) | Deng, JC; Low, PA; Opfer-Gehrking, TL; FJ, | 2001 |  |  | Exclusion reason: Wrong patient population; |
| Treatment of arterial hypotonia and postural orthostatic tachycardia syndrome. Clinical experience with dihydroergotamine methanesulfonate in 30 cases | García Legido, A; García-Santalla, JM | 1973 |  |  | Exclusion reason: No publication available; |
| Vagal Stimulation in POTS | NCT03124355, | 2017 |  |  | Exclusion reason: Study not yet finished; |
| IVIG (Gamunex-C) Treatment Study for POTS Subjects | NCT03919773, | 2019 |  |  | Exclusion reason: Duplicate; |
| IVABRADINE EFFECTS ON COVID-19 INDUCED POSTURAL ORTHOSTATIC TACHYCARDIA SYNDROME | Abdelnabi, M.; Ahmed, A.; Benjanuwattra, J.; Saleh, Y.; Leelaviwat, N.; Almaghraby, A. | 2023 | Journal of the American College of Cardiology | https://dx.doi.org/10.1016/S0735-1097%2823%2900500-4 | Exclusion reason: Conference abstract; |
| Heart rate and plasma cyclic AMP responses to isoproterenol infusion and effect of beta-adrenergic blockade in patients with postural orthostatic tachycardia syndrome | Abe, H.; Nagatomo, T.; Kohshi, K.; Numata, T.; Kikuchi, K.; Sonoda, S.; Mizuki, T.; Kuroiwa, A.; Nakashima, Y.; Motomura, S.; Toyo-Oka, T.; Hirata, Y. | 2000 | Journal of Cardiovascular Pharmacology | http://dx.doi.org/10.1097/00005344-200000006-00017 | Exclusion reason: Duplicate; |
| Symptom relief from median arcuate ligament syndrome corrective surgery in patients with postural orthostatic tachycardia syndrome and Median arcuate ligament syndrome | Abouelnasr, A.; Alam, S.; Rehman, Z.; Mistry, H.; Munez, K.; Abujajeh, R.; Nasri, M. A.; Khan, M.; Chandralekha, A.; Suleman, A.; Overberg, R. | 2017 | Clinical Autonomic Research | https://dx.doi.org/10.1007/s10286-017-0462-2 | Exclusion reason: Conference abstract; |
| Autonomic parameters in postural orthostatic tachycardia syndrome patients before and after median arcuate ligament syndrome surgery | Alam, S.; Kommera, K.; Mistry, H.; Munez, K.; Algabre, E.; Nasri, M. A.; Joy, N.; Dada, R. D.; Suleman, A. | 2017 | Clinical Autonomic Research | https://dx.doi.org/10.1007/s10286-017-0462-2 | Exclusion reason: Conference abstract; |
| Ovarian vein embolization as treatment for postural orthostatic tachycardia syndrome and pelvic congestion syndrome | Algabre, E. D.; Munez, K.; Kommera, K.; Suleman, A.; Stein, J.; Slonim, S.; Chan, D. | 2018 | Journal of Vascular and Interventional Radiology | https://dx.doi.org/10.1016/j.jvir.2017.12.007 | Exclusion reason: Conference abstract; |
| Intravenous saline provides symptomatic relief for patients with postural orthostatic tachycardia syndrome (POTS) | Almardini, W.; Nasri, M. A.; Mistry, H.; Alam, S. B.; Joy, N.; Munez, K.; Alam, S.; Suleman, A. | 2018 | Cardiology (Switzerland) | https://dx.doi.org/10.1159/000491714 | Exclusion reason: Conference abstract; |
| A NOVEL STRATEGY OF AUTONOMIC REHABILITATION THERAPY FOR POSTURAL ORTHOSTATIC TACHYCARDIA SYNDROME | Almeida, T.; Lemes, C. I.; Hachul, D. T. | 2023 | Heart Rhythm | https://dx.doi.org/10.1016/j.hrthm.2023.03.1427 | Exclusion reason: Conference abstract; |
| ILIAC VEIN STENTING AND QUALITY OF LIFE IN PATIENTS WITH POSTURAL ORTHOSTATIC TACHYCARDIA SYNDROME (POTS) | Almuwaqqat, Z.; Thadani, S.; Bortfeld, K.; McGeoch, C.; Steinberg, R. S.; Garcia, M.; Hassan, M. E.; Cutchins, A. | 2024 | Journal of the American College of Cardiology | https://dx.doi.org/10.1016/S0735-1097%2824%2904303-1 | Exclusion reason: No publication available; |
| Median arcuate ligament syndrome in postural orthostatic tachycardia syndrome (POTS) | Ashangari, C.; Suleman, A.; Le, T. H. | 2015 | Autonomic Neuroscience: Basic and Clinical | https://dx.doi.org/10.1016/j.autneu.2015.07.225 | Exclusion reason: Conference abstract; |
| Hemodynamic consequences of hypocapnic hyperventilation in POTS | Baker, J. R.; Incognito, A. V.; Ranada, S. I.; Phillips, A. A.; Sheldon, R. S.; Wilson, R. J. A.; Raj, S. R. | 2022 | Clinical Autonomic Research | https://dx.doi.org/10.1007/s10286-022-00892-z | Exclusion reason: Conference abstract; |
| PoTS: Long term follow-up of an Italian cohort | Barbieri, F.; Bongiovanni, L. G.; Fondrieschi, L.; Monaco, S. | 2016 | Clinical Neurophysiology | https://dx.doi.org/10.1016/j.clinph.2015.09.056 | Exclusion reason: Conference abstract; |
| Intravenous Immunoglobulin Therapy for Autoimmune Dysautonomia in children: A Case Series | Bolufer, A.; Prakash, V. | 2023 | Neurology | https://dx.doi.org/10.1212/WNL.0000000000202015 | Exclusion reason: Wrong study design; |
| Utilization of medications to reduce the symptoms in pediatric patients with postural orthostatic tachycardia syndrome | Boris, J. R.; Bernadzikowski, T. | 2017 | Clinical Autonomic Research | https://dx.doi.org/10.1007/s10286-017-0462-2 | Exclusion reason: Conference abstract; |
| WAIST-HIGH COMPRESSION GARMENTS REDUCE ORTHOSTATIC TACHYCARDIA IN PATIENTS WITH POSTURAL ORTHOSTATIC TACHYCARDIA SYNDROME IN A COMMUNITY SETTING | Bourne, K.; Karalasingham, K.; Sheldon, R.; Exner, D.; Siddiqui, T.; Hall, J.; Raj, S. | 2022 | Canadian Journal of Cardiology | https://dx.doi.org/10.1016/j.cjca.2022.08.099 | Exclusion reason: Conference abstract; |
| Evaluation of waist-high compression garment use in patients with postural orthostatic tachycardia syndrome in a community setting | Bourne, K. M.; Karalasingham, K.; Sheldon, R. S.; Exner, D. V.; Siddiqui, T.; Hall, J.; Raj, S. R. | 2022 | Clinical Autonomic Research | https://dx.doi.org/10.1007/s10286-022-00892-z | Exclusion reason: Conference abstract; |
| Why do patients wear a compression garment for their POTS symptoms (or not)? | Bourne, K. M.; Sheldon, R. S.; Raj, S. R.; Runte, M. | 2019 | Clinical Autonomic Research | https://dx.doi.org/10.1007/s10286-019-00631-x | Exclusion reason: Conference abstract; |
| Preliminary data on the durability of improved symptoms, functioning, and psychological distress in adolescents with POTS treated in a multidisciplinary treatment program | Bruce, B. K.; Harrison, T. E.; Weiss, K. E.; Fischer, P. R.; Ahrens, S. P.; Timm, W. N. | 2012 | Clinical Autonomic Research | https://dx.doi.org/10.1007/s10286-012-0175-5 | Exclusion reason: Conference abstract; |
| An innovative approach to support women with postural orthostatic tachycardia syndrome (POTS) | Childerhose, D.; Delos-Reyes, F.; Harvey, P.; Landry, M.; Fong, M.; Osuntokun, T.; Price, J. | 2021 | Canadian Journal of Cardiology | https://dx.doi.org/10.1016/j.cjca.2020.02.014 | Exclusion reason: Conference abstract; |
| Median arcuate ligament surgery in postural orthostatic tachycardia syndrome (POTS)-is post operative celiac artery velocity (POCAV) a success marker? | Dada, R. D.; Nasri, M. A.; Mistry, H.; Kommera, K.; Joy, N.; Munez, K.; Alam, S.; Algabre, E.; Iqbal, R.; Suleman, A. | 2017 | Clinical Autonomic Research | https://dx.doi.org/10.1007/s10286-017-0462-2 | Exclusion reason: Conference abstract; |
| Abstract 14305: Long-Term Results of Left Atrial Ganglionated Plexi Ablation in Patients With Syncope and POTS | Das, Mithilesh; Kumar, Awaneesh; Vasilottos, Nektarios | 2022 | Circulation | 10.1161/circ.146.suppl_1.14305 | Exclusion reason: Conference abstract; |
| Long-Term Results of Left Atrial Ganglionated Plexi Ablation in Patients With Syncope and POTS | Das, M.; Kumar, A.; Vasilottos, N. | 2022 | Circulation | https://dx.doi.org/10.1161/circ.146.suppl_1.14305 | Exclusion reason: Conference abstract; |
| Clinical Audit of a Paediatric Neurology Syncope Clinic: A Re-Submission | Davies, G.; Whitehouse, W. P. | 2023 | Developmental Medicine and Child Neurology | https://dx.doi.org/10.1111/dmcn.15477 | Exclusion reason: Conference abstract; |
| Redo procedures after sinus node sparing hybrid ablation for inappropriate sinus tachycardia/postural orthostatic sinus tachycardia | De Asmundis, C.; Marcon, L.; Pannone, L.; Della Rocca, D. G.; Lakkireddy, D.; Beaver, T. M.; Brodt, C. R.; Monaco, C.; Sorgente, A.; Audiat, C.; Vetta, G.; Ramak, R.; Overeinder, I.; Kronenberger, R.; Bala, G.; Almorad, A.; Stroker, E.; Sieira, J.; Sarkozy, A.; Brugada, P.; Chierchia, G. B.; La Meir, M. | 2024 | Europace | https://dx.doi.org/10.1093/europace/euad373 | Exclusion reason: Wrong study design; |
| IVABRADINE IS SUPERIOR TO BETA BLOCKERS IN REDUCING SYMPTOMS IN PEDIATRIC POSTURAL ORTHOSTATIC TACHYCARDIA SYNDROME | Decker, J. A.; Mohammed, M.; Morgan, J. | 2019 | Heart Rhythm | https://dx.doi.org/10.1016/j.hrthm.2019.04.018 | Exclusion reason: Conference abstract; |
| Postural orthostatic tachycardia syndrome: Experience at a single center | Devarapalli, S.; Jayagopal, L. A.; Piccione, E.; Thaisetthawatkul, P. | 2017 | Muscle and Nerve |  | Exclusion reason: No publication available; |
| Transdermal vagal stimulation in postural tachycardia syndrome | Diedrich, A.; Okamoto, L.; Black, B.; Biaggioni, I. | 2018 | Clinical Autonomic Research | https://dx.doi.org/10.1007/s10286-018-0565-4 | Exclusion reason: Conference abstract; |
| Sub-perception transdermal vagal stimulation in postural tachycardia syndrome | Diedrich, A.; Okamoto, L.; Black, B.; Hale, M. D.; Biaggioni, I. | 2018 | Hypertension | https://dx.doi.org/10.1161/hyp.72.suppl_1.P387 | Exclusion reason: Conference abstract; |
| Ivabradine in the paediatric population: Preliminary findings | Donne, G. D.; Roses-Noguer, F.; Till, J.; Salukhe, T.; Prasad, S.; Daubeney, P. | 2016 | Journal of the American College of Cardiology |  | Exclusion reason: Conference abstract; |
| Exercise intolerance in preload failure treated with pyridostigmine | Faria Urbina, M.; Oliveira, R.; Oaklander, A.; Waxman, A. B.; Systrom, D. M. | 2018 | American Journal of Respiratory and Critical Care Medicine |  | Exclusion reason: Conference abstract; |
| The effect of acute volume loading with saline on exercise capacity in postural tachycardia syndrome | Figueroa, R. A.; Arnold, A. C.; Okamoto, L. E.; Diedrich, A.; Paranjape, S. Y.; Black, B. K.; Nwazue, V. C.; Biaggioni, I.; Raj, S. R.; Gamboa, A. | 2013 | Clinical Autonomic Research | https://dx.doi.org/10.1007/s10286-013-0211-0 | Exclusion reason: Conference abstract; |
| Biofeedback for postural orthostatic tachycardia syndrome | Flanigan, Aubrey L. | 2022 | Dissertation Abstracts International: Section B: The Sciences and Engineering |  | Exclusion reason: Wrong study design; |
| A noninvasive neurotechnology, hirrem, is associated with symptom reduction and improved cardiovascular autonomic measures in adolescents with pots | Fortunato, J.; Cook, J.; Lee, S.; Franco, M.; Tegeler, C. | 2014 | Neurology |  | Exclusion reason: Conference abstract; |
| Fludrocortisone acetate improves baroreflex sensitivity and heart rate variability during tilt in children with postural orthostatic tachycardia syndrome | Fortunato, J. E.; Diz, D. I.; Shaltout, H. A. | 2011 | Hypertension | https://dx.doi.org/10.1161/HYP.0b013e318234a201 | Exclusion reason: Conference abstract; |
| Case series using high-resolution, relational, resonancebased, electroencephalic mirroring (HIRREM) for POTS | Fortunato, J. E.; Tegeler, C. L.; Lee, S. W.; Pajewski, N. M.; Franco, M.; Cook, J. F.; Tegeler, C. H. | 2013 | Clinical Autonomic Research | https://dx.doi.org/10.1007/s10286-013-0211-0 | Exclusion reason: Conference abstract; |
| High dose octreotide; a novel therapy for the treatment of drug refractory postural orthostatic tachycardia syndrome in patients with joint hypermobility syndrome | French, A. E.; Shepherd, C.; Horne, A.; Parker, C.; Tagney, J.; Pitts-Crick, J.; Thomas, J. G. | 2011 | Heart | https://dx.doi.org/10.1136/heartjnl-2011-300198.160 | Exclusion reason: Conference abstract; |
| Nonpharmacological treatment of postural orthostatic tachycardia syndrome: Commentary and implications for psychologists | Frye, William S.; Boris, J. R. Moak J. P. Boyle K. Bruce B. K. Harrison T. E. Bee S. M. Luedtke C. A. Porter C. J. Fischer P. R. Hayes S. E. Allman D. A. Ale C. M. Weiss K. E. Bruce B. K. Weiss K. E. Harrison T. E. Allman D. A. Petersen M. A. Luedkte C. A. Fischer P. R. Fedorowski A. Frye W. S. King C. K. Schaefer M. R. Decker J. Kuhn B. Gamboa A. Paranjape S. Y. Black B. K. Arnold A. C. Figueroa R. Okamoto L. E. Nwazue V. C. Diedrich A. Plummer W. D. Dupont W. D. Robertson D. Raj S. R. Goff A. Patel A. Spies J. Chan K. Faulds I. Goodkin M. B. Bellew L. J. Grubb A. F. Grubb B. P. Haker E. Egekvist H. Bjerring P. Hawks J. Pitula C. Shoop J. He W. Wang X. Shi H. Shang H. Li L. Jing X. Zhu B. Junghans-Rutelonis A. N. Craner J. R. Ale C. M. Harbeck-Weber C. Fischer P. R. Weiss K. E. McTate E. A. Weiss K. E. Moss D. Nijjar P. S. Puppala V. K. Dickinson O. Duval S. Duprez D. Kreitzer M. J. Benditt D. G. Ralston T. E. Kanzler K. E. Reilly C. C. Floyd S. V. Lee K. Warwick G. James S. Gall N. Rafferty G. F. Sheldon R. S. Grubb B. P. Olshansky B. Shen W. K. Calkins H. Brignole M. Raj S. R. Krahn A. D. Morillo C. A. Stewart J. M. Sutton R. Sandroni P. Friday K. J. Hachul D. T. Cohen M. I. Lau D. H. Mayuga K. A. Moak J. P. Sandhu R. K. Kanjwal K. | 2024 | Clinical Practice in Pediatric Psychology | https://dx.doi.org/10.1037/cpp0000473 | Exclusion reason: Wrong study design; |
| Chronic treatment with propranolol does not alter sympathetic and cardiovagal baroreflex sensitivity in the Postural Orthostatic Tachycardia Syndrome | Fu, Q.; VanGundy, T. B.; Melyn Galbreath, M.; Shibata, S.; Jarvis, S. S.; Levine, B. D. | 2010 | FASEB Journal |  | Exclusion reason: Conference abstract; |
| Decreased upright heart rate with increased inspiratory resistance in postural tachycardia syndrome | Gamboa, A.; Nwazue, V.; Okamoto, L. E.; Paranjape, S. Y.; Biaggioni, I.; Black, B. K.; Robertson, D.; Raj, S. R. | 2013 | Clinical Autonomic Research | https://dx.doi.org/10.1007/s10286-013-0211-0 | Exclusion reason: Conference abstract; |
| Gravitational Based Therapy for POTS: An International Registry Evaluating the Success of a Structured, Graduated Exercise Program Administered in a Community Setting: Abstract 16542 | George, Stephen; Bivens, Tiffany; Hendrickson, Dianne; Galbreath, Melyn; Fu, Qi; Levine, Benjamin | 2012 | Circulation |  | Exclusion reason: Conference abstract; |
| Gravitational based therapy for pots: An international registry evaluating the success of a structured, graduated exercise program administered in a community setting | George, S. A.; Bivens, T. B.; Hendrickson, D.; Galbreath, M.; Fu, Q.; Levine, B. D. | 2012 | Circulation |  | Exclusion reason: Conference abstract; |
| Menstrual Cycle Variability in Symptoms of Postural Orthostatic Tachycardia Syndrome (POTS) | Goff, A.; O'Sullivan, J.; Nguyen, C.; Wilcox, I. | 2022 | Heart Lung and Circulation | https://dx.doi.org/10.1016/j.hlc.2022.06.352 | Exclusion reason: Conference abstract; |
| CE-541-02 POST-COVID AND POSTURAL ORTHOSTATIC TACHYCARDIA SYNDROME | Haloot, J.; Kabbani, M.; Verduzco-Gutierrez, M.; Bhavaraju-Sanka, R.; Pillarisetti, J. | 2022 | Heart Rhythm | https://dx.doi.org/10.1016/j.hrthm.2022.03.688 | Exclusion reason: Conference abstract; |
| ONE-YEAR LONG-TERM FOLLOW UP FOR POST-ACUTE SEQUELAE OF COVID-19 POTS PATIENTS | Haloot, J.; Shankar, A.; Thunuguntla, S.; Nayak, H. M.; Bhavaraju-Sanka, R.; Verduzco-Gutierrez, M.; Pillarisetti, J. | 2023 | Heart Rhythm | https://dx.doi.org/10.1016/j.hrthm.2023.03.1404 | Exclusion reason: Conference abstract; |
| A transdiagnostic approach to the treatment of autonomic dysfunction associated with orthostatic intolerance in pediatric patients | Hawks, Jessica L.; Pitula, Clio; Shoop, Jamie; Anderson, J. W. Lambert E. A. Sari C. I. Dawood T. Esler M. D. Vaddadi G. Lambert G. W. Armstrong K. R. De Souza A. M. Sneddon P. L. Potts J. E. Claydon V. E. Sanatani S. Bastien C. H. Morin C. M. Ouellet M. C. Blais F. C. Bouchard S. Boris J. R. Bernadzikowski T. Bruce B. K. Harrison T. E. Bee S. M. Eccleston C. Palermo T. M. Williams A. C. Lewandowski H. A. Morley S. Fisher E. Law E. Fedoroff I. C. Taylor S. Freedenberg V. A. Hinds P. S. Friedmann E. Jarjour I. T. Johnson J. N. Mack K. J. Kuntz N. L. Brands C. K. Porter C. J. Fischer P. R. Junghauns-Rutelonis A. N. Postier A. Warmuth A. Schwantes S. Weiss K. E. Kieckhefer G. M. Trahms C. M. Churchill S. S. Kratz L. Uding N. Villareale N. Kizilbash S. J. Ahrens S. P. Bruce B. K. Chelimsky G. Driscoll S. W. Harbeck-Weber C. Lloyd R. M. Mack K. J. Nelson D. E. Ninis N. Pianosi P. T. Stewart J. M. Weiss K. E. Fischer P. R. Kritzberger C. J. Antiel R. M. Wallace D. P. Zacharias J. D. Brands C. K. Fischer P. R. Harbeck-Weber C. Lynch-Jordan A. M. Sil S. Cunningham N. R. Joffe N. Slater S. K. Tran S. T. Crosby L. E. Mayo Clinic McTate E. A. Weiss K. E. Miranda N. A. Boris J. R. Kouvel K. M. Stiles L. Ojha A. Chelimsky T. C. Chelimsky G. Palermo T. M. Raj V. Haman K. L. Raj S. R. Byrne D. Blakely R. D. Biaggioni I. Robertson D. Shelton R. C. Rohde P. Stewart J. M. Boris J. R. Chelimsky G. Fischer P. R. Fortunato J. E. Grubb B. P. Heyer G. L. Jarjour I. T. Medow M. S. Numan M. T. Pianosi P. T. Singer W. Tarbell S. Chelimsky T. C. The Pediatric Writing Group of the American Autonomic Society Twohy E. Malmberg J. Williams J. Vinall J. Pavlova M. Asmundson G. J. G. Rasic N. Noel M. Wetherell J. L. Afari N. Rutledge T. Sorrell J. T. Stoddard J. A. Petkus A. J. Solomon B. C. Lehman D. H. Liu L. Atkinson J. H.; et al., | 2021 | the Behavior Therapist |  | Exclusion reason: No publication available; |
| Clinical, manometric, and radiographic evidence of diffuse gastrointestinal dysmotility in the postural orthostatic tachycardia syndrome (POTS) | Huang, R. J.; Chun, C.; Friday, K.; Triadafilopoulos, G. | 2013 | Gastroenterology |  | Exclusion reason: No publication available; |
| NAUSEA AND VOMITING SEVERITY IS ASSOCIATED WITH RESPONSE TO GASTRIC ELECTRICAL STIMULATION IN CHILDREN | Hurwitz, N. R.; Orsagh-Yentis, D.; Ryan, K. K.; Diefenbach, K. A.; Bali, N.; Vaz, K.; Yacob, D.; Di Lorenzo, C.; Lu, P. L. | 2020 | Gastroenterology | https://dx.doi.org/10.1016/S0016-5085%2820%2933560-5 | Exclusion reason: Wrong patient population; |
| The effects of the somatostatin analogue, octreotide, on orthostasis before and after food ingestion, in the postural tachycardia syndrome | Iodice, V.; Low, D.; Mathias, C. | 2012 | European Journal of Neurology | https://dx.doi.org/10.1111/j.1468-1331.2012.03887.x | Exclusion reason: Conference abstract; |
| The effects of the somatostatin analogue, octreotide, on orthostasis before and after food ingestion, in the Postural Tachycardia Syndrome | Iodice, V.; Low, D. A.; Mathias, C. J. | 2011 | Clinical Autonomic Research | https://dx.doi.org/10.1007/s10286-011-0137-3 | Exclusion reason: Conference abstract; |
| CONTRASTING EFFECTS OF VOLUME LOADING AND [alpha]-ADRENERGIC AGONISTS IN POSTURAL ORTHOSTATIC TACHYCARDIA SYNDROME | Jordan, J.; Shannon, J.; Barwise, J.; Black, B.; Robertson, D. | 1998 | Med Sci Sports Exerc |  | Exclusion reason: No publication available; |
| Depression and Quality of Life in Patients with Postural Orthostatic Tachycardia Syndrome: How Does Cardiac Rehab Participation Factor in? | Jordan, M. | 2023 | Journal of Cardiopulmonary Rehabilitation and Prevention | https://dx.doi.org/10.1097/HCR.0000000000000819 | Exclusion reason: Conference abstract; |
| Droxidopa management in postural orthostatic tachycardia syndrome (POTS) patients | Joy, N.; Algabre, E.; Mistry, H.; Munez, K.; Nasri, M. A.; Alam, S.; Jajunda, R.; Kommera, K.; Rehman, Z.; Suleman, A. | 2017 | Clinical Autonomic Research | https://dx.doi.org/10.1007/s10286-017-0462-2 | Exclusion reason: Conference abstract; |
| Exercise Training in Postural Orthostatic Tachycardia Syndrome: Blocking the Urge to Block [beta]-Receptors? | Joyner, Michael | 2011 | Hypertension | 10.1161/HYPERTENSIONAHA.111.173872 | Exclusion reason: Wrong study design; |
| Effectiveness of droxidopa in children with postural orthostatic tachycardia syndrome | Kakavand, B.; Pires, K. | 2017 | Clinical Autonomic Research | https://dx.doi.org/10.1007/s10286-017-0462-2 | Exclusion reason: Conference abstract; |
| Median Arcuate Ligament Syndrome and Concomitant Autonomic Dysfunction Pathology-Case Reports and Penn Treatment Algorithm | Kalapatapu, V. | 2022 | Journal of Vascular Surgery | https://dx.doi.org/10.1016/j.jvs.2022.03.379 | Exclusion reason: Conference abstract; |
| Medical treatment of postural orthostatic tachycardia syndrome (POTS)-interim analyses | Kim, T. J.; Moon, J. S.; Kim, D. Y.; Jung, K. H.; Lee, S. T.; Sunwoo, J. S.; Byun, J. I.; Lim, J. A.; Jung, K. Y.; Lee, S. K.; Chu, K. | 2015 | Annals of Neurology | https://dx.doi.org/10.1002/ana.24498 | Exclusion reason: Conference abstract; |
| Efficacy of epidural blood patch as a treatment for chronic orthostatic intolerance: A case series | Kinsella, L.; Boedefeld, M.; Stephens, J. | 2019 | Clinical Autonomic Research | https://dx.doi.org/10.1007/s10286-019-00631-x | Exclusion reason: Conference abstract; |
| Cerebral blood flow, autoregulation, and symptoms during orthostasis with lower body compression in postural tachycardia syndrome | Lloyd, M. G.; Bourne, K. M.; Sheldon, R. S.; Exner, D. V.; Tyberg, J.; Kogut, K.; Ng, J.; Sheikh, N.; Scott, E.; Raj, S. R. | 2019 | Clinical Autonomic Research | https://dx.doi.org/10.1007/s10286-019-00631-x | Exclusion reason: Conference abstract; |
| A study in syncope: A review of 94 tilt table tests | Monaghan, M.; McCarron, M.; Purvis, J. | 2013 | Irish Journal of Medical Science | https://dx.doi.org/10.1007/s11845-013-1006-y | Exclusion reason: Conference abstract; |
| SEVERE POTS (POSTURAL ORTHOSTATIC TACHYCARDIA SYNDROME) | Nair, P.; Gupta, S.; Peshimam, N.; Sesham, R. | 2022 | Archives of Disease in Childhood | https://dx.doi.org/10.1136/archdischild-2022-rcpch.690 | Exclusion reason: Conference abstract; |
| Outcome of adolescent onset pots (Postural Orthostatic Tachycardia Syndrome) | Nair, P.; MacCarthy, T.; Osman, N. E. M. | 2021 | Archives of Disease in Childhood | https://dx.doi.org/10.1136/archdischild-2021-rcpch.668 | Exclusion reason: Conference abstract; |
| Effects of ivabradine on autonomic parameters in postural orthostatic tachycardia syndrome (POTS) patients | Nasri, M. A.; Alam, S.; Kommera, K.; Mistry, H.; Munez, K.; Joy, N.; Algabre, E.; Iqbal, R.; Dada, R. D.; Suleman, A. | 2017 | Clinical Autonomic Research | https://dx.doi.org/10.1007/s10286-017-0462-2 | Exclusion reason: Conference abstract; |
| Immunotherapy With Subcutaneous Immunoglobulin or Plasmapheresis in Patients With Postural Orthostatic Tachycardia Syndrome (POTS) | Nelson, R.; Kesterson, K.; Schofield, J.; Blitshteyn, S. | 2022 | Neurology | https://dx.doi.org/10.1212/01.wnl.0000903616.08384.e6 | Exclusion reason: Wrong study design; |
| Combined beta-blockade and splanchnic venous compression in the treatment of POTS | Okamoto, L. E.; Diedrich, A.; Gamboa, A.; Shibao, C.; Black, B. K.; Raj, S. R.; Robertson, D.; Biaggioni, I. | 2015 | Autonomic Neuroscience: Basic and Clinical | https://dx.doi.org/10.1016/j.autneu.2015.07.210 | Exclusion reason: Conference abstract; |
| O-AD003. Postural Orthostatic Tachycardia Syndrome (POTS): A case series from India | Parthasarathy, B.; Grace Roy, A. | 2021 | Clinical Neurophysiology | https://dx.doi.org/10.1016/j.clinph.2021.02.138 | Exclusion reason: Conference abstract; |
| Postural orthostatic tachycardia: Study of eight patients | Perez, S. S.; Gila, T. F. | 1998 | Medicina Clinica |  | Exclusion reason: Wrong language; |
| Experience and results for laparoscopic median arcuate ligament release in young patients with postural orthostatic tachycardia syndrome | Petrosyan, M.; Franklin, A.; Guzzetta, P.; Abdullah, H.; Kane, T. D. | 2015 | Gastroenterology |  | Exclusion reason: Conference abstract; |
| Abstract 16180: Blockade of Norepinephrine Transporter Magnifies Tachycardia and Worsens Symptoms in Postural Tachycardia Syndrome | Raj, Satish; Biaggioni, Italo; Black, Bonnie; Shibao, Cyndya; Robertson, David; Paranjape, Sachin | 2010 | Circulation |  | Exclusion reason: Conference abstract; |
| Modafinil increases blood pressure, but not heart rate, and does not worsen symptoms in postural tachycardia syndrome (POTS) | Raj, S. R.; Kpaeyeh Jr, J. A. G.; Biaggioni, I.; Black, B. K.; Shibao, C.; Robertson, D. | 2013 | Heart Rhythm |  | Exclusion reason: No publication available; |
| The effect of dietary sodium on blood volume and heart rate in postural tachycardia syndrome | Raj, S. R.; Nwazue, V.; Garland, E.; Black, B.; Okamoto, L.; Paranjape, S.; Shibao, C.; Biaggioni, I.; Robertson, D. | 2014 | Heart Rhythm | https://dx.doi.org/10.1016/j.hrthm.2014.03.025 | Exclusion reason: Conference abstract; |
| Inspiratory resistance decreases upright heart rate in postural tachycardia syndrome | Raj, S. R.; Nwazue, V.; Okamoto, L.; Paranjape, S.; Biaggioni, I.; Black, B. K.; Robertson, D.; Gamboa, A. | 2013 | Heart Rhythm |  | Exclusion reason: Duplicate; |
| Prevalence and treatment of small intestinal bacterial overgrowth (SIBO) in patients with postural orthostatic tachycardia syndrome (POTS) | Rehman, Z.; Rajumon, M.; Alam, S. B.; Almardini, W.; Mistry, H.; Khan, A.; Noor, N.; Gaied, L. B.; Alam, S.; Sheikh, B.; Nasri, M. A.; Suleman, A. | 2018 | Clinical Autonomic Research | https://dx.doi.org/10.1007/s10286-018-0565-4 | Exclusion reason: Conference abstract; |
| POTS & PANTS: Improving cerebral perfusion using lower body compression in patients with postural tachycardia syndrome (POTS) is independent of heart rate | Reiter-Campeau, S.; Schondorf, N.; Benoit, J.; Schondorf, R. | 2022 | Clinical Autonomic Research | https://dx.doi.org/10.1007/s10286-022-00892-z | Exclusion reason: Conference abstract; |
| Long lasting changes in symptoms and autonomic profile after 14-day tVNS in hyperadrenergic postural orthostatic tachycardia syndrome (POTS) | Rigo, S.; Shiffer, D.; Minonzio, M.; Pellizon, F.; Bisoglio, A.; Mehrez, D.; Zamuner, A. R.; Porta, A.; Cairo, B.; Tobaldini, E.; Furlan, L.; Montano, N.; Vasile, U.; Biaggioni, I.; Diedrich, A.; Furlan, R. | 2022 | Clinical Autonomic Research | https://dx.doi.org/10.1007/s10286-022-00892-z | Exclusion reason: Conference abstract; |
| Urologic symptoms in pediatric patients with postural orthostatic tachycardia syndrome | Rivera, M. E.; Kimmes, S. A.; Strand, A. B.; Fischer, P. R.; Granberg, C. F. | 2014 | Clinical Autonomic Research | https://dx.doi.org/10.1007/s10286-014-0251-0 | Exclusion reason: Conference abstract; |
| Postural Tachycardia Syndrome (POTS) in Migraine Patients & their Response to Erenumab | Robblee, J.; Vanderpluym, J.; Mendez, N.; Potter, J.; Slonaker, J.; Grimsrud, K.; Starling, A. J. | 2019 | Cephalalgia | https://dx.doi.org/10.1177/0333102419859835 | Exclusion reason: Conference abstract; |
| Functional restoration as an adjunct to pharmacotherapy in treatment of postural orthostatic tachycardia syndrome (POTS) | Rummans, T.; Bruce, B.; Harrison, T. | 2012 | International Journal of Neuropsychopharmacology | https://dx.doi.org/10.1017/S1461145712000508 | Exclusion reason: Conference abstract; |
| Managing gastrointestinal manifestations in patients with postural orthostatic tachycardia syndrome-a UK district general hospital experience | Sagar, V.; Nayagam, J.; Asante, M. | 2018 | United European Gastroenterology Journal | https://dx.doi.org/10.1177/2050640618792819 | Exclusion reason: Conference abstract; |
| Autoimmunity and immunotherapy in postural tachycardia syndrome | Sato, K.; Yamaga, A.; Nishimura, Y.; Shibata, K.; Nakaoka, T.; Sunami, Y.; Hirai, T.; Nakane, T.; Sakura, H. | 2019 | Clinical Autonomic Research | https://dx.doi.org/10.1007/s10286-019-00631-x | Exclusion reason: Conference abstract; |
| Comparative Cohort Study of Post-Acute Covid-19 Infection with a Nested, Randomized Controlled Trial of Ivabradine for Those With Postural Orthostatic Tachycardia Syndrome (The COVIVA Study) | Saunders, D.; Arnold, T. B.; Lavender, J. M.; Bi, D.; Alcover, K.; Hellwig, L. D.; Leazer, S. T.; Mohammed, R.; Markos, B.; Perera, K.; Shaw, D.; Kobi, P.; Evans, M.; Mains, A.; Tanofsky-Kraff, M.; Goguet, E.; Mitre, E.; Pratt, K. P.; Dalgard, C. L.; Haigney, M. C. | 2023 | medRxiv | https://dx.doi.org/10.1101/2023.04.25.23289110 | Exclusion reason: Study not yet finished; |
| Pharmacological I(f) pacemaker current inhibition in a human postural tachycardia syndrome (POTS) model | Schroeder, C.; Heusser, K.; Rieck, D.; Luft, F. C.; Tank, J.; Jordan, J. | 2012 | Clinical Autonomic Research | https://dx.doi.org/10.1007/s10286-012-0175-5 | Exclusion reason: Conference abstract; |
| Abstract 302: Negative Effects of Fludrocortisone Treatment in Adolescents with POTS and Syncope | Shaltout, Hossam; Fortunato, John; Diz, Debra | 2012 | Hypertension |  | Exclusion reason: Conference abstract; |
| Effect of fludrocortisone acetate on nausea and autonomic function during tilt in children with postural orthostatic tachycardia syndrome | Shaltout, H. A.; Diz, D. I.; Fortunato, J. E. | 2011 | Clinical Autonomic Research | https://dx.doi.org/10.1007/s10286-011-0137-3 | Exclusion reason: Conference abstract; |
| Negative effects of fludrocortisone treatment in adolescents with pots and syncope | Shaltout, H. A.; Fortunato, J. E.; Diz, D. I. | 2012 | Hypertension |  | Exclusion reason: Conference abstract; |
| Effects of transcutaneous vagal nerve stimulation on orthostatic tolerance in patients with postural tachycardia syndrome (POTS) | Shiffer, D.; Furlan, R.; Barbic, F.; Minonzio, M.; Cairo, B.; Porta, A.; Montano, N.; Tobaldini, E.; Furlan, L.; Urechie, V.; Biaggioni, I.; Diedrich, A. | 2019 | Clinical Autonomic Research | https://dx.doi.org/10.1007/s10286-019-00631-x | Exclusion reason: Conference abstract; |
| Autonomic profiles and symptoms of hyperadrenergic versus nonhyperadrenergic POTS patients and their response to chronic tVNS | Shiffer, D.; Rigo, S.; Minonzio, M.; Mehrez, D.; Pellizon, F.; Bisoglio, A.; Zamuner, A. R.; Porta, A.; Tobaldini, E.; Furlan, L.; Montano, N.; Vasile, U.; Biaggioni, I.; Diedrich, A.; Furlan, R. | 2022 | Clinical Autonomic Research | https://dx.doi.org/10.1007/s10286-022-00892-z | Exclusion reason: Conference abstract; |
| Efficacy and safety of periodic albumin infusions in refractory postural orthostatic tachycardia syndrome: A comparative study | Siddiqi, Z. A.; Blackmore, D.; Soloway, A. | 2019 | Canadian Journal of Neurological Sciences | https://dx.doi.org/10.1017/cjn.2019.140 | Exclusion reason: Conference abstract; |
| Refractory postural orthostatic tachycardia syndrome: Efficacy and safety of weekly albumin infusions | Siddiqi, Z. A.; Soloway, A.; Blackmore, D. | 2016 | Journal of Neuromuscular Diseases | https://dx.doi.org/10.3233/JND-160001 | Exclusion reason: Conference abstract; |
| Enhanced external counterpulsation (ECP) use in postural orthostatic tachycardia syndrome (POTS): Case series | Siddiqui, S.; Ajmal, J.; Suleman, A. | 2013 | Clinical Autonomic Research | https://dx.doi.org/10.1007/s10286-013-0211-0 | Exclusion reason: Conference abstract; |
| Propranolol decreases tachycardia but does not improve postural stability and cerebral blood flow in pots | Su, C. W.; Chiu, C. C.; Yeh, S. J. | 2014 | Cerebrovascular Diseases | https://dx.doi.org/10.1159/000367674 | Exclusion reason: Conference abstract; |
| Postural Orthostatic Tachycardia Syndrome After COVID-19 Vaccination | Teodorescu, D. L.; Kote, A.; Reaso, J.; Rosenberg, C.; Liu, X.; Kwan, A. C.; Cheng, S.; Chen, P. S. | 2023 | Circulation | https://dx.doi.org/10.1161/circ.148.suppl_1.15090 | Exclusion reason: Wrong outcomes; |
| PatientsWith Postural Orthostatic Tachycardia SyndromeWho Have Psychiatric Disorders Experience Increased Treatment Failures | Tidd, S.; Nowacki, A.; Singh, T.; Wilson, R. | 2023 | Neurology | https://dx.doi.org/10.1212/WNL.0000000000203088 | Exclusion reason: Wrong study design; |
| Abnormal Blood Pressure Fall During Hyperventilation Maneuver In Postural Tachycardia Syndrome | Urechie, V.; Rigo, S.; Shibao, C. A.; Okamoto, L. E.; Gamboa, A.; Biaggioni, I.; Wahba, A.; Elkholey, K.; Giesecke, M.; Muldowney, J.; Mohr, A. E.; Diedrich, A. | 2022 | Hypertension | https://dx.doi.org/10.1161/hyp.79.suppl_1.P131 | Exclusion reason: Conference abstract; |
| Abnormal blood pressure fall during hyperventilation maneuver in postural tachycardia syndrome | Urechie, V.; Rigo, S.; Shibao, C. A.; Okamoto, L. E.; Gamboa, A.; Biaggioni, I.; Wahba, A.; Elkholey, K.; Giesecke, M.; Muldowney, J. A. S.; Mohr, A.; Diedrich, A. | 2022 | Clinical Autonomic Research | https://dx.doi.org/10.1007/s10286-022-00892-z | Exclusion reason: Conference abstract; |
| Open Label use of carbidopa for treatment of hyperadrenergic symptoms | Vernino, S. | 2014 | Clinical Autonomic Research | https://dx.doi.org/10.1007/s10286-014-0251-0 | Exclusion reason: Conference abstract; |
| EP099 / #275 PERCUTANEOUS AURICULAR VAGUS NERVE STIMULATION IN POST-COVID-19 SYNDROME: A CASE SERIES: EPOSTER VIEWING: AS13 - NON- AND LESS-INVASIVE BRAIN STIMULATION | Wolf, A.; Wolf, B.; Stremnitzer, C.; Kampusch, S. | 2023 | Neuromodulation | https://dx.doi.org/10.1016/j.neurom.2023.10.107 | Exclusion reason: Conference abstract; |
| Efficacy of serotonin reuptake inhibitors in postural orthostatic tachycardia syndrome | Yamada, U.; Ohta, D. | 2014 | Psychosomatic Medicine | https://dx.doi.org/10.1097/PSY.0000000000000057 | Exclusion reason: Conference abstract; |
| The follow-up study on the treatment of children with postural orthostatic Tachycardia syndrome | Yang, J.; Liao, Y.; Zhang, F.; Chen, L.; Du, J.; Jin, H. | 2014 | Annals of Pediatric Cardiology |  | Exclusion reason: Wrong language; |
| Children suffering from postural orthostatic tachycardia syndrome with a marked increase in erythrocytic hydrogen sulfide have a better therapeutic response to midodrine hydrochloride | Yang, J.; Zhao, J.; Liu, D.; Fu, C.; Li, X.; Chen, S.; Zhang, F.; Tang, C.; Du, J.; Jin, H. | 2012 | Nitric Oxide - Biology and Chemistry | https://dx.doi.org/10.1016/j.niox.2012.08.030 | Exclusion reason: Conference abstract; |
| Propranolol decreases tachycardia but does not improve orthostatic reduction of cerebral blood flow in the postural orthostatic tachycardia syndrome | Yeh, S. J.; Su, C. W.; Chiu, C. C. | 2014 | Clinical Autonomic Research | https://dx.doi.org/10.1007/s10286-014-0251-0 | Exclusion reason: Conference abstract; |
| 3 years experience of diagnosing and managing postural tachycardia (PoTS) from a UK regional syncope service | Zheng, Y.; Moyles, C.; Blackburn, Y.; Joy, E.; Mohee, K.; Morley, C. | 2015 | European Heart Journal | https://dx.doi.org/10.1093/eurheartj/ehv400 | Exclusion reason: Conference abstract; |
| Open Label Extension of Efgartigimod in Adults With Post-COVID-19 POTS |  |  |  |  | Exclusion reason: Study not yet finished; |
| A Novel Noninvasive Thermoregulatory Device for Postural Tachycardia Syndrome |  |  |  |  | Exclusion reason: Study not yet finished; |
| NC Testing in LC & POTS |  |  |  |  | Exclusion reason: Study not yet finished; |
| Long-term Effects of Transcutaneous Vagal Nerve Stimulation on Postural Orthostatic Tachycardia Syndrome (POTS) |  |  |  |  | Exclusion reason: Study not yet finished; |
| Alterations of Attention in POTS Depending on Body Position and Hydration |  |  |  |  | Exclusion reason: Duplicate; |
| Physical Training in Patients With POTS After Covid-19 |  |  |  |  | Exclusion reason: Study not yet finished; |
| Aldosterone & Sodium Regulation in Postural Tachycardia Syndrome - Screening |  |  |  |  | Exclusion reason: Wrong outcomes; |
| Anti-Cholinergic Receptors Antibodies, Autonomic Profile and Dysautonomia Symptoms in PAF, ALS and POTS (DISAUT-AB) |  |  |  |  | Exclusion reason: Study not yet finished; |
| Acute Salt Handling in Orthostatic Intolerance |  |  |  |  | Exclusion reason: Study not yet finished; |
| Physical Activity as a Complementary Treatment in POTS |  |  |  |  | Exclusion reason: Study withdrawn; |
| Cardiovascular Autonomic and Immune Mechanism of Post COVID-19 Tachycardia Syndrome |  |  |  |  | Exclusion reason: Study not yet finished; |
| Implementation of Transdx Group for POTS |  |  |  |  | Exclusion reason: Study not yet finished; |
|  |  |  |  |  |  |
| Systematic Literature Review - Treatment of Postural Orthostatic Tachycardia Syndrome (POTS; Clinical Autonomic Research; Authors: Nicole Schiweck, Katharina Langer, Andrea Maier, Daniel Vilser, Juliane Spiegler; University Hospital Wuerzburg; Corresponding author: Juliane Spiegler (spiegler_j@ukw.de) | | | | | |
