# Supplementary material for: Systematic literature review: treatment of postural orthostatic tachycardia syndrome (POTS)
Source: Clin Auton Res. 2025 Nov 12;36(1):3–16. doi: 10.1007/s10286-025-01172-2 (PMC12982215; doi:10.1007/s10286-025-01172-2)
Supplement: Supplementary file 7 — Supplementary file7 (DOCX 45 kb) [file 10286_2025_1172_MOESM7_ESM.docx]

**Supplement 7 - SUMMARY OF FINDINGS (RCT)**

**Summary of findings – Pharmacological interventions compared to Placebo or Standard-of-Care in patients with POTS (RCT)**

**Pharmacological interventions compared to Placebo or Standard-of-Care in Patients with POTS (RCT studies)**

**Population:** Adults and / or children with a confirmed diagnosis of POTS

**Intervention:** Pharmacological interventions (see below)

**Comparison:** Placebo or Standard-of-Care

| Studies | Intervention (+ Dosage) | Outcome | Number of participants included in the analysis  (studies) | Age of participants  (MEAN + SD or Age Range) | Sex Ratio (female : male; percentage of females) | Country | Certainty of evidence (GRADE) | Comments |
| --- | --- | --- | --- | --- | --- | --- | --- | --- |
| Green et al. (2013) | **Atomoxetine**  **40 mg (single dose)** | Change in Heart Rate upon postural change (bpm) | 27 participants (1 RCT study) | 34 ± 9 years | 25 : 2  (93 %) | United States (US) | Very low (ACIP 4) | In adult patients, four hours after the application of 40 mg atomoxetine, there was no decrease in heart rate increment upon postural change vs. placebo. Due to a moderate risk of bias and a small size of population, this study´s level of certainty is very low. A positive effect of Atomoxetine on hemodynamics cannot be ruled out. There was no specific data on the use of Atomoxetine in ME/CFS. |
| Green et al. (2013) |  | Symptom Burden (Vanderbilt Orthostatic Symptom Scale) | 27 participants (1 RCT study) | 34 ± 9 years | 25 : 2 (93 %) | United States (US) | Very low (ACIP 4) | In adult patients, four hours after the application of 40 mg atomoxetine, there was no significant decrease in symptom burden vs. placebo . Due to a moderate risk of bias and a small size of population, this study´s level of certainty is very low. A positive effect of Atomoxetine on symptom burden cannot be ruled out. There was no specific data on the use of Atomoxetine in ME/CFS. |
| Coffin et al. (2012) | Desmopressin (DDAVP)  0.2 mg (single dose, oral) | Change in Heart Rate upon postural change (bpm) | 30 participants (1 RCT study) | 37 ± 11 years | 26 : 4 (87 %) | United States (US) | Very low (ACIP 4) | In adult patients, four hours after a single application of 0.2 mg Desmopressine, there was a non-significant decrease in the change in heart rate upon postural change vs. placebo (p = 0.181). Due to a moderate risk of bias and a small size of population, this study´s level of certainty is very low A positive effect of Desmopressin on hemodynamics cannot be ruled out. There was no specific data on the use of Desmopressin in ME/CFS. |
| Coffin et al. (2012) |  | Symptom Burden (Vanderbilt Orthostatic Symptom Scale) | 30 participants (1 RCT study) | 37 ± 11 years | 26 : 4 (87 %) | United States (US) | Very low (ACIP 4) | In adult patients, four hours after the application of 0.2 mg Desmopressin, there was a significant decrease in symptom burden vs. placebo (p = 0.009). Due to a moderate risk of bias and a small size of population, this study´s level of certainty is very low. Desmopressin might have an effect on symptom burden in POTS patients. There was no specific data on the use of Desmopressin in ME/CFS. |
| Vernino et al. (2024) | Intravenous Immunoglobulines (IVIG)  0.4 gm/kg | Symptom Burden (Vanderbilt Orthostatic Symptom Scale) | 28 participants (1 RCT study) | 18 – 55 years | not clearly presented | United States (US) | Very low (ACIP 4) | In adult patients, 12 weeks after the application of intravenous immunoglobulines (IVIG), there was no significant decrease in symptom burden vs. albumin application. Due to a moderate risk of bias and a small size of population, this study´s level of certainty is very low. A positive effect of IVIG on symptom burden cannot be ruled out. There was no specific data on the use of IVIG in ME/CFS. |
| Vernino et al. (2024) |  | Symptom Burden (COMPASS-31) | 28 participants (1 RCT study) | 18 – 55 years | not clearly presented | United States (US) | Very low (ACIP 4) | In adult patients, 12 weeks after the application of intravenous immunoglobulines (IVIG), there was no significant decrease in symptom burden vs. albumin application . Due to a moderate risk of bias and a small size of population, this study´s level of certainty is very low. A positive effect of IVIG on symptom burden cannot be ruled out. There was no specific data on the use of IVIG in ME/CFS. |
| Taub et al. (2021) | Ivabradine  5mg (2x / d)  [10 mg / d] | Change in Heart Rate upon postural change (bpm) | 22 participants (1 RCT study) | 33.9 ± 11.7 years | not clearly presented | United States (US) | Very low (ACIP 4) | In adult patients, after one month of undergoing Ivabradine therapy, there was a significant decrease in the change in heart rate following HUT vs. placebo (p < 0.001). Due to the risk of imprecision including a small size of population, this study´s level of certainty is very low. Ivabradine might have an effect on HR increment in POTS patients. There was no specific data on the use of Ivabradine in ME/CFS. |
| Taub et al. (2021) |  | Quality of Life (SF-36) | 22 participants (1 RCT study) | 33.9 ± 11.7 years | not clearly presented | United States (US) | Very low (ACIP 4) | In adult patients, after one month of undergoing Ivabradine therapy, there was a significant decrease in physical functioning (p 0.008) and social functioning (p 0.021) vs. placebo. Due to the risk of imprecision, including a small size of population, this study´s level of certainty is very low. Ivabradine might improve quality of life in POTS patients. There was no specific data on the use of Ivabradine in ME/CFS. |
| Green et al. (2014) | Melatonin  3 mg (single dose) | Upright Heart Rate (bpm) | 78 participants (1 RCT study) | 32 ± 9 years | 72 : 6 (92 %) | United States (US) | Very low (ACIP 4) | In adult patients, four hours after the application of melatonin, there was a significant decrease in upright HR vs. placebo. Due to a moderate risk of bias and a small size of population, and a high risk of publication bias, this study´s level of certainty is very low. Melatonin might have a positive effect on HR increment in POTS patients. There was no specific data on the use of Melatonin in ME/CFS. |
| Green et al. (2014) |  | Symptom Burden (Vanderbilt Orthostatic Symptom Scale) | 78 participants (1 RCT study) | 32 ± 9 years | 72 : 6 (92 %) | United States (US) | Very low (ACIP 4) | In adult patients, four hours after the application of melatonin, there was no significant change in symptom burden vs. placebo. Due to a moderate risk of bias and a small size of population, and a high risk of publication bias, this study´s level of certainty is very low. A positive effect on symptom burden in POTS cannot be ruled out. There was no specific data on the use of Melatonin in ME/CFS. |
| Ross et al. (2014) | Midodrine  2.5 – 10 mg 3x/d  [max. 30 mg / d] | Upright Heart Rate (bpm) | 20 participants (1 RCT study) | 12 – 20 years | 15 : 5 (75 %) | United States (US) | Very low (ACIP 4) | In teenage patients with neuropathic POTS, a therapy with Midodrine over 2 weeks resulted in a significant decrease in heart rate upon tilt. In hyperadrenergic POTS, there was no significant decrease in HR (p = 0.3). Due to a very small population size and a moderate risk of bias, this study´s certainty is very low. In daily clinical practice, a differentiation between subtypes of POTS is not usual. Midodrine might have a positive effect on upright heart rate in POTS patients. There was no specific data on the use of Midodrine in ME/CFS. |
| Kpaeyeh et. al (2014) | Modafinil  100 mg | Upright Heart Rate (bpm) | 54 participants (1 RCT study) | 32 ± 10 years | 48 : 6 (89 %) | United States (US) | Very low (ACIP 4) | In adult patients, four hours after the application of 100 mg modafinil, there was a non-significant decrease in the change in upright heart rate vs. placebo . Due to a moderate risk of bias and a small size of population, this study´s level of certainty is very low. A positive effect on hemodynamics in POTS cannot be ruled out. There was no specific data on the use of Modafinil in ME/CFS. |
| Kpaeyeh et. al (2014) |  | Symptom Burden (Vanderbilt Orthostatic Symptom Scale) | 54 participants (1 RCT study) | 32 ± 10 years | 48 : 6 (89 %) | United States (US) | Very low (ACIP 4) | In adult patients, four hours after the application of 100 mg modafinil, there was no significant decrease in the change in symptom burden vs. placebo . Due to a moderate risk of bias and a small size of population, this study´s level of certainty is very low. A positive effect on symptom burden in POTS cannot be ruled out. There was no specific data on the use of Modafinil in ME/CFS. |
| Raj et al. (2005) | Pyridostigmine (Acetylcholineesterase-Inhibitor)  30 mg (single dose) | Upright HR (bpm) | 15 participants (1 RCT study) | 37 ± 11 years | 12 : 3 (80 %) | United States (US) | Very low (ACIP 4) | In adult patients, two hours after administration of 30 mg Pyridostigmine, there was a significant decrease in the standing heart rate vs. placebo (p = 0.160), but there was only a non-significant decrease after hours. Due to a moderate risk of bias and a small size of population, this study´s level of certainty is very low. Pyridostigmine could have a beneficial effect on hemodynamics in POTS. There was no specific data on the use of Pyridostigmine in ME/CFS. |
| Raj et al. (2005) |  | Symptom Burden (Vanderbilt Orthostatic Symptom Scale) | 15 participants (1 RCT study) | 37 ± 11 years | 12 : 3 (80 %) | United States (US) | Very low (ACIP 4) | In adult patients, four hours after the application of 30 mg Pyridostigmine, there is a non-significant decrease in symptom burden vs. placebo (p = 0.174). Due to a moderate risk of bias and a small size of population, this study´s level of certainty is very low. A positive effect on symptom burden in POTS cannot be ruled out. There was no specific data on the use of Pyridostigmine in ME/CFS. |
| Mar et al. (2014) | Selective-Serotonine-Reuptake-Inhibitors (SSRI)  50 mg Sertraline | Change in Heart Rate upon postural change (bpm) | 39 participants (1 RCT study) | 39 ± 9 years | 37 : 2 (95 %) | United States (US) | Very low (ACIP 4) | In adult patients, four hours after the application of sertraline, there is a non-significant decrease in heart rate increment upon postural change vs. placebo. Due to a moderate risk of bias and a small size of population, this study´s level of certainty is very low. A positive effect on hemodynamics in POTS cannot be ruled out. There was no specific data on the use of Sertraline in ME/CFS. |
| Mar et al. (2014) |  | Symptom Burden (Vanderbilt Orthostatic Symptom Scale) | 39 participants (1 RCT study) | 39 ± 9 years | 37 : 2 (95 %) | United States (US) | Very low (ACIP 4) | In adult patients, four hours after the application of sertraline, there is no significant decrease in symptom burden vs. placebo. Due to a moderate risk of bias and a small size of population, and a high risk of publication bias, this study´s level of certainty is very low. A positive effect on symptom burden in POTS cannot be ruled out. There was no specific data on the use of Sertraline in ME/CFS. |
| Smith et al. (2020)  Raj et al. (2009)  Moon et al. (2018) | ß-Adrenergic-Blocking-Agents    Propanolol (20 mg single dose)  OR  Propanolol 10 mg- 20 2x /d  OR  BISOPROLOL 2.5 – 5 mg 1x /d | Change in Heart Rate upon postural change (bpm) | 108 participants  (3 RCT studies*) | (n = 54)  34 ± 10 years  (n = 19)  32 ± 2 years  (n = 19)  39.4 ± 11.6 years  (n = 17)  29.8 ± 9.9 years | 49 : 5 (91 %)  19 : 0 (100 %)  13 : 6 (68 %)  9 : 8 (53 %) | United States (US), South Korea | Very low (ACIP 4) | Two to four hours after the application of a ß-Adrenergic-Blocking-Agent, such as Propanolol, a decrease in heart rate increment could be detected. Four hours after medication, a significant change could be seen compared to the control group.  After three months of ß-Adrenergic-Blocking-Agents, patients showed a significant change in HR increment compared to pre-intervention analysis. Since all included studies comprise a moderate to high risk of bias and a small size of population, there is a very low certainty of evidence. Application of ß-Adrenergic-Blocking-Agents might have a positive effect aon HR increment and could be considered as a treatment option in POTS patients in the long term-use. There was no specific data on the use of ß-Adrenergic-Blocking-Agents in ME/CFS. |
| Smith et al. (2020)  Raj et al. (2009) |  | Symptom Burden (Vanderbilt Orthostatic Symptom Scale) | 55 participants  (2 RCT studies) | (n = 19)  32 ± 2 years  (n = 36)  34 ± 10 years | 49 : 5 (91 %)  19 : 0  (100 %) | United States (US) | Very low (ACIP 4) | In one study, two hours after intervention, a non-significant decrease in Symptom Burden was detected. Another study found a significant decrease in symptom burden after two and four hours. Since all included studies comprise a moderate risk of bias and a small size of population, there is a very low certainty of evidence. A positive effect on symptom burden in POTS cannot be ruled out. There was no specific data on the use of ß-Adrenergic-Blocking-Agents in ME/CFS. |
| Moon et al. (2018) |  | Quality of Life (SF-36) | 36 participants  (1 RCT study*) | (n = 19)  39.4 ± 11.6 years  (n = 17)  29.8 ± 9.9 | 13 : 6 (68 %)  9 : 8 (53 %) | South Korea | Very low (ACIP 4) | In a Pre-Post-analysis, after three months of taking ß-Adrenergic-Blocking-Agents, a significant improvement in physical and mental components could be detected in POTS patients. Due to a high risk of bias and a small size of population, as well as a missing placebo-/standard-of-care comparison, the certainty of evidence is very low. ß-Adrenergic-Blocking-Agents might have a positive effect on Quality of Life in POTS patients in the long term use. There was no specific data on the use of ß-Adrenergic-Blocking-Agents in ME/CFS. |

*one study (Moon, 2018) was analysed by using a Pre-Post-Intervention-approach since Placebo- or Standard-of-Care- data was not available.

**Summary of Findings – Non-pharmacological interventions compared to Placebo or Standard-of-Care in patients with POTS (RCT)**

**Non-pharmacological interventions compared to Placebo or Standard-of-Care in Patients with POTS (RCT studies)**

**Population:** Adults and / or children with a confirmed diagnosis of POTS

**Intervention:** Non-pharmacological interventions (see below)

**Comparison:** Placebo or Standard-of-Care

| Studies | Intervention (+ Dosage) | Outcome | Number of participants included in the analysis  (studies) | Age of participants  (MEAN + SD or Age Range) | Sex Ratio (female : male; percentage of females) | Country | Certainty of evidence (GRADE) | Comments |
| --- | --- | --- | --- | --- | --- | --- | --- | --- |
| Bourne et al. (2021)  Smith et al. (2020) | **Compression Garments**  (Abdominal Compression, Leg Compression)  [20 – 40 mmHg] | Change in HR upon postural change (bpm) | 49 participants (2 studies) | 18 – 60 years (n=30)  32 ± 2 years (n = 19) | 28 : 2  (93%)  19 : 0  (100 %) | United States (US),  Canada | Very low (ACIP 4) | In adult patients, abdominal compression alone caused a non-significant change in HR increment upon postural change compared to placebo after two hours of application. With additional leg compression, a significant decrease in HR increment could be detected upon postural change (p < 0.001) vs. no compression garments. Due to concerns regarding risk of bias, the impact of patients` awareness and a small size of the study population, certainty of evidence is very low. Compression garments might have a beneficial effect on HR increment and could be considered as a treatment option in POTS patients. There was no specific data on the use of compression garments in patients with ME/CFS. |
| Bourne et al. (2021)  Smith et al. (2020) |  | Symptom Burden (Vanderbilt Orthostatic Symptom Scale) | 49 participants (2 RCT studies) | 18 – 60 years(n=30)  32 ± 2 years (n = 19) | 28 : 2  (93%)  19 : 0  (100 %) | United States (US),  Canada | Very low (ACIP 4) | In adult patients, abdominal compression did not cause a significant change in symptom burden compared to placebo after two hours of application. With additional leg compression, a significant decrease in symptom burden (p < 0.001) could be detected compared to wearing no compression garments. Compression garments might have a beneficial effect on symptom burden and could be considered as a treatment option in POTS patients. There was no specific data on the use of compression garments in ME/CFS. |
| Gamboa et al. (2015) | **Impedance Treshold Device (ITD), Breathing Device** | Upright Heart Rate (bpm) | 26 participants (1 RCT study) | 30 ± 2 years | 25 : 1  (96 %) | United States (US) | Very low (ACIP 4) | In adult patients, the use of an impedance treshold device (ITD) caused a significant decrease in upright heart rate in comparison to the use of a sham device. Due to concerns regarding the risk of bias and due to the small size of study population, the level of certainty for the use of ITD is very low. By enhancing venous return, an ITD might have a beneficial effect on upright heart rate in POTS patients but can currently not be generally recommended. There was no specific data on the use of Impendance Treshold Devices in ME/CFS. |
| Gamboa et al. (2015) |  | Symptom Burden (Vanderbilt Orthostatic Symptom Scale) | 26 participants (1 RCT study) | 30 ± 2 years | 25 : 1  (96 %) | United States (US) | Very low (ACIP 4) | In adult patients, the use of an impedance treshold device (ITD) did so far not cause any significant decrease in symptom burden in comparison to the use of a sham device. Due to concerns regarding the risk of bias and due to a small study population, the level of certainty for the use of ITD is very low. An effect on symptom burden cannot be ruled out. There was no specific data on the use of Impedance Treshold Devices in ME/CFS. |
| Nardone et al. (2020) | Neck Compression | Upright Heart Rate (bpm) | 10 participants (1 RCT study) | 40 ± 10 years | 9 : 1  (90 %) | Canada | Very low (ACIP 4) | In adult patients, neck compression did not show a significant change in upright heart rate. Since there is only one study which included very few participants and comprises concerns regarding risk of bias, certainty of evidence is very low. A beneficial effect on heart rate increment upon postural change cannot be ruled out. There was no specific data on the use neck compression garments in ME/CFS. |
| Nardone et al. (2020) |  | Symptom Burden (Vanderbilt Orthostatic Symptom Scale) | 10 participants (1 RCT study) | 40 ± 10 years | 9 : 1  (90 %) | Canada | Very low (ACIP 4) | In adult patients, neck compression led to a significant change in symptom burden (p = 0.04). Since this study included very few participants and comprises concerns regarding risk of bias, especially patients´ awareness, certainty of evidence is very low. A beneficial effect on symptom burden in POTS patients cannot be ruled out. There was no specific data on the use of neck compression garments in ME/CFS. |
| Wheatley-Guy et al. (2023) | **Physical exercise** | Symptom Burden (COMPASS-31) | 49 participants (1 RCT study) | Intervention group (n = 26):  33 ± 11 years  Control group  (n= 23):  37 ± 10 years | Intervention group:  23 : 3  (88.5 %)  Control group:  22 : 1  (95.7 %) | United States (US) | Very low (ACIP 4) | In adult patients who participated in a partly supervised exercise programme over three months, no significant changes in symptom burden could be detected vs. a standard-of-care group. Since there is only one RCT study and due to a moderate risk of bias, a high dependence on patient compliance and a small number of participants, certainty of evidence is very low. Physical exercise might have a beneficial effect on symptom burden in POTS patients. As it can be individually adapted, it could be considered as a possible treatment option. There was no specific data on the use of physical training approaches in ME/CFS. |
| Garland et al. (2021) | **Salt supplementation**  **High Na+ Diet (300 mEq / day)** | Change in HR upon postural change (bpm) | 27 participants (1 RCT study) | POTS: 35 ± 8 years  Healthy controls: 31 ± 6 years | POTS patients:  14 : 0  (100 %)  Healthy controls:  13 : 0  (100 %) | United States (US) | Very low (ACIP 4) | In adult patients performing a high sodium diet for six days, a significant change in HR increment upon postural change could be detected compared to a control group with low sodium diet. Since there is only one RCT study, and due to a moderate risk of bias and a small size of the study population, certainty of evidence is very low for this study. There might be a beneficial effect of salt supplementation on hemodynamics of POTS patients. Due to its simple applicability, it could be considered as a possible treatment option. There was no specific data on the use of salt supplementation in ME/CFS |
| Stavrakis et al. (2024) | **Transdermal Vagus Nerve Stimulation (tVNS)**  1 hour per day (Amplitude :1 mA below discomfort threshold.  20 Hz, pulse width of 200 ms) | Change in HR upon postural change (bpm) | 25 participants (1 RCT study) | Intervention group (n = 11): 31.2 ± 5.5 years  Control group (n = 14): 30.8 ± 5.6 years | 25 : 0  (100 %) | United States (US) | Very low (ACIP 4) | In adult patients undergoing transdermal vagal nerve stimulation over a period of two months, a significant decrease in HR increment compared to the control group using a sham device could be detected. Since there is only one RCT study and due to a small number of study participants and a moderate risk of bias, the level of certainty is very low. tVNS might have a beneficial effect on hemodynamics in POTS patients in the long term. There was no specific data on the use of tVNS in ME/CFS. |
| Stavrakis et al. (2024) |  | Symptom Burden (COMPASS-31) | 25 participants (1 RCT study) | Intervention group (n = 11): 31.2 ± 5.5 years  Control group (n = 14): 30.8 ± 5.6 years | 25 : 0  (100 %) | United States (US) | Very low (ACIP 4) | In adult patients undergoing transdermal vagal nerve stimulation over a period of two months, there was no significant decrease in symptom burden compared to the control group using a sham device. Since there is only one RCT study and due to a small number of study participants and a moderate risk of bias, the level of certainty is very low. A beneficial effect of tVNS on symptom burden in POTS patients in the long term cannot be ruled out. There was no specific data on the use of tVNS in ME/CFS. |
