# Supplementary material for: Systematic literature review: treatment of postural orthostatic tachycardia syndrome (POTS)
Source: Clin Auton Res. 2025 Nov 12;36(1):3–16. doi: 10.1007/s10286-025-01172-2 (PMC12982215; doi:10.1007/s10286-025-01172-2)
Supplement: Supplementary file 8 — Supplementary file8 (DOCX 42 kb) [file 10286_2025_1172_MOESM8_ESM.docx]

**Supplement 8 - SUMMARY OF FINDINGS (Non-RCT)**

**Summary of findings – Pharmacological interventions in Patients with POTS (Non-RCT studies)**

**Pharmacological interventions evaluated in a pre- and post-intervention analysis or compared to a non-randomized control group**

**Population:** Adults and / or children with a confirmed diagnosis of POTS

**Intervention:** Pharmacological interventions (see below)

**Comparison:** Pre-intervention assessments; baseline data

| Studies | Intervention (+ Dosage) | Outcome | Number of participants (studies) | Age of participants  (MEAN + SD or Age Range) | Sex Ratio (female : male; percentage of females) | Country | Certainty of evidence (GRADE) | Comments |
| --- | --- | --- | --- | --- | --- | --- | --- | --- |
| Nesheiwat et al. (2020) | **Buproprion**  **150 mg – 450 mg od** | Upright HR (bpm) | 47 participants (1 study) | 42.0 ± 13.6 years | 38 : 9  (80.1 %) | United States (US) | Very low (ACIP 4) | Buproprion has not shown to cause any significant changes in upright heart rate. Since there is only one study which uses a retrospective approach, and due to a certain risk of bias and a small study population, certainty of evidence is very low. A potential effect of buproprion in POTS patients cannot be ruled out. There was no specific data on the use of Buproprion in ME/CFS. |
| Gordon et al. (2000)  Jacob et al. (1997) | **Clonidine**  **0.1 - 0.2 mg (single dose)** | Upright HR (bpm) | 19 participants (2 studies) | 14 - 39 years (n = 6)  18 – 47 years (n = 13) | not clearly presented  11 : 2 (85 %) | United States (US) | Very low (ACIP 4) | In adult POTS patients, short-term application of Clonidine did not lead to a significant decrease in heart rate increment upon postural change or upright HR. Due to a very small size of population and high risk of impecision, certainty of evidence is very low. A potential effect of Clonidine on hemodynamics in POTS cannot be ruled out. There was no specific data on the use of Clonidine in ME/CFS. |
| Stewart et al. (2021) | **Digoxin**  **500 mg** | Upright HR (bpm) | 36 participants (1 study) | 15 – 30 years | 36 : 0 (100 %) | United States (US) | Very low (ACIP 4) | In patients with POTS, short-term application of Digoxin did not lead to significant changes in upright heart rate compared to untreated POTS. Due to a small size of population and certain concerns regarding the risk of bias, certainty of evidence is very low. A potential effect on hemodynamics cannot be ruled out. There was no specific data on the use of Digoxin in ME/CFS. |
| Ruzieh et al. (2017) | **Droxidopa**  **100 – 600 mg 3x/d**  **[MAX. 1800 mg]** | Upright HR (bpm) | 37 participants (1 study) | 48.08 ± 18.1 years | 28 : 9 (75.7 %) | United States (US) | Very low (ACIP 4) | Application of droxidopa over up to one year did not lead to a significant decrease in standing heart rate in POTS patients. Since there is only one non-randomized study which uses a retrospective approach with a small size of population and a certain risk of bias, certainty of evidence is very low. A potential effect of droxidopa in POTS patients cannot be ruled out. There was no specific data on the use of Droxidopa in ME/CFS. |
| Kanjwal et al. (2012) | **Erythropoetin**  **10.000 – 20.000 I.E./week if Hct < 50 %** | Upright HR (bpm) | 39 participants (1 study) | 33 ± 12 years | 37 : 2 (95 %) | United States (US) | Very low (ACIP 4) | In adult patients, application of Erythropoetin over six months did not lead to any significant changes in upright heart rate. Since there is only one study which uses a retrospective approach and due to a small size of population and a certain risk of bias, certainty of evidence is very low. A potential effect of Erythropoetin cannot be ruled out. There was no specific data on the use of Erythropoetin in ME/CFS. |
| Towheed et al. (2020)  Ruzieh et al. (2017)  Barzilai et al. (2015) | Ivabradine  2.5 – 10 mg 2x / d  7.5 mg (single dose)  MAX. (children):  7.5 mg 2x/d | Upright Heart Rate (bpm) | 84 participants (3 studies) | 31 ± 3 years (n = 8)  35.1 ± 10.35 years (n = 49)  12 – 17 years (n = 27) | 6 : 2 (75 %)  47 : 2 (95.9 %)  25 : 2 (92.5 %) | Israel, United States (US) | Very low (ACIP 4) | In children and adults receiving Ivabradine for 3-12 months, a significant change in upright heart rate could be detected. Short-term application of Ivabradine led to a significant change in upright heart rate. Since there are few studies and a small size of each study population as well as a certain risk of bias, certainty of evidence is very low. Ivabradine might be a suitable treatment option to improve hemodynamics in POTS patients, including children. There was no specific data on the use of Ivabradine in ME/CFS. |
| Gordon et al. (2000) | **Midodrine**  **2.5 mg 1x/d (children)**  **10 mg (single dose)** | Upright Heart Rate (bpm) | 12 participants (1 study) | 14 – 39 years (n = 12) | Not clearly presented | United States (US) | Very low (ACIP 4) | In children suffering from POTS, application of Midodrine hydrochloride over three months led to a significant decrease in HR increment upon postural change compared to the control group. In adult POTS patients, short-term application of Midodrine partly led to a significant change in HR increment. Since the studies are non-randomized trials with certain concerns regarding risk of bias and due to a small size of the study population, certainty of evidence is very low. Midodrine hydrochloride might have a beneficial effect on hemodynamics in POTS patients, including children and could be considered as a treatment option. There was no specific data on the use of Midodrine in ME/CFS. |
| Yang et al. (2013)  Zhang et al. (2012)  Chen et al. (2011)  Hoeldtke et al. (2006)  Jacob et al. (1997) |  | Change in Heart Rate upon postural change (bpm) | 119 participants (5 studies) | 20 – 54 years (n = 15)  6 – 17 years (n = 19)  18 – 47 years (n = 13)  11 ± 3 years  (n = 44)  11.5 ± 2.5 years (n = 28) | Not clearly presented  Not clearly presented  11 : 2 (85 %)  16 : 12 (57.1 %)  Not clearly presented | United States (US), China | Very low (ACIP 4) |  |
| Hoeldtke et al. (2006) | **Octreotide**  **0.9 µg/kg s.c.** | Upright HR (bpm) | 9 participants (1 study) | 20 – 54 years (n = 9) | Not clearly presented | United States (US | Very low (ACIP 4) | In adult POTS patients, short-term application of Octreotide led to a significant change in upright heart rate. Due to a very small size of population and high risk of impecision, certainty of evidence is very low. Octreotide might have a beneficial effect on hemodynamics in POTS. There was no specific data on the use of Octreotide in ME/CFS. |
| Gordon et al. (2000) | **Phenobarbital**  **120 mg (single dose)** | Upright HR (bpm) | 11 participants (1 study) | 14 – 39 years | Not clearly presented | United States (US) | Very low (ACIP 4) | In patients with POTS, short-term application of Phenobarbital did not lead to significant changes in upright heart rate compared to pooled baseline data. Due to a very small number of participants, and certain concerns regarding risk of bias, the certainty of evidence is very low. A positive effect of Phenobarbital on hemodynamics in POTS cannot be ruled out. There was no specific data on the use of Phenobarbital in ME/CFS. |
| Stewart et al. (2021)  Kanjwal et al. (2011) | **Pyridostigmine**  **60 mg (single dose)** | Upright HR (bpm) | 206 participants (2 studies) | 15 – 30 years (n = 36)  26 ± 12 years (n = 170) | 36 : 0 (100 %)  (not clearly presented) | United States (US) | Very low (ACIP 4) | In patients with POTS, short-term and long-term application of Pyridostigmine led to a significant decrease in upright heart rate. Since the studies are non-randomized trials with a risk of imprecision and some concerns regarding the risk-of-bias assessment, certainty of evidence is very low. Pyridostigmine might be a suitable treatment option to improve hemodynamics in patients with POTS, including children. There was no specific data on the use of Pyridostigmine in ME/CFS. |
| Zhao et al. (2014)  Chen et al. (2011) | ß**-Adrenergic-Blocking-Agents**  **Propanolol 40 mg (single dose)**  **Bisoprolol 5 mg 1x/d**  **Metoprolol 0.5 mg twice daily) (children)** | Change in Heart Rate upon postural change (bpm) | 68 participants (2 studies) | 7- 16 years (n = 49)  6 – 17 years (n = 19) | 24 : 25 (49 %)  Not clearly presented | China | Very low (ACIP 4) | In children, the application of a ß-Adrenergic-Blocking-Agent over a period of 1.5 to 6 months, resulted in a significant decrease in HR increment upon postural change compared to baseline data. In adult patients with POTS, short-term application of ß-Adrenergic-Blocking-Agents, as well as application of ß-Adrenergic-Blocking-Agents over 3 months led to a significant decrease in upright heart rate compared to baseline data. Since the studies were non randomized trials and due to a small size of study population and a certain risk of bias, the level of certainty is very low. ß-Adrenergic Blocking Agents might have a beneficial effect on hemodynamics in POTS patients including children and could be considered a suitable treatment option. There was no specific data on the use of ß-Adrenergic-Blocking-Agents in ME/CFS. |
| Gordon et al. (2000)  Freitas et al. (2000) |  | Upright Heart Rate (bpm) | 19 participants  (2 studies) | 14 – 39 years (n = 9)  17 - 57 years ( n = 10) | Not clearly presented  10 : 0 (100 %) | United States (US), Portugal | Very low (ACIP 4) |  |

**Summary of findings – Non-pharmacological interventions in Patients with POTS (Non-RCT studies)**

**Non-pharmacological interventions evaluated in a pre- and post-intervention analysis or compared to a non-randomized control group**

**Population:** Adults and / or children with a confirmed diagnosis of POTS

**Intervention:** Non-pharmacological interventions (see below)

**Comparison:** Pre-intervention assessments; baseline data

| Studies | Intervention | Outcome | Number of participants  (studies) | Age of participants  (MEAN + SD or Age Range) | Sex Ratio (female : male; percentage of females) | Country | Certainty of evidence (GRADE) | Comments |
| --- | --- | --- | --- | --- | --- | --- | --- | --- |
| Heyer et al. (2014) | **Compression garments**  **20 – 40 mmHg** | Change in HR upon postural change | 20 participants (1 study) | Mean age: 15. 8 years  [13 – 19 years] | 19 : 1 (95 %) | United States (US) | Very low (ACIP 4) | In children, short term application of lower-extremity and abdominal compression garments led to a significant decrease in heart rate increment upon postural change. Due to a moderate risk of bias in this non-randomized trial and due to a small size of the study population, level of certainty is very low. Compression garments might have a positive effect on hemodynamics in POTS and could be considered as a potential treatment option. There was no specific data on the use of compression garments in ME/CFS. |
| Zha et al. (2023) | **Gluten-free Diet** | Symptom Burden (COMPASS-31) | 20 participants (1 study) | 16 – 62 years | 20 : 0 (100%) | United States (US) | Very low (ACIP 4) | In mainly adult patients, performing a gluten-free diet for four weeks led to a significant decrease in symptom burden. Due to a high risk of bias and a small size of the study population, certainty of evidence is very low. A Gluten-free diet might have a positive impact on symptom burden in POTS patients. There was no specific data on the use of gluten-free dietary approaches in ME/CFS. |
| Rodriguez et al. (2022) | **Oral water intake**  **450 – 500 ml water or clear soup (oral)** | Upright Heart Rate (bpm) | 28 participants (3 studies) | 27.62 ± 5.95 (n = 13) | 11 : 2 (85 %)  Not clearly presented | Switzerland | Very low (ACIP 4) | In adult patients, short-term hydration with mineral water or clear soup did not lead to significant changes in heart rate increment upon postural change. Due to the design as non-randomized trials with a very small size of population, level of certainty is very low. Due to its simple applicability, oral hydration could be considered as a possible treatment option. There was no specific data on the use of increased oral water intake in patients with ME/CFS. |
| Rodriguez et al. (2019)  Z´Graggen et al. (2010) |  | Change in Heart Rate (bpm) |  | 37.7 ± 6 years (n = 7)  18 – 45 years (n = 8) | 7 : 0 (100%)  6 : 2 (75 %) |  | Very low (ACIP 4) |  |
| Gordon et al. (2000)  Jacob et al. (1997)  Ruzieh et al. (2017) | **1 Liter Saline (intravenous)** | Change in Heart Rate (bpm) | 81 participants (3 studies) | 14 – 39 years (n = 11)  18 – 47 years (n = 13)  35.0 ± 12.9 years | Not clearly presented  11 : 2 (85 %)  55 : 2 (96.5 %) | United States (US) | Very low (ACIP 4) | In adolescents and adult patients, short-term , as well as long-term intermittend intravenous hydration with saline led to significant decrease in heart rate increment. Due to the design as non-randomized trials with a very small size of population, level of certainty is very low. Intravenous saline might have a positive effect on hemodynamics in POTS in the long term but is an invasive method that carries risks and can currently not be recommended for POTS patients. The effect of oral rehydration, especially in the long term remains unclear. There was no specific data on the use of intravenous saline infusions in ME/CFS. |
| Ruzieh et al. (2017) | **1 Liter saline infusions** | Quality of Life (SF-36) | 57 participants (1 study) | 35.0 ± 12.9 years | 55 : 2 (96.5 %) | United States (US) | Very low (ACIP 4) | In adult patients, intermittent saline infusions over 3-12 months led to a significant improvement in quality of life. Since there is only one study, designed as a non-randomized trial with few participants and a high risk of bias, certainty of evidence is very low. Intravenous saline might have a positive effect on symptom burden in POTS in the long term but is an invasive method that carries risks and can currently not be recommended for POTS patients. There was no specific data on the use of intravenous saline infusions in patients with ME/CFS. |
| Gibbons et al. (2021) | **Physical training**  **6 months** | Change in HR upon postural change (bpm) | 48 participants (1 study) | 26.3 ± 6.6 years | 43 : 5 (89.6 %) | United States (US) | Very low (ACIP 4) | Adult patients who completed a physical training programme over 3 – 6 months, showed a significant decrease in upright heart rate and change in HR upon postural change. Due to the study design as non-randomized trials with few participants, certainty of evidence is very low. Physical exercise might have a beneficial effect on hemodynamics in POTS and could be considered as a possible treatment option, as it is individually adaptable and easy to apply. There was no specific data on the use of physical training as a therapeutic approach in ME/CFS. |
| Shibata et al. (2012)  Fu et al. (2010) | **Physical training**  **3 months** | Upright HR (bpm) | 19 participants (2 studies) | 27 ± 2 years | 18 : 1 (95 %) | United States (US) | Very low (ACIP 4) |  |
| Svensson et al. (2024) | **Physical training**  **3 months** | Symptom Burden (Vanderbilt Orthostatic Symptom Scale) | 25 participants (1 study) | [21 – 55 years] | 21 : 4 (84 %) | Sweden | Very low (ACIP 4) | Adult patients participating in a three-month supervised physical exercise programme showed a significant decrease in symptom burden compared to baseline. Since this is a non-randomized trial with few participants and a certain risk of bias, level of certainty is very low. Physical exercise might have a positive impact on symptom burden in POTS and could be considered as a possible treatment option, as it is individually adaptable and easy to apply. There was no specific data on the use of physical training as a therapeutic approach in ME/CFS. |
| George et al. (2016) | **Physical training**  **3 months** | Quality of Life (SF-36) | 78 participants (1 study) | 26 ± 11 years | Not clearly presented | United States (US) | Very low (ACIP 4) | Adult patients who completed a physical training programme over 3 months, showed a significant improvement in physical, mental and social components of life quality. Since this was a non-randomized trial with few participants, certainty of evidence is very low. Physical exercise might have a beneficial effect on quality of life in POTS and could be considered as a possible treatment option, as it is individually adaptable and easy to apply. There was no specific data on the use of physical training as a therapeutic approach in ME/CFS. |
